# Supplementary material for: Seasonal variations in haematological and biochemical parameters of healthy Gambian adults: Retrospective study 2018–2022
Source: PLOS Glob Public Health. 2024 Sep 17;4(9):e0003715. doi: 10.1371/journal.pgph.0003715 (PMC11407651; doi:10.1371/journal.pgph.0003715)
Supplement: S1 Text — (PDF) [file pgph.0003715.s002.pdf]

C/o MRC Unit The Gambia @ LSHTM, Fajara
P.O. Box 273, Banjul
The Gambia, West Africa
Fax: +220 – 4495919 or 4496513

The Gambia Government/MRCG Joint
Tel: +220 – 4495442-6 Ext. 2308

ETHICS COMMITTEE

Email: ethics@mrc.gm

20 September 2023

Mr Mustapha Dibbasey
MRCG at LSHTM

Dear Mr Dibbasey

Project ID/Ethics ref: L2023.E03

Project Title: Seasonal and yearly variation in haematological and biochemical parameters of healthy Gambian adults: A Retrospective Study

Thank you for submitting your application which was considered by the Gambia Government/MRCG Joint Ethics Committee at its meeting held on 31 August 2023.

Confirmation of Ethical Opinion
The committte is pleased to approve your request for ethical approval waiver on the basis of the application and supporting document.

The documents reviewed were:

Document Type/Name	Version Number	Date
Request letter		10/08/2023
Draft manuscript detailing the procedure & how the study was conducted		

With best wishes
Yours sincerely

Dr Mohammadou Kabir Cham
Chairperson, Gambia Government/MRCG Joint Ethics Committee
