# Supplementary figures and images for: Seasonal variations in haematological and biochemical parameters of healthy Gambian adults: Retrospective study 2018–2022
Source: PLOS Glob Public Health. 2024 Sep 17;4(9):e0003715. doi: 10.1371/journal.pgph.0003715 (PMC11407651; doi:10.1371/journal.pgph.0003715)

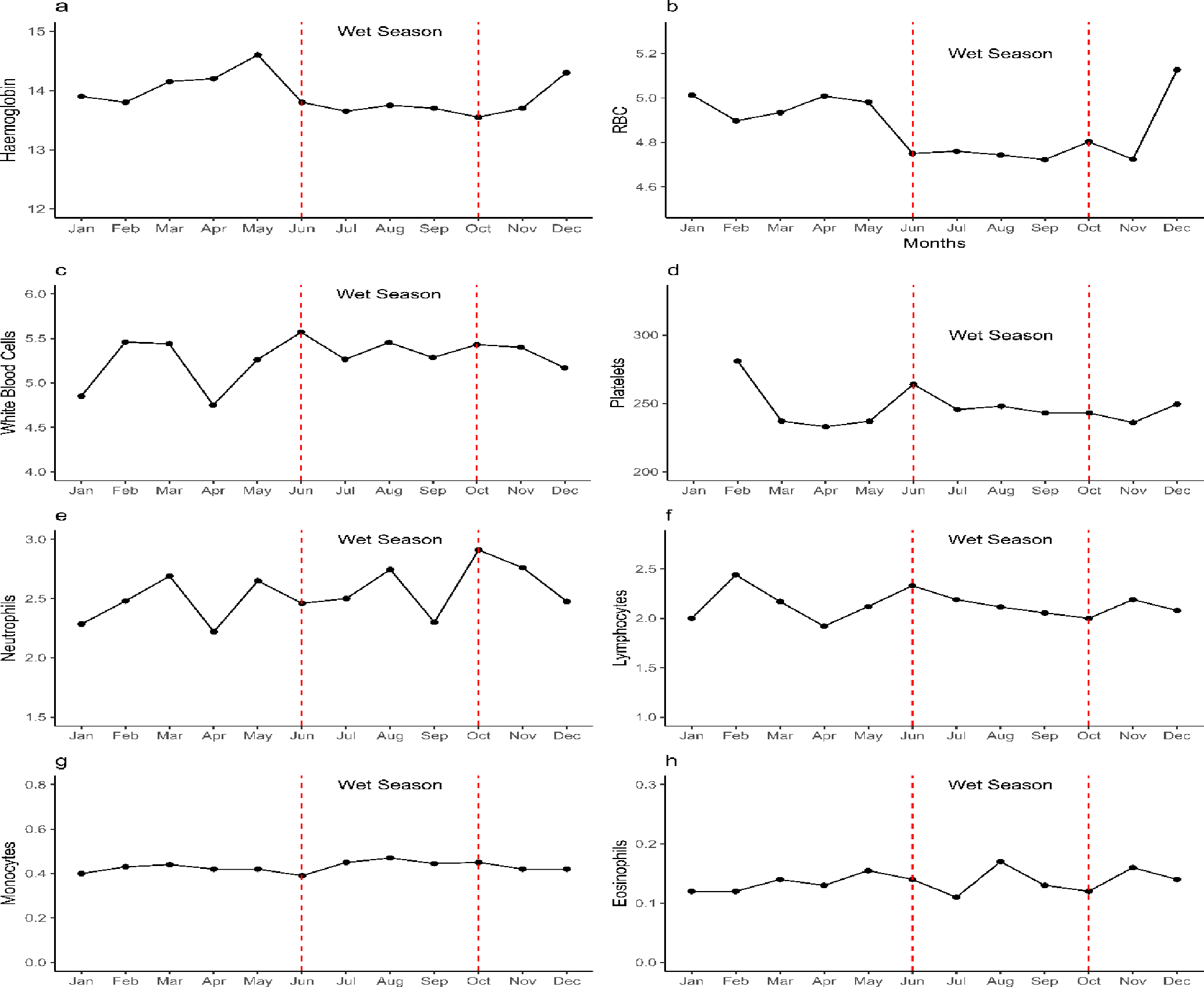

Supplement: S1 Fig — The point showed monthly medians in five year period. (TIF) [file pgph.0003715.s003.tif]

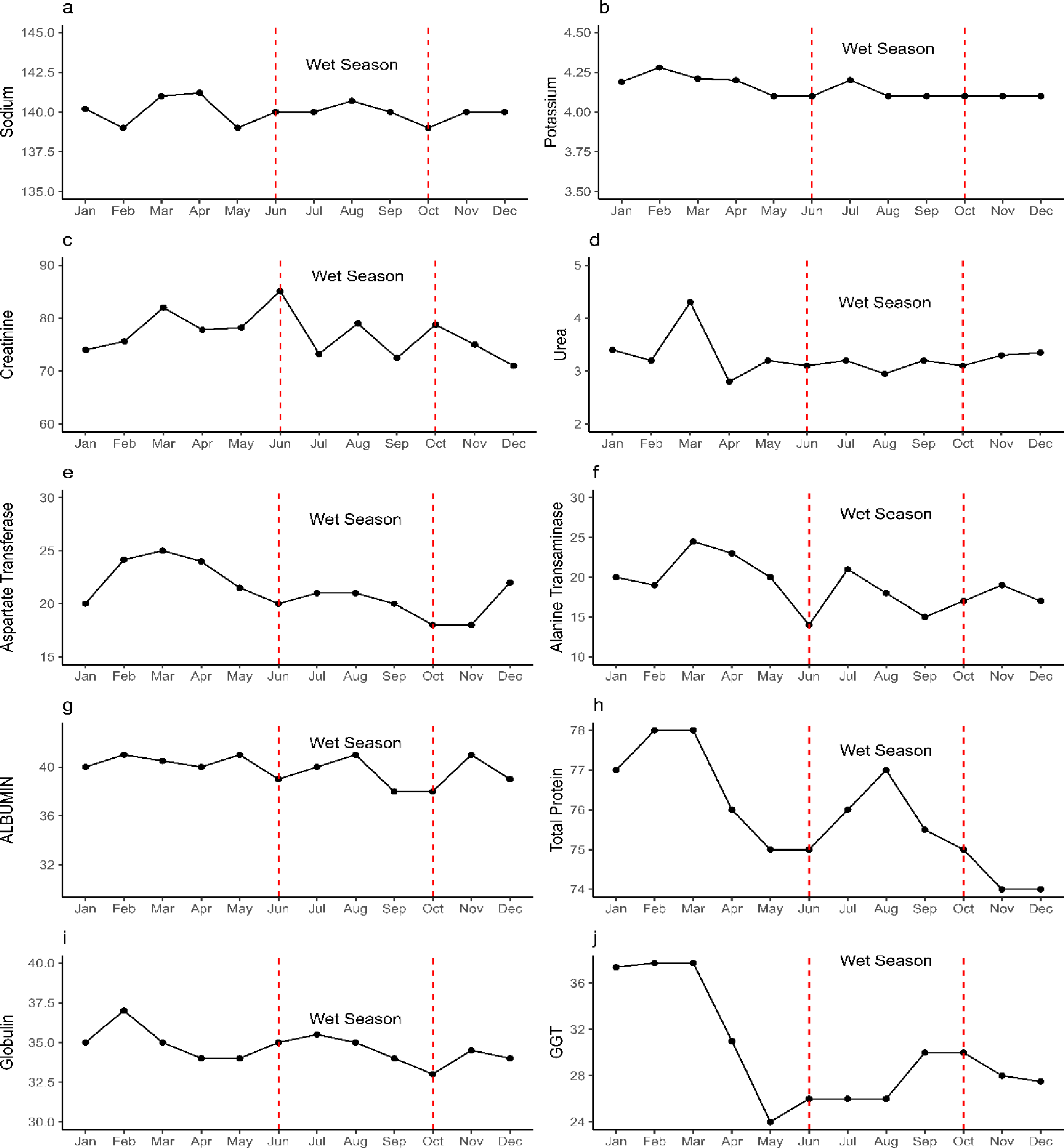

Supplement: S2 Fig — The point showed monthly medians in five year period. (TIF) [file pgph.0003715.s004.tif]
