# Supplementary material for: Seasonal variations in haematological and biochemical parameters of healthy Gambian adults: Retrospective study 2018–2022
Source: PLOS Glob Public Health. 2024 Sep 17;4(9):e0003715. doi: 10.1371/journal.pgph.0003715 (PMC11407651; doi:10.1371/journal.pgph.0003715)
Supplement: Attachment — Submitted filename: Comment.pdf [file pgph.0003715.s005.pdf]

Title: "**Seasonal and yearly variations in haematological and biochemical parameters of healthy Gambian adults: a retrospective study**"

<u>Summary</u>

The study attempts to establish or otherwise the influence of seasonal variation on hematological and biochemical parameters. It reports that, reference ranges are not affected by seasonal variation however, HB, RBC, liver enzymes as well potassium were seasonally dependent.

<u>General comment</u>

The study requires major modification. The use of the term/word healthy people is problematic. If someone came to the laboratory for routine check-up/testing does not make such individual healthy person. Perhaps, individual with no known underlying health condition could suffice. Although the study design was retrospective, reading through the entire write-up appears more prospective.

<u>Point by point comment</u>

<u>Methods</u>

Line 90 – 93 is not clear. Please revise

Line 96, we are being encouraged not to start sentences with abbreviation

Line 104 -108, The definition of healthy people is challenging

Line 108, statement suggest more prospective design

Line 113 – 114, it is not clear whether the study included persons aged 18 – 63 or exempted these age group rather

Line 117 (the figure 1), is confusing. What is Hep? What is the meaning of Age (18-63) = 129? The entire figure 1 is not clear.

Line 120 – 130, this section appears the authors did the sampling themselves

Line 157 is contradicting with line 103

<u>Discussion</u>

This section needs major revision. It is not clear why the authors are comparing their study to a previous work among children less then 5 years (line 264 -266). It is also not clear if the study evaluated the malaria or other parasitic infectious state of the study participants. It can be argued that, during the raining season, parasitic infection is high and can affect the hematological parameters. Therefore, the observed variation in the parameters require clear explanation. Why

was hematocrit (HCT) not included to substantiated the hemoglobin (Hb) recorded. It is possible for people to take less fluid during the raining season and get dehydrated.
