# Supplementary material for: Seasonal variations in haematological and biochemical parameters of healthy Gambian adults: Retrospective study 2018–2022
Source: PLOS Glob Public Health. 2024 Sep 17;4(9):e0003715. doi: 10.1371/journal.pgph.0003715 (PMC11407651; doi:10.1371/journal.pgph.0003715)
Supplement: Attachment — Submitted filename: PGPH-D-23-02498.pdf [file pgph.0003715.s006.pdf]

PLOS Global Public Health

Seasonal and yearly variations in haematological and biochemical parameters of healthy Gambian adults: a retrospective study

--Manuscript Draft--

**Manuscript Number:**	PGPH-D-23-02498
**Article Type:**	Research Article
**Full Title:**	Seasonal and yearly variations in haematological and biochemical parameters of healthy Gambian adults: a retrospective study
**Short Title:**	Seasonal and yearly variations in the routine haematological and biochemical parameters
**Corresponding Author:**	Mustapha Dibbasey, BSc (honours); MSc<br>University of Ghana<br>Accra, GHANA
**Order of Authors:**	Mustapha Dibbasey, BSc (honours); MSc
	Mamudou Dahaba
	Francess Sarfo
	Rosyna Begum
	Mustapha Kanteh
	Nyima Sumareh
	Mustapha Bakare
	Solomon Umukoro
	Alfred Amambua-Ngwa
**Keywords:**	Keywords: Dry Season; Wet Season; Haematological Parameters; Biochemical Parameters
**Abstract:**	Background: Haematological and biochemical parameters are important biomarkers for assessing the overall health of an individual, and several factors such as environmental changes and pandemic-related life style changes can contribute to seasonal and yearly variation. In this study, we determined the presence and extent of seasonal variations and provided further insight into the yearly trend from 2018 to 2022 in a comprehensive set of routine haematological and biochemical parameters in healthy adults in Gambia.<br>Methods: The study retrieved five years of data (January 2018 to December 2022) from an electronic database and analysed 503 full blood counts and 772 biochemical data.<br>Results: The study found an association between seasonality and haematological parameters such as HB, RBC, WBC and neutrophils but seasonal effects on reference intervals from dry season to wet season were not significant (P>0.05). In fact, our study showed comparable reference intervals between our study and a study that targeted healthy Gambian Adults during TB contact tracing. The median values for HB and RBC as well as liver enzymes and potassium were high in the wet season but low in dry season. Although no significant difference was found (Kruskal-Wallis chi-squared = 0.1148, p-value = 1) among the median values for all the parameters from 2018 to 2022, further analysis showed reduced median values for HB and RBC and a high median value for total WBC in 2022, with ALT and AST parameters showing a consistent declining trend from 2018 to 2022.<br>Conclusion: In this study, we found no significant seasonal effects on the reference intervals of haematological and biochemical parameters. However, changes in patterns in certain parameters, such as HB, RBC, liver enzymes, and potassium, from January to December were profoundly recognised. This suggests that seasonal variations should be considered when interpreting laboratory results and conducting future reference interval studies. Further assessment of the declining trend in AST and ALT levels from 2018 to 2022 should be conducted.

Powered by Editorial Manager® and ProduXion Manager® from Aries Systems Corporation

Additional Information:	
Question	Response
**Financial Disclosure**<br><br>Enter a financial disclosure statement that describes the sources of funding for the work included in this submission and the role the funder(s) played. This includes grants and any commercial funding of the work or authors.<br><br>This statement will be typeset if the manuscript is accepted for publication.<br><br>*Please review the submission guidelines and the instructions link below for detailed requirements and guidance.*	The study was funded by Medical Research Council the Gambia at London School of Hygiene and Tropical Medicine
**Competing Interests**<br><br>On behalf of all authors, disclose any competing interests that could be perceived to bias this work.<br><br>This statement will be typeset if the manuscript is accepted for publication.<br><br>*Please review the instructions link below and PLOS Global Public Health's competing interests policy to determine what information must be disclosed at submission.*	The authors declare no competing interest
**Data Availability**<br><br>Before publication, Authors are required to make fully available and without restriction all data underlying their findings. Please see our PLOS Data Policy page for detailed information on this policy.<br><br>A **Data Availability Statement**, detailing	The data for the study is fully available upon request

where the data can be accessed, is required at first submission. Insert your Data Availability Statement in the box below.<br><br>Please see the data reporting section of our submission guidelines for instructions on what you need to include in your Data Availability Statement.<br><br>This statement will be typeset if the manuscript is accepted for publication.<br><br>*PLOS allows rare exemptions to address legal and ethical concerns. If you have legal or ethical restrictions, please use the box below to detail these in full sentences for the Journal team to consider.*	

Powered by Editorial Manager® and ProduXion Manager® from Aries Systems Corporation

Manuscript

Click here to access/download;Manuscript;Mustapha_Dibbasey_SV_Manus

Seasonal and yearly variations in haematological and biochemical parameters of healthy Gambian adults: A Retrospective Study

Mustapha Dibbasey*[1,2], Mamudou Dahaba[1], Francess Sarfo[1], Rosyna Begum[1], Mustapha Kanteh[1], Nyima Sumareh[1], Mustapha Bakare[1], Solomon Umukoro[1], **Alfred Amambua-Ngwa**[1,3]

[1]Medical Research Council the Gambia at London School of Hygiene and Tropical Medicine

[2]Department of Biochemistry, University of Ghana, Ghana, Accra, Legon

[3]London School of Hygiene and Tropical Medicine

*Corresponding Author: Mustapha Dibbasey (lshmd4@lshtm.ac.uk; mdibbasey@mrc.gm)

Abstract

Background: Haematological and biochemical parameters are important biomarkers for assessing the overall health of an individual, and several factors such as environmental changes and pandemic-related life style changes can contribute to seasonal and yearly variation. In this study, we determined the presence and extent of seasonal variations and provided further insight into the yearly trend from 2018 to 2022 in a comprehensive set of routine haematological and biochemical parameters in healthy adults in Gambia.

Methods: The study retrieved five years of data (January 2018 to December 2022) from an electronic database and analysed 503 full blood counts and 772 biochemical data.

Results: The study found an association between seasonality and laboratory parameters such as HB, RBC, WBC and neutrophils but seasonal effects on reference intervals from dry season to wet season were not significant ($P>0.05$). In fact, our study showed comparable reference intervals between our study and a study that targeted healthy Gambian Adults during TB contact tracing. The median values for HB and RBC as well as liver enzymes and potassium were high in the wet season but low in dry season. Although no significant difference was found (Kruskal-Wallis chi-squared = 0.1148, p-value = 1) among the median values for all the parameters from 2018 to 2022, further analysis showed reduced median values for HB and RBC and a high median value for total WBC in 2022, with ALT and AST parameters showing a consistent declining trend from 2018 to 2022.

Conclusion: In this study, we found no seasonal effects on the reference intervals of haematological and biochemical parameters. However, changes in patterns in certain parameters, such as HB, RBC, liver enzymes, and potassium, from January to December were profoundly recognised. This suggests that seasonal variations should be considered when interpreting laboratory results and conducting future reference interval studies. Further assessment of the declining trend in AST and ALT levels from 2018 to 2022 should be conducted.

Keywords: Dry Season; Wet Season; Haematological Parameters; Biochemical Parameters

Introduction

Routine haematological and biochemical parameters play crucial roles in assessing an individual's health and diagnosing various medical conditions(1–4). Several factors, such as environmental conditions including temperature, humidity, and rainfall, could contribute to seasonal and yearly variation in both haematological and biochemical parameters. Changes in environmental conditions such as temperature, humidity, and exposure to sunlight can influence an individual's dietary preferences and physical activity levels (5–8). These lifestyle choices driven by environmental changes may affect the metabolic processes and overall health of an individual. Additionally, the prevalence of certain diseases, such as respiratory infections, especially from human coronaviruses, malaria, and parasitic infestations, often exhibits seasonal patterns which could impact haematological and biochemical parameters, especially total white cell count and differentials. Furthermore, the novel coronavirus pandemic in 2019 (COVID-19) has caused enormous lifestyle changes such as physical inactivity and less access to a healthy diet in many parts of the world which could also impact the general health of a particular population or country (9–11).

The investigation of seasonal variations and yearly trends in routine parameters in the last five years in routine parameters is of both clinical and epidemiological significance. In the context of seasonality, few studies conducted in sub-Saharan Africa considered the seasonal effects on haematological and biochemical parameters reference intervals and have shown to affect the reference intervals (1–3,12). However, an earlier study conducted in Rwanda, Uganda and Zambia reported that seasonality had minimal impact on adult clinical laboratory parameter values in these populations, suggesting that seasonal variation may not be an important factor in the evaluation of adult clinical laboratory parameters (13). Despite these contradictory findings, seasonal influences on haematological and biochemical parameters

can potentially influence the interpretation of laboratory results, the diagnosis of diseases, and the management of patient care. Amid the ongoing country-tailored reference interval study project, this retrospective study has the potential to contribute to the refinement of our geographically and genetically tailored reference ranges for routine parameters in the context of seasonality, ensuring more accurate and context-specific interpretation of laboratory results.

In this study, the presence and extent of seasonal variations in a comprehensive set of haematological and biochemical routine parameters in healthy Gambian adults and their effects on our defined reference intervals were evaluated. We further shed light on the trending pattern of main haematological and biochemical parameters in the last five years. By leveraging the retrospective data captured in the electronic medical record system of the Medical Research Council Unit The Gambia at LSHTM (MRCG@LSHTM) clinical laboratories, the description of these variations and their potential clinical implications was provided.

Methods

Study Settings

The study was conducted in the clinical laboratory department hosted at the Kuyateh Building at MRCG@LSHTM. The clinical laboratory of MRCG@LSHTM is in the Kanifing municipality, greater Banjul area, the Gambia. The Gambia is geographically situated in West Africa with a population of about 2.5 million and an area of 10,000 km2.

Clinical laboratory departments composed of clinical microbiology, clinical haematology and clinical biochemistry laboratories attained good clinical laboratory practice in 2011(14,15) and ISO15189 accreditation in 2016 (16) with laboratory and clinical data capture and management through a certified electronic medical records system. Part of the core

responsibility of the clinical laboratory department is to provide standard and quality-oriented diagnostic services to MRCG@LSHTM staff, clinical trial projects, as well as government and private hospitals. MRCG@LSHTM staff as well as the general population access routine medical examination in the clinical laboratory department and the haematological and/or biochemical results data were captured in the electronic database.

Study Population and Design

This study was a retrospective study retrieving haematological full blood count results data and biochemical data from January 2018 to December 2022 from the MRCG@LSHTM electronic medical record system database. From the database, 503 full blood counts and 772 biochemical data were retrieved after being granted access 1st January 2023. Based on our record, we focused on healthy individuals who visited the clinical laboratories department for routine medical examination or medical assessment without any underlying medical conditions. The full blood count comprised haemoglobin, RBC, platelets, WBC and differentials. Biochemical parameters tested were potassium, urea, creatinine, aspartate transaminase, alkaline transferase, glutamyl gamma transferase, total protein, albumin, and globulin were extracted.

We searched for Human Immunodeficiency Virus (HIV) and Hepatitis B serological results for these individuals and excluded HIV and Hepatitis B serological-positive individuals based on their electronic medical records. As part of the exclusion criteria, we also restricted the age range from 18 to 63 as per the employment age range in MRCG@LSHTM policies and excluded data from pregnant women based on clinical data in the electronic system.

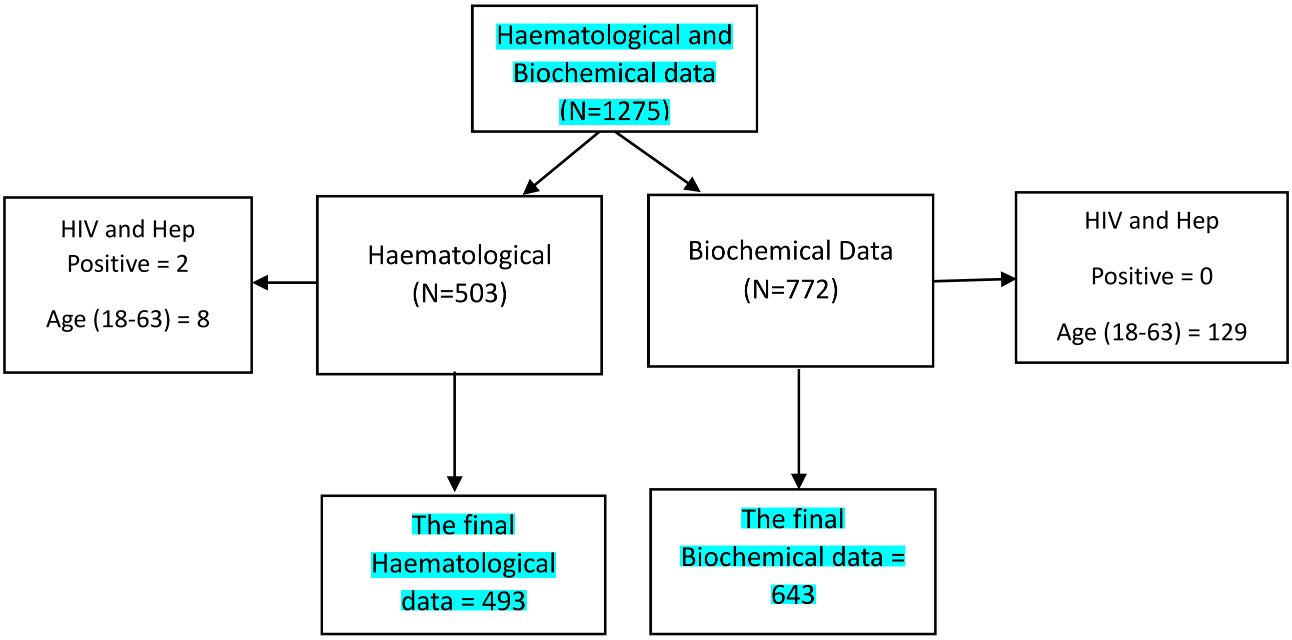

Figure 1: Flow chart demonstrating the data retrieval process

Laboratory Method and Equipment Employed

Data in the records analysed were generated from two blood samples collected per individual, one in a serum separator plain tube for serum analysis and another with an Ethylenediaminetetraacetic acid tube (EDTA) for full blood count analysis. A total of 5 mL of blood was collected per individual. The biochemistry blood samples were centrifuged within two hours of collection and serum was harvested and analysed using Ortho Clinical Diagnostics Vitros 350 Chemistry Analyzer (Ortho Clinical Diagnostics, New Jersey, United States), Roche Cobas C111 Chemistry Analyzer (Roche Diagnostic, Basel, Switzerland), or Randox Daytona Plus Bio Chemistry Analyzer (Randox Laboratories, Co. Antrim, Northern Ireland) for serum Gamma-glutamyl transpeptidase (GGT), Sodium, Potassium, Urea, Creatinine, Aspartate aminotransferase (AST), Alanine Aminotransferase (ALT), Total Protein (TP), Albumin (ALB), and Globulin. The haematological samples were placed on a Denley Spiramix 5 roller for proper mixing followed by analysis of total WBC (WBC), RBC (RBCS) Haemoglobin HGB), Platelets (PLT), Neutrophils, Lymphocytes, Monocytes, and Eosinophils within two hours of collection. For full blood count, different haematology

analysers were used over the last five years in the haematology laboratory; 5-part HumaCount 5D analyzer (Human, Germany), 5-part Cell Dyn Ruby analyzer (Abbott Diagnostics, USA) or 5-part Sysmex XN-1500 haematology analyzer (Sysmex, Germany).

Ethics approval and consent to participate

This study was approved by the Gambia Government/MRCG Joint Ethics Committee (Project ID/Ethics ref: L2023.E03). Since all data retrieved from the database were anonymised and coded, the informed consent was waived by the Ethics Committee. All methods were performed in accordance with Declaration of Helsinki.

Statistics

The data was retrieved in Microsoft Word Excel and analysed using the R package to obtain summary statistics including the mean, median and plots. Shapiro test was validated by Qqplot and a normal distribution bell-shaped curve was employed to study the distribution pattern of our data across the parameters. Boxplot was employed to assess the presence of outliers in datasets. Reference intervals were determined based on 2.5th and 97.5th percentiles after eliminating the outliers in our data using the interquartile range calculations (25th- 1.5*IQR; 75th- 1.5*IQR). The comparison between the dry and wet season median was performed by nonparametric Wilcox Rank test in R. Multivariate analysis using a generalised linear modelling approach in R was employed to model the association between explanatory variables (season, age, gender, and year) and haematological and biochemical parameters. We further compared the medians of 2018 to 2021 using the Dunn test in R to generate Kruskal-Wallis chi-squared and p-value results.

Result

From this study, four hundred and ninety-three (N= 493) haematological full blood count data comprising RBC, HB, PLT, and WBC and differentials were retrieved and analysed

(**table 1**). In the case of biochemistry data, six hundred and forty-three (643) biochemistry laboratory data comprising liver enzymes, sodium, potassium, urea and creatinine were retrieved and analysed. The age range spans 18 to 63 years for both haematological and biochemistry laboratory data. The mean and median ages of haematological data were 31 years and 28 years respectively. However, the mean (36 years) and median (33 years) age of biochemistry laboratory data were higher but not significantly different (table 1).

Over the five years from 2018 to 2022, we defined the dry and wet seasons based on the visit date (i.e. sample collection date) at the clinical laboratory department, where visits from June to October represented the wet season and November to May, the wet season. Based on the visit date, 55% of the full blood count data and 58% of the biochemistry laboratory data were generated in the dry season (**table 1**).

Unlike biochemistry laboratory data that showed almost equal gender proportion, there was gender disparity in haematological laboratory data, with 60% of the routine medical examination of full blood count data from the male population (**table 1**).

Seasonal and Yearly Effects and Reference Intervals

Despite the primary focus being to assess the seasonal effects on the biochemical and haematological parameters, we analysed the association of explanatory variables gender and age with the haematological and biochemical parameters as shown in Table 2. Using generalised linear modelling (GLM), the results showed an association between seasonality and the following haematological parameters (HB: 0.014(0.015), $P<0.05$), RBC: -0.184(0.061), $P< 0.003$; PLTs: 17.47 (6.86), $p<0.01$; WBC: 0.243(0.163), $p =0.0014$; neutrophils: 0.271(0.131), $P<0.05$; and lymphocytes: -0.003(0.066), $P= 0.01$). However, biochemical parameters were not affected by the explanatory variables, dry and wet seasons.

In the context of age, haemoglobin (0.028(0.011); $P =0.011$), platelets (-0.918(0.44): P =

0.039), creatinine (1.202(0.472); P<0.01) and total protein (-0.117(0.048=9); P<0.05) parameters were shown to have an association with age (table 2). Furthermore, several haematological parameters; bar monocytes and eosinophils were associated with gender, but only sodium (0.680(0.323); P=0.04) biochemical parameter was associated with gender. We also assessed the association of the year of visit on the parameters in healthy individuals in **Table 2**. Using the GLM approach, haematological parameters such as haemoglobin (0.156(0.079): P<0.05), RBC (0.080(0.034): P<0.05), platelets (7.861(3.16); P<0.05) and biochemical parameters such as sodium (-0.93(0.31); P<0.05), potassium (-0.107(0.041); P=0.013) albumin (-0.89(0.43); P=0.04) and globulin (1.33(0.50); P=0.01) were shown to have a significant association with year of visit in our data.

We nonparametrically defined reference intervals from our data based on 2.5th and 97.5th percentiles for haematological and biochemical parameters (**table 3**). To assess the effects of seasonality, we compared the median of the dry season to the wet season median each year, from 2018 to 2022. Even though the difference was not statistically significant across all the parameters (Wilcoxon test, P>0.05), the median for platelets as well as WBC and differentials were slightly higher in the wet season compared to the dry season. On the contrary, the median for haemoglobin and RBC was lower in the wet season. We further assessed the effects of seasonality on reference intervals derived from our study data. The changes with reference intervals from the dry season to the wet season were not significant (Wilcoxon test, P>0.05), but there was a slight reduction in the upper limit of reference intervals in the wet season for haemoglobin and RBC parameters and a slight increase in the upper limit of WBC and differentials.

Monthly and Yearly Pattern in Parameters

We further analysed patterns based on changes in the monthly and yearly median of haematological and biochemical parameters. This pattern was evident in Figure 2 as well as

(Figures S1 and S2) which demonstrated a change in the pattern of our monthly median of biochemical and haematological parameters from January to December in five years. From this pattern, the result demonstrated that the median values for haemoglobin and RBC parameters were high in the wet season but moderately low in the dry season whereas the median values for WBC and differentials including platelets were slightly high from November to March. For biochemistry parameters, liver enzymes (AST, ALT, GGT, total protein) and potassium were generally high in the wet season (June to October) but moderately low in the dry season.

Finally, we assessed how the median values of the parameters varied from 2018 to 2022 using the boxplot in **Figure 3 and Figure 4 followed by a statistical significance test with a non-parametric test, Kruskal-Wallis test.** Although no significant difference was found (Kruskal-Wallis chi-squared = 0.1148, p-value = 1) among the median values of all the parameters within the last five years (2018 to 2022), the median values for HB and RBC parameters have decreased in 2020 while total WBC count, absolute neutrophils count and platelets medians have increased, albeit slightly. For biochemical parameters, no obvious pattern was observed bar AST and ALT in which we have observed a downward trend from 2018 to 2022.

Table 1: Summary table of characteristics of sample population

**Explanatory variables**	**Characteristics**	**Summary N(%)**
**Haematology laboratory**		
Season (N=493)	Dry (n)	271 (55.0)
	Wet (n)	222 (45.0)
Gender (N=493)	Male (n)	300 (60.85)

	Female (n)	193 (39.15)
Dry Season (N=271)	Male (n)	175 (64.58)
	Female (n)	96 (35.42)
Wet Season (N=222)	Male (n)	125 (56.31)
	Female (n)	97 (43.69)
Age	Age range	18-63
	Age (Median)	28.00
	Age (Mean)	30.54
**Biochemistry Laboratory**		
Season (N=643)	Dry (n)	374(58.16)
	Wet(n)	264(41.84)
Dry Season (N=374)	Male()	180(48.13)
	Female (n)	191(51.87)
Wet Season (N=264)	Male(n)	139(52.65)
	Female(n)	122(47.35)
Gender (N= 643)	Male (n)	321 (49.92)
	Female(n)	316(50.08)
Age	Age range	18-63
	Age (Median)	33
	Age (Mean)	36.16

Table 2: Association study of explanatory variables season, age and gender, and all the haematological and biochemical parameters. The association was determined by estimated effects on the median (SE) value of the parameters. P value <0.05; Standard Error of the estimate intercept (SE)

Laboratory parameters	Season[a] Estimate(Std. Error); P value <0.05	Age (Estimate(Std. Error); P value <0.05	Gender[b] (Estimate(Std. Error); P value <0.05	Year (Estimate(Std. Error); P value <0.05
HB (g/dL)	**0.014(0.015); p<0.05**	**0.028(0.011); P =0.011**	**2.355(0.126); P<0.0001**	**0.156(0.079): P<0.05**
RBCa(106/uL)	**-0.184(0.061); p<0.05**	0.004(0.003); P=0.287	**0.750(0.049); P<0.0001**	**0.080(0.034): P<0.05**
PLT (103/uL)	**17.47 (6.86); p<0.01**	**-0.918(0.44): P = 0.039**	**-60.19(6.276); P<0.0001**	**7.861(3.16); P<0.05**
WBC (103/uL)	**0.243(0.163); P<0.01**	-0.006(0.011); P = 0.566	**-0.929(0.158); P<0.0001**	0.030(0.071) P>0.67
Neutrophils (103/uL)	**0.271(0.131); P<0.05**	-0.009(0.008); P=0.295	**-0.624(0.129); P<0.0001**	-0.027(0.05); P=0.61
Lymphocytes (103/uL)	**-0.003(0.066);P< 0.01**	0.006(0.004); P= 0.169	**-0.288(0.0654); P<0.0001**	0.038(0.03); P= 0.22

Monocytes (103/uL)	-0.004(0.165); P=0.798	-0.002(0.001); P=0.092	-0.027(0.017); P = 0.11	**0.037(0.007); P<0.05**
Eosinophils (103/uL)	-0.021(0.023); P=0.359	-0.001(0.001); P=0.380)	0.011(0.023); P= 0.623	-0.01(0.01); P=0.139
Sodium(mmol/L)	-0.238(0.330);P =0.471	0.0188(0.013); P=0.156	**0.680(0.323); P=0.04**	**-0.93(0.31); P<0.05**
GGT (U/L)	0.213(7.31); P=0.977	0.227(0.241); P= 0.347	10.696(7.27); P=0.142	-0.43(1.39); P=0.76
Potassium (mmol/L)	0.020(0.039);P=0.6143	0.001(0.001): P=0.424	0.065(0.039); P=0.098	**-0.11(0.04); P=0.013**
Urea (mmol/L)	-4.128(0.259);P=0.112	0.064(0.040); P=0.113	1.225(1.301); P=0.347	-0.11(0.136); P= 0.42
Creatinine (umol/L)	-18.13(14.88);P=0.224	**1.202(0.472); P<0.01**	28.15(15.23); P=0.066	0.62(2.00); P=0.76
AST (U/L)	-2.045(2.256);P<0.344	-0.066(0.069); P= 0.597	0.566(0.256); P=0.798	-0.56(0.65); P= 0.40
ALT (U/L)	-1.619(2.60); P=0.534	0.031(0.089); P=0.729	1.244(2.846); P=0.662	-1.69(1.12); P=0.14
Albumin (g/dL)	-1.663(0.863); P= 0.06	-0.052(0.029); P=0.08	2.028(0.814); P=0.07	**-0.89(0.43); P=0.04**
Total Protein (g/dL)	-2.142(1.465); P=0.148	**-0.117(0.048=9); P<0.05**	1.017(1.408); P=0.472	-0.51(0.64); P=0.43
AST (U/L)	-0.536(1.228); P=0.663	-0.064(0.041); P= 0.12	-1.057(1.168); 0.367	**1.33(0.50); P=0.01**

[a]Wet Season(reference) is compared to Dry Season; [c]Male (reference) is compared to Female gender;

Table 3: The effect of exploratory seasonal factor on the reference ranges of the haematological and biochemical parameters (Wilcoxon test P>0.05).

**Laboratory parameters**	**N**	**Median (Reference Range)**	**N**	**Median (Dry Season)**	**N**	**Wet Season**
HB (g/dL)	461	13.90(11.0-16.5)	258	14.0(11.34-16.36)	206	13.70(10.70-16.29)
RBC[a](10^6/uL)	444	4.87(3.90-5.85)	238	4.95(4.06-5.89)	201	4.77(3.77-5.76)
PLT (10^3/uL)	433	244.5(158.6-346.2)	238	237(156.9-338.2)	197	248(163.5-361.1)
WBC (10^3/uL)	437	5.40(3.52-7.65)	245	5.34(3.51-7.60)	194	5.34(3.52-7.80)
Neutrophils (10^3/uL)	426	2.55(1.34-4.05)	235	2.47(1.32-3.81)	194	2.49(1.40-4.55)
Lymphocytes (10^3/uL)	418	2.15(1.33-2.96)	232	2.14(1.30-3.065)	192	2.14(1.33-2.93)
Monocytes (10^3/uL)	416	0.43(0.25-0.64)	230	0.42(0.25-0.62)	190	0.43(0.25-0.64)
Eosinophils (10^3/uL)	335	0.13(0.13-0.31)	201	0.14(0.02-0.36)	138	0.11(0.01-0.26)
Basophils (10^3/uL)	350	0.04(0.01-0.07)	185	0.03(0.02-0.06)	146	0.04(0.02-0.07)
Sodium (mmol/L)	384	140(136-144)	215	140(137-144)		140(136-144)

Potassium (mmol/L)	498	4.12(3.6-4.7)	235	4.20(3.6-4.7)	152	4.10(3.70-4.50)
GGT (U/L)	240	26(11.00-52.025)	134	26(10.23-51.03)	106	26(12.78-53.00)
Creatinine (umol/L)	500	75.2(50.48-107.05)	289	75(49.00-107.08)	211	77(52.93-106.68)
Urea (mmol/L)	507	3.1(1.7-4.9)	381	3.1(1.80-4.7)	217	3.1(1.70-4.9)
AST (U/L)	354	20(12-32)	211	20(12-34.5)	138	20(12.43-29.00)
ALT (U/L)	234	17(7.0-33.0)	130	17(6.23-33.78)	104	17(8.15-31.43)
Albumin (g/dL)	236	40(34-47)	150	40(34.00-47.00)	82	40(34.03-45.00)
Total Protein (g/dL)	336	76(67.00-83.63)	195	76(68.00-85.00)	147	76(67.00-83.35)
Globulin (g/dL)	203	35(27.0-42.00)	116	35(27-42.13)	73	35(29.8-40.0)

[a]Mean value was used for RBC parameter because the data was normally distributed

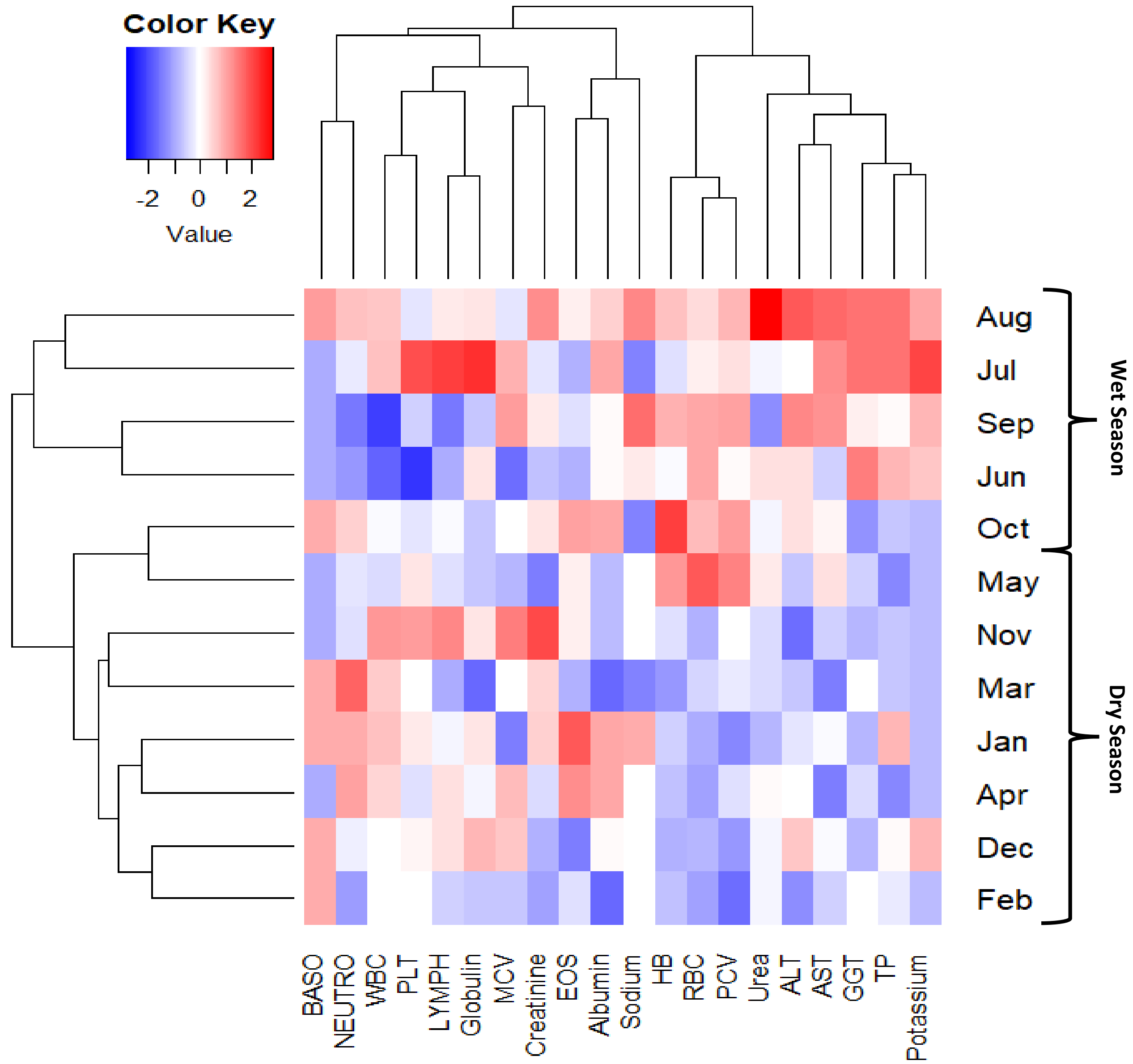

Figure 2: Monthly pattern of haematological and biochemical parameters from 1st January 2018 to 31st December 2022 in five year period. Haemoglobin (HB); Red blood cells (RBC); White blood cells (WBC); Platelets (PLT); Neutrophils (NEUTRO); Lymphocytes (LYMPH); Mean cell volume (MCV); Packed cell volume (PCV); Eosinophils (EOS); Basophil (BASO); Aspartate aminotransferase (AST); Alanine transaminase (ALT); Gamma-glutamyl transferase (GGT).

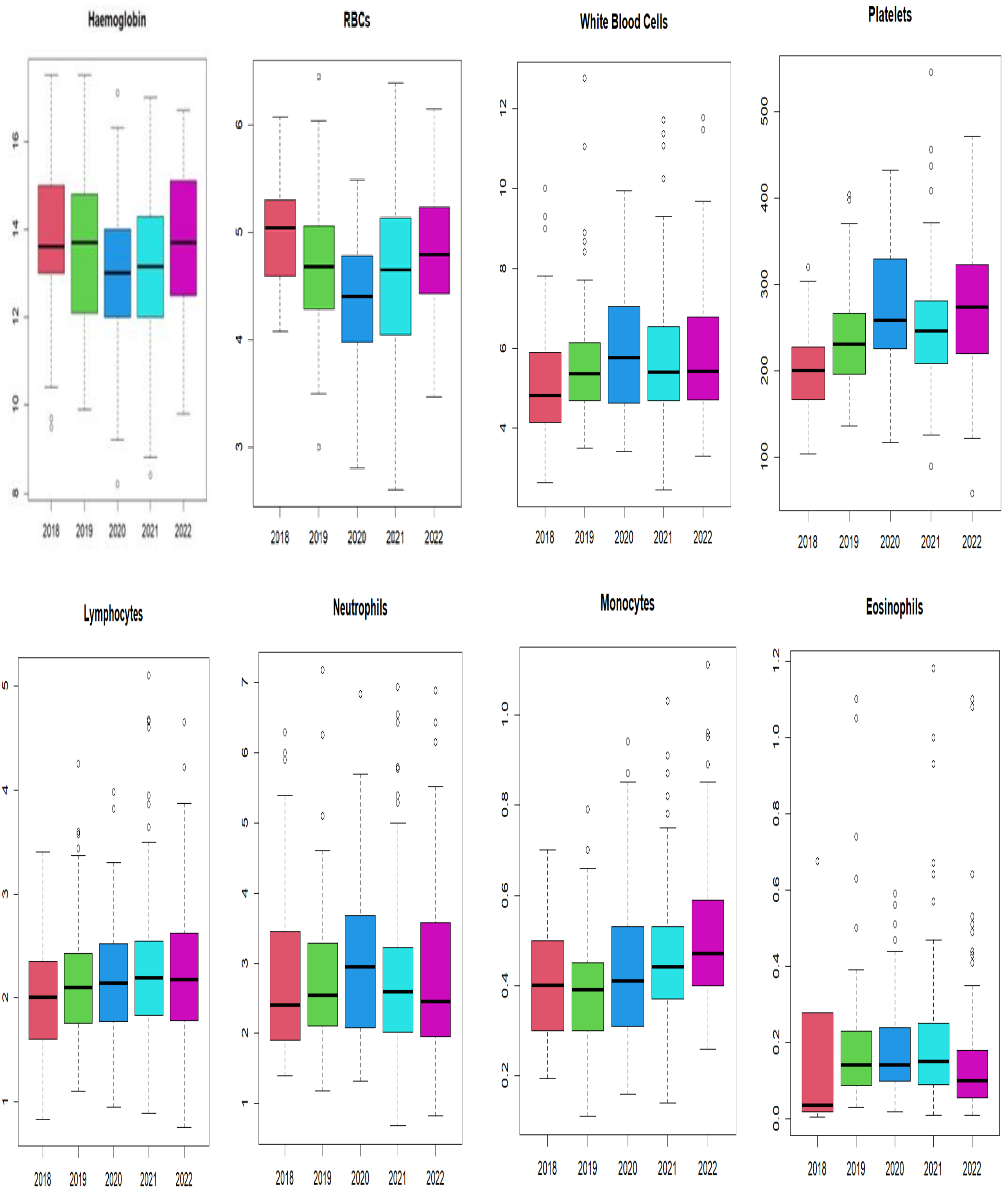

Figure 3: Boxplot showing pattern/trend of haematological parameters in the last five years. The results displayed in medians (Kruskal-Wallis chi-squared = 0.063, p-value = 1).

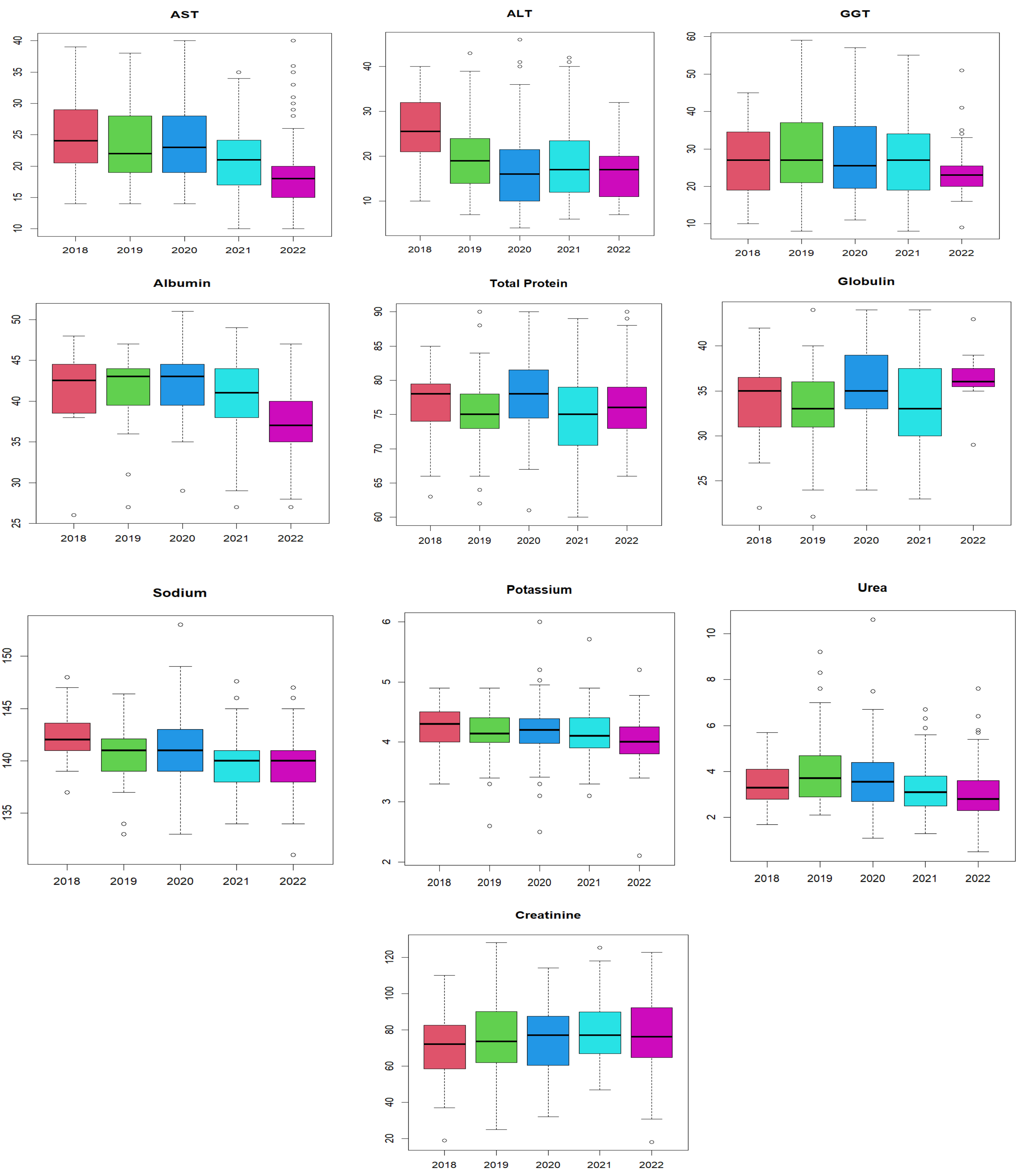

Figure 4: Boxplot showing pattern/trend of biochemical parameters in the last five years. Aspartate aminotransferase (AST); Alanine transaminase (ALT); Gamma-glutamyl transferase (GGT). (Kruskal-Wallis chi-squared = 0.8433, p-value = 0.93)

Discussion

The study evaluated seasonal and yearly effects on haematological and biochemical parameters and on the derived reference intervals as seasonal variations could be influenced by factors such as changes in environmental conditions, diet, physical activity and certain seasonal diseases like malaria. Overall, the differences between the dry and wet seasons were not statistically significant and hence no seasonal effect was recognised in reference intervals derived from our study data. These findings were in contrast to what was reported by Okebe et al 2016 prospective study conducted in the Gambia children below five years, which could be attributed to the difference in the study groups. However, the reference intervals derived from our study data were comparable to the study of Adetifa et al. 2009 which targeted healthy Gambian Adults during the TB contact tracing study. The neutrophils reference intervals upper-limit difference between the dry season (1.32-3.81) and wet season (1.40-4.55) indicate that perhaps there is a need to generate a more informative reference which contextualises seasonality. These seasonal variations are important to put into consideration, particularly in research studies or clinical trials in which patients are monitored at different times of the year. Awareness of these subtle seasonal variations may deter erroneous conclusions from the study data.

To decipher the monthly pattern in the parameters from January to December, we discovered high HB and RBC medians in the wet season. Haemoglobin and RBC parameters are integral in diagnosing anaemia in clinical settings(20). It has been reported that healthy individuals may experience variations in haemoglobin levels due to factors like changes in diet such as increased consumption of iron-rich foods in certain seasons, and asymptomatic infection (22). During the wet season, the nutritional pattern in the Gambia leans toward the abundant availability of iron-rich foods which could elucidate the possible high haemoglobin and RBCs in the wet season in the Gambia (21).

The seasonal variations were observed in our study in WBC and differential as well as PLT. In the Gambia, environmental changes such as increased rainfall in the wet season together with cold weather that spans from November to March favour transmission of malaria parasites which peaks around November, and respiratory infections (24–29). Our study of seasonal effects on white blood cells and differentials is supported by early findings (1). Many infectious diseases such as malaria flourish in the wet season, likely due to environmental influences such as rainfall causing massive water stagnation and numerous waterlogged areas (30-33). This could potentially lead to an increase in mean WBC count and differentials in the general population due to systemic low-grade inflammation in the community as a consequence of malaria transmission and viral illnesses (3,10).

Like HB and RBCs, the monthly pattern of liver enzymes and potassium was changed based on seasonality. Our finding is concordant with early studies which have shown that seasonal-induced changes in diet and fluid intake can influence biochemical parameters, particularly electrolytes. For the biochemical parameters, the temperature difference between the wet and dry seasons could explain the seasonal effects (34–36). A reference interval study conducted in Guinea also reported seasonal effects of biochemical parameters, particularly liver enzymes (31). Our urea and creatinine median values were relatively normal across the dry and wet seasons despite reports that have shown that dehydration can be more common in the dry season and could affect parameters like serum creatinine and urea levels (37).

Although we have observed no significant difference in median values of all the parameters in the last five years, the reduction in median values for HB and RBC but the increase in total WBC median values in 2020 could be attributed to the slight effects of the coronavirus disease 2019 (COVID-19) pandemic. The pandemic impacted lifestyles including limited access to healthy diet and fresh fruits and vegetables, changes in eating patterns, as well as physical inactivity (39). In the Gambia, the first case of the COVID-19 pandemic was

identified on 17 March 2020 which eventually culminated in a government-enforced lockdown and restrictions amid rising cases of Covid-19 due to curtail community transmission(40). Alarmingly, our study result showed a consistent decline in the median of AST and ALT biochemical parameters, warranting further investigation. Our public health authorities need to be aware of the trend of haematological and biochemical parameters to assess the lasting impact of the pandemic on the public health level.

As seen in most respective studies, our study has limitations constraining the control of certain factors such as haemoglobin genotype status, body size, smoking, alcohol, exercise and contraceptive pills that could influence the outcome (17). The sample size for example was also constrained by available data with no options for additional data to improve statistical power (18,19). These limitations could be addressed by a well-designed prospective study in a healthy population congruent to the approach employed by Okebe et al. 2016. Despite these limitations, we believe that the results of this study are accurate, as the sample size here was significantly considerable. Therefore, our study was still representative, supporting the current perspective on reference intervals that will provide a more comprehensive sampling and data stratification reflecting dry and wet seasons.

Conclusion

Overall, our study found no seasonal effects on haematological and biochemical parameters' reference intervals despite some parameters showing an association with seasonality. However, variations in certain parameters such as HB, RBC, liver enzymes as well and potassium from January to December were demonstrated. Furthermore, the yearly variations in the median values for all the parameters were not significant despite the COVID-19 pandemic in 2020. Hence, our findings suggest that seasonal variations in haematological and biochemical parameters should be considered when interpreting laboratory results and making better diagnostic decisions and participant recruitment in clinical trial projects. This

study should be followed by well-designed context-specific studies that will employ standardised sampling and analytical methods in our geographical setting.

Acknowledgements

Not Applicable

Availability of Data and Materials

The datasets used and/or analysed during the current study are available from the corresponding author on reasonable request.

Funding

This work was supported by the Medical Research Council Unit The Gambia at the London School of Hygiene and Tropical Medicine.

Consent for publication

Not applicable

Competing Interest

I declare that the authors have no competing interests as defined by BMC, or other interests that might be perceived to influence the results and/or discussion reported in this paper.

Abbreviations

COVID-19: Coronavirus Disease 2019

LSHTM: London School of Hygiene and Tropical Medicine

MRCG@LSHTM: Medical Research Council Unit The Gambia at LSHTM

HIV: Human Immunodeficiency Virus

GGT: Gamma-glutamyl transpeptidase

AST: Aspartate aminotransferase

ALT: Alanine Aminotransferase

TP: Total Protein

ALB: Albumin

WBC: White Blood Cells

RBC: Red Blood Cells

HB: Haemoglobin,

PLT: Platelets

GLM: Generalised Linear Modelling

Author's Contributions

MD and AAN designed the study and drafted the manuscript. MD, MDD, FS, RB, NS, MB, and SU performed the laboratory analysis, data retrieval and performed data arrangements. MD and AAN performed the statistical analysis. All authors reviewed and approved the final manuscript.

References

1. Bæk O, Jensen KJ, Andersen A, Balé C, Martins C, Biering-Sørensen S, et al. Seasonal and sex-specific variations in haematological parameters in 4 to 5.5-month-old infants in Guinea-Bissau, West Africa. Trans R Soc Trop Med Hyg. 2017;111(1).

2. Béavogui AH, Touré AA, Camara D, Doumbouya A, Sylla MM, Touré S, et al. Haematological and Biochemical Reference Values for Healthy Population of Maferinyah Rural Community in Guinea. Biomed Res Int. 2020;2020.

3. Okebe J, Mwesigwa J, Agbla SC, Sanya-Isijola F, Abubakar I, D'Alessandro U, et al. Seasonal variation in haematological and biochemical reference values for healthy young children in The Gambia. BMC Pediatr. 2016;16(1).

4. Gidlow DA, Church JF, Clayton BE. Seasonal Variations in Haematological and Biochemical Parameters. Ann Clin Biochem. 1986;23(3).

5. Marriott BM. Nutritional Needs in Hot Environments: Applications for military personnel in Field Operations. Nutritional Needs in Hot Environments. 1993.

6. Chan CB, Ryan DA. Assessing the effects of weather conditions on physical activity participation using objective measures. Vol. 6, International Journal of Environmental Research and Public Health. 2009.

7. Pasiakos SM. Nutritional Requirements for Sustaining Health and Performance during Exposure to Extreme Environments. Vol. 40, Annual Review of Nutrition. 2020.

8. Turrisi TB, Bittel KM, West AB, Hojjatinia S, Hojjatinia S, Mama SK, et al. Seasons, weather, and device-measured movement behaviors: a scoping review from 2006 to 2020. Vol. 18, International Journal of Behavioral Nutrition and Physical Activity. 2021.

9. Akinbo FO, Okaka CE, Omoregie R. Seasonal Variation of Intestinal Parasitic Infections Among HIV-Positive Patients in Benin City, Nigeria. Ethiopian journal of health sciences. 2011;21(3).

10. Mwesigwa J, Okebe J, Affara M, Di Tanna GL, Nwakanma D, Janha O, et al. On-going malaria transmission in The Gambia despite high coverage of control interventions: A nationwide cross-sectional survey. Malaria Journal. 2015;14(1).

11. Collins KA, Ceesay S, Drammeh S, Jaiteh FK, Guery MA, Lanke K, et al. A Cohort Study on the Duration of Plasmodium falciparum Infections During the Dry Season in The Gambia. Journal of Infectious Diseases. 2022;226(1).

12. Serena V, Alessandro M, Maurizio, Nadia T, Valeria S, Eleonora C, et al. Baseline haematological and biochemical reference values for healthy male adults from mali. Pan African Medical Journal. 2019;32.

13. Ruzagira E, Abaasa A, Karita E, Mulenga J, Kilembe W, Allen S, et al. Effect of seasonal variation on adult clinical laboratory parameters in Rwanda, Zambia, and Uganda: Implications for HIV biomedical prevention trials. PLoS ONE. 2014;9(8).

14. Ezzelle J, Rodriguez-Chavez IR, Darden JM, Stirewalt M, Kunwar N, Hitchcock R, et al. Guidelines on good clinical laboratory practice: Bridging operations between research and clinical research laboratories. Vol. 46, Journal of Pharmaceutical and Biomedical Analysis. 2008.

15. Joshi RK, Sarangi SC, Mohapatra S, Mallick S, Datta SK. A Comparative Review of ICMR, WHO, and EMA Guidelines for Good Clinical Laboratory Practices. Journal of Laboratory Physicians. 2023;15(02).

16. International Organization for standardization. ISO 15189:2012 - Medical laboratories-Requirements for quality and competence. International Organization for standardization ISO. 2012;2012.

17. Talari K, Goyal M. Retrospective studies - Utility and caveats. Journal of the Royal College of Physicians of Edinburgh. 2020;50(4).

18. Suresh K, Chandrashekara S. Sample size estimation and power analysis for clinical research studies. Vol. 5, Journal of Human Reproductive Sciences. 2012.

19. Kim J, Seo BS. How to calculate sample size and why. Clinics in Orthopedic Surgery. 2013;5(3).

20. Critchley J, Bates I. Haemoglobin colour scale for anaemia diagnosis where there is no laboratory: A systematic review. International Journal of Epidemiology. 2005;34(6).

21. World Health Organization. Countdown to 2015 decade report (2000-2010) with country profiles: taking stock of maternal, newborn and child survival. Maternal, Newborn & Child Survival Countdown to 2015. 2015;

22. Semagn BE, Gebreegziabher ZA, Abebaw WA. Iron-rich food consumption and associated factors among children aged 6–23 months in Sierra Leone: multi-level logistic regression analysis. BMC Public Health. 2023;23(1).

23. Ayanful-Torgby R, Quashie NB, Boampong JN, Williamson KC, Amoah LE. Seasonal variations in Plasmodium falciparum parasite prevalence assessed by varying diagnostic tests in asymptomatic children in southern Ghana. PLoS ONE. 2018;13(6).

24. Chowdhury FR, Ibrahim QSU, Shafiqul Bari M, Jahangir Alam MM, Dunachie SJ, Rodriguez-Morales AJ, et al. The association between temperature, rainfall and humidity with common climate-sensitive infectious diseases in Bangladesh. PLoS ONE. 2018;13(6).

25. Prentice AM, Mendoza YA, Pereira D, Cerami C, Wegmuller R, Constable A, et al. Dietary strategies for improving iron status: Balancing safety and efficacy. Nutrition Reviews. 2017;75(1).

26. Maes M, Stevens W, Scharpe S, Bosmans E, De Meyer F, D'Hondt P, et al. Seasonal variation in peripheral blood leukocyte subsets and in serum interleukin-6, and soluble interleukin-2 and-6 receptor concentrations in normal volunteers. Experientia. 1994;50(9).

27. Ruul A, Saar B. Anaemia of chronic disease. Eesti Arst. 2015;94(9).

28. Kurkina N V., Gorshenina EI, Chegodaeva L V., Polagimova A V. Anemia of Chronic Diseases. Klinicheskaya Onkogematologiya/Clinical Oncohematology. 2021;14(3).

29. Fraenkel PG. Understanding anemia of chronic disease. Hematology (United States). 2015;2015(1).

30. Fares A. Factors influencing the seasonal patterns of infectious diseases. International Journal of Preventive Medicine. 2013;4(2).

31. Abdoul Habib Béavogui, Almamy Amara Touré, Daouda Camara, Abdoulaye Doumbouya, Malick Minkael Sylla, Sékou Touré, Ahmadou Hamidou Togo, Mamadou Saliou Diallo, Alexandre Delamou, Issaka Sagara, Alassane Dicko, Abdoulaye Djimdé, "Haematological and Biochemical Reference Values for Healthy Population of Maferinyah Rural Community in Guinea", BioMed Research International. 2020;2020. https://doi.org/10.1155/2020/8605485

32. Mandala W, Munyenyembe A, Sulani I, Soko M, Mallewa J, Hiestand J. Acute Malaria in Malawian Children and Adults is Characterized by Thrombocytopenia That Normalizes in Convalescence. Journal of Blood Medicine. 2022;13.

33. Bhatia I, Sharma A, Guleria S, Sharma A, Jaswal RS, Vaid A, et al. Thrombocytopenia in Children: A Large Prospective Study on Clinical Manifestations, Seasonal Variation, Etiology, and Outcome. Journal of Association of Physicians of India. 2023;71(3).

34. Kailakov AM. Seasonal fluctuations in the content of some electrolytes in patients with diabetes mellitus. Problemy Endokrinologii. 1979;25(2).

35. Swingler G, Power D. Seasonal plasma electrolyte fluctuations in childhood diarrhoea. Archives of Disease in Childhood. 2002;87(5).

36. Koyama T, Makinouchi R, Machida S, Matsui K, Shibagaki Y, Imai N. Seasonal Changes in the Prevalence of Hyperkalemia in the Emergency Department: A Single Center Study. Medicina (Lithuania). 2022;58(2).

37. Ephraim RKD, Asamoah CA, Abaka-Yawson A, Kwadzokpui PK, Adusei S. Climate change causes changes in biochemical markers of kidney disease. BMC Nephrology. 2020;21(1).

38. Adetifa IM, Hill PC, Jeffries DJ, Jackson-Sillah D, Ibanga HB, Bah G, Donkor S, Corrah T, Adegbola RA. Haematological values from a Gambian cohort--possible reference range for a West African population. Int J Lab Hematol. 2009 Dec;31(6):615-22. doi: 10.1111/j.1751-553X.2008.01087.x. Epub 2008 Jul 10. PMID: 18631172.

39. van der Werf, E.T., Busch, M., Jong, M.C. et al. Lifestyle changes during the first wave of the COVID-19 pandemic: a cross-sectional survey in the Netherlands. BMC Public Health 21, 1226 (2021). https://doi.org/10.1186/s12889-021-11264-z

40. Abatan B, Agboghoroma O, Akemoke F, Antonio M, Awokola B, Bittaye M, Bojang A, Bojang K, Brotherton H, Cerami C, Clarke E, D'Alessandro U, de Silva T, Drammeh M, Forrest K, Hofmann N, Jagne S, Jah H, Jarju S, Jaye A, Jobe M, Kampmann B, Manjang B, Martinez-Alvarez M, Mohammed N, Nadjm B, Ndiath MO, Nkereuwem E, Nwakanma D, Oko F, Okoh E, Okomo U, Olatunji Y, Oriero E, Prentice AM, Roberts C, Roca A, Sabally B, Sambou S, Samateh A, Secka O, Sesay AK, Singhateh Y, Susso B, Usuf E, Vilane A, Wariri O. Intense and Mild First Epidemic Wave of Coronavirus Disease, The Gambia. Emerg Infect Dis. 2021; 27(8):2064-2072. doi: 10.3201/eid2708.204954.

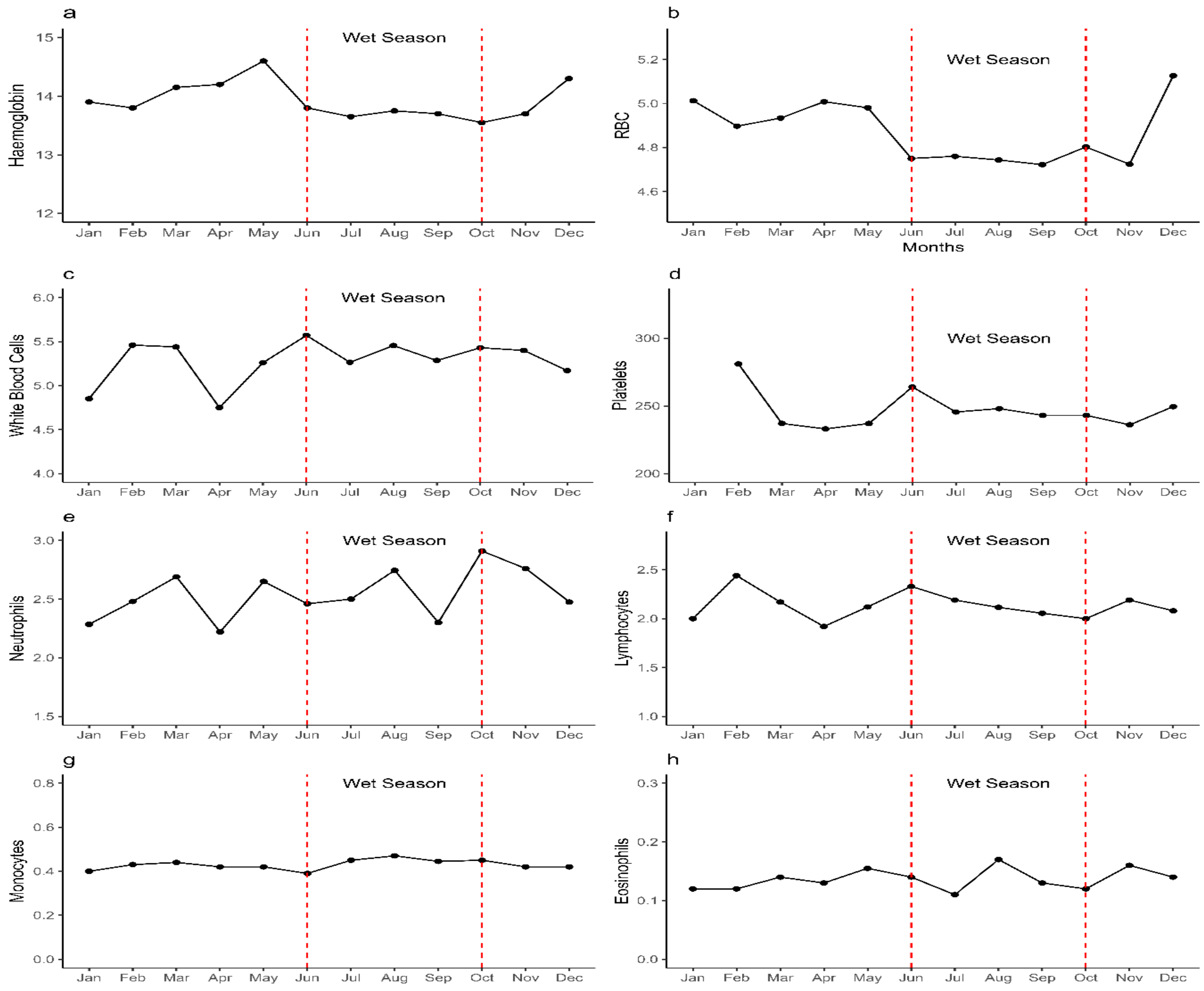

Figure S1: Haematological parameters from January to December in five year period. The point showed monthly medians in five year period. RBC (RBC)

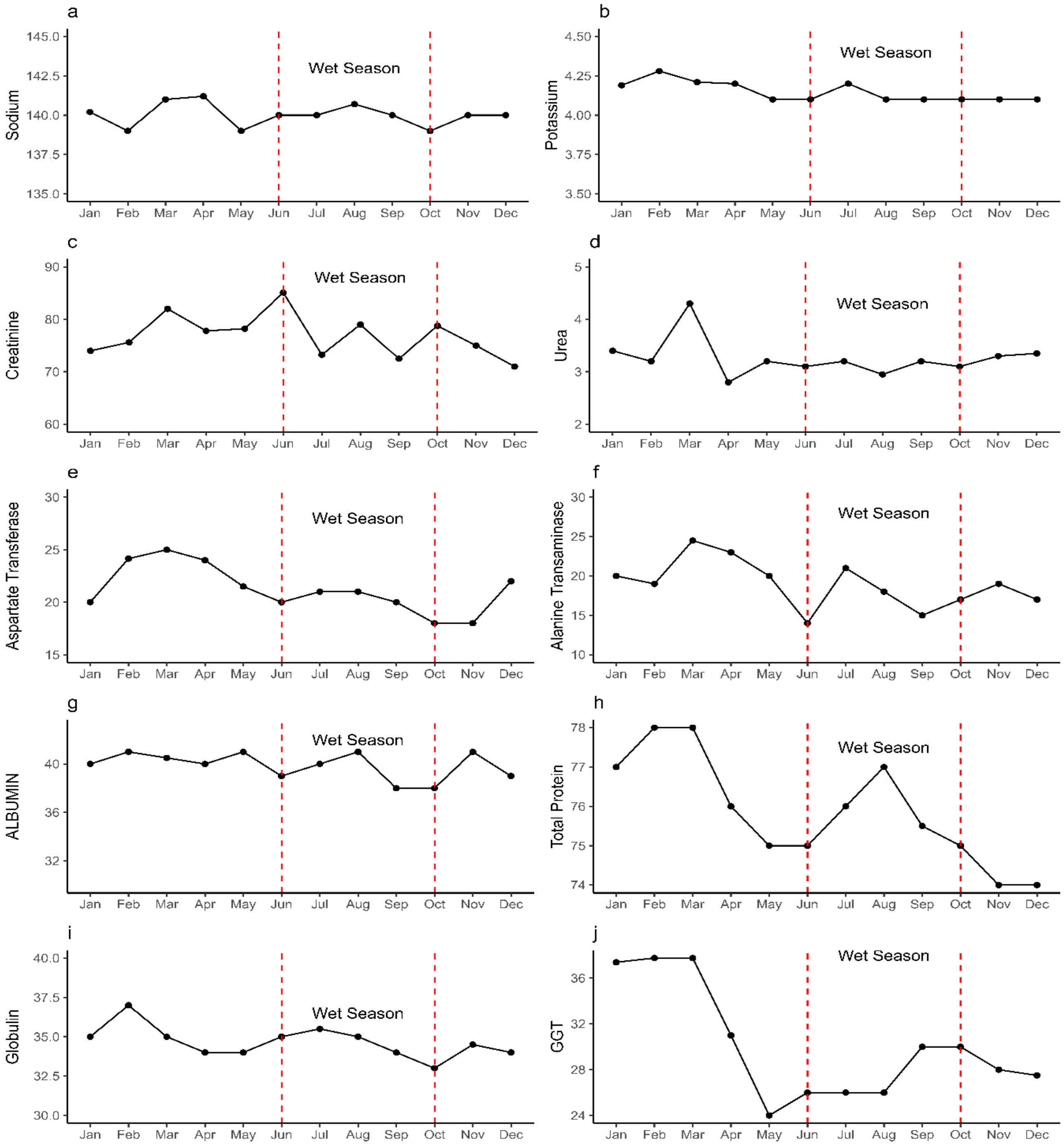

Figure S2: Biochemical parameters from January to December in five year period. The point showed monthly medians in five year period. Aspartate aminotransferase (AST); Alanine transaminase (ALT); Gamma-glutamyl transferase (GGT)

Click here to access/download
Supporting Information
Study Raw Data.xlsx
