# Supplementary material for: Seasonal variations in haematological and biochemical parameters of healthy Gambian adults: Retrospective study 2018–2022
Source: PLOS Glob Public Health. 2024 Sep 17;4(9):e0003715. doi: 10.1371/journal.pgph.0003715 (PMC11407651; doi:10.1371/journal.pgph.0003715)
Supplement: Attachment — Submitted filename: PGPH-D-23-02498_R2.pdf [file pgph.0003715.s010.pdf]

PLOS Global Public Health

Seasonal variations in haematological and biochemical parameters of healthy Gambian adults: Retrospective Study 2018-2022

--Manuscript Draft--

**Manuscript Number:**	PGPH-D-23-02498R2
**Article Type:**	Research Article
**Full Title:**	Seasonal variations in haematological and biochemical parameters of healthy Gambian adults: Retrospective Study 2018-2022
**Short Title:**	Seasonal and yearly variations in the routine haematological and biochemical parameters
**Corresponding Author:**	Mustapha Dibbasey, BSc (honours); MSc<br>University of Ghana<br>Accra, GHANA
**Order of Authors:**	Mustapha Dibbasey, BSc (honours); MSc
	Mamudou Dahaba
	Francess Sarfo
	Rosyna Begum
	Mustapha Kanteh
	Nyima Sumareh
	Mustapha Bakare
	Solomon Umukoro
	Alfred Amambua-Ngwa
**Keywords:**	Keywords: Dry Season; Wet Season; Haematological Indices; Biochemical Measurements; Seasonal Variation; Seasonality
**Abstract:**	Objective: The objectives of this study are to determine the presence and effect of seasonal variations and provide insights into trend from 2018 to 2022 in a comprehensive set of routine haematological indices and biochemical measurements in Gambian adults with no known underlying health condition.Methods: The study was conducted in the clinical diagnostic laboratory at MRCG@LSHTM. We retrieved five years of data (1st January 2018 to 31st December 2022) from an electronic database and analysed 493 full blood counts and 643 biochemical data. In this study, we focused on data from individuals with no known underlying health condition who visited the clinical diagnostic laboratory for routine medical examinations or assessments.Results: Our study found a positive association between seasonality (wet season as the reference) and HB (HB: 0.014(0.015), P<0.05), WBC (WBC: 0.243(0.163), p =0.0014), and neutrophils (neutrophils: 0.271(0.131), P<0.05) with exception to RBC (RBC: - 0.184(0.061), P< 0.003) that showed negative association. Despite the association, the seasonal effects on our derived reference intervals for haematological indices and biochemical measurements from the wet season to the dry season were not statistically significant (Wilcoxon test, P>0.05). In addition, we observed in our heatmap result that some laboratory parameters, including HB, RBC, HCT/PCV, urea, liver enzymes, and potassium, showed seasonal variation patterns throughout the year, with median levels being normal to slightly low during the dry season and normal to high during the wet season. Although no significant difference (Kruskal-Wallis chi-squared = 0.1148, p-value = 1) was found among the median values for all parameters from 2018 to 2022, further analysis showed reduced median values for HB and RBC and a high median value for total WBC in 2022. Additionally, ALT and AST parameters showed a consistent declining trend from 2018 to 2022.Conclusion: Our study found no seasonal effects on the derived reference intervals of haematological indices and biochemical measurements. However, we observed changes in patterns for certain parameters particularly HB, RBC, liver enzymes, and potassium based on seasonality .

Powered by Editorial Manager® and ProduXion Manager® from Aries Systems Corporation

Additional Information:	
Question	Response
**Financial Disclosure**<br><br>Enter a financial disclosure statement that describes the sources of funding for the work included in this submission and the role the funder(s) played. This includes grants and any commercial funding of the work or authors.<br><br>This statement will be typeset if the manuscript is accepted for publication.<br><br>*Please review the submission guidelines and the instructions link below for detailed requirements and guidance.*	This project received no specific funding for this work.
**Competing Interests**<br><br>On behalf of all authors, disclose any competing interests that could be perceived to bias this work.<br><br>This statement will be typeset if the manuscript is accepted for publication.<br><br>*Please review the instructions link below and PLOS Global Public Health's competing interests policy to determine what information must be disclosed at submission.*	The authors have declared that no competing interests exist.
**Data Availability**<br><br>Before publication, Authors are required to make fully available and without restriction all data underlying their findings. Please see our PLOS Data Policy page for detailed information on this policy.<br><br>A **Data Availability Statement**, detailing	The data for the study is fully available and submitted with manuscript.

where the data can be accessed, is required at first submission. Insert your Data Availability Statement in the box below.<br><br>Please see the data reporting section of our submission guidelines for instructions on what you need to include in your Data Availability Statement.<br><br>This statement will be typeset if the manuscript is accepted for publication.<br><br>*PLOS allows rare exemptions to address legal and ethical concerns. If you have legal or ethical restrictions, please use the box below to detail these in full sentences for the Journal team to consider.*	

Powered by Editorial Manager® and ProduXion Manager® from Aries Systems Corporation

Manuscript

Click here to access/download;Manuscript;Revised Manuscript without Track Changes v2.docx

Seasonal variations in haematological and biochemical parameters of healthy Gambian adults: Retrospective Study 2018-2022

Mustapha Dibbasey*[1,2], Mamudou Dahaba[1], Francess Sarfo[1], Rosyna Begum[1], Mustapha Kanteh[1], Nyima Sumareh[1], Mustapha Bakare[1], Solomon Umukoro[1], **Alfred Amambua-Ngwa**[1,3]

[1]Medical Research Council the Gambia at London School of Hygiene and Tropical Medicine

[2]School of Biological Science, Department of Biochemistry, Cell and Molecular Biology, University of Ghana, Ghana, Accra, Legon

[3]London School of Hygiene and Tropical Medicine

*Corresponding Author: Mustapha Dibbasey (lshmd4@lshtm.ac.uk; mdibbasey@mrc.gm)

Abstract

Objective: The objectives of this study are to determine the presence and effect of seasonal variations and provide insights into trend from 2018 to 2022 in a comprehensive set of routine haematological indices and biochemical measurements in Gambian adults with no known underlying health condition..

Methods: The study was conducted in the clinical diagnostic laboratory at MRCG@LSHTM. We retrieved five years of data (1st January 2018 to 31st December 2022) from an electronic database and analysed 493 full blood counts and 643biochemical data. In this study, we focused on data from individuals with no known underlying health condition who visited the clinical diagnostic laboratory for routine medical examinations or assessments.

Results: Our study found a positive association between seasonality (wet season as the reference) and HB (HB: 0.014(0.015), P<0.05), WBC (WBC: 0.243(0.163), p =0.0014), and neutrophils (neutrophils: 0.271(0.131), P<0.05) with exception to RBC (RBC: - 0.184(0.061), P< 0.003) that showed negative association. Despite the association, the seasonal effects on our derived reference intervals for haematological indices and biochemical measurements from the wet season to the dry season were not statistically significant (Wilcoxon test, P>0.05). In addition, we observed in our heatmap result that some laboratory parameters, including HB, RBC, HCT/PCV, urea, liver enzymes, and potassium, showed seasonal variation patterns throughout the year, with median levels being normal to slightly low during the dry season and normal to high during the wet season. Although no significant difference (Kruskal-Wallis chi-squared = 0.1148, p-value = 1) was found among the median values for all parameters from 2018 to 2022, further analysis showed reduced median values for HB and RBC and a high median value for total WBC in 2022. Additionally, ALT and AST parameters showed a consistent declining trend from 2018 to 2022.
Conclusion: Our study found no seasonal effects on the derived reference intervals of haematological indices and biochemical measurements. However, we observed changes in patterns for certain parameters particularly HB, RBC, liver enzymes, and potassium based on seasonality .

Keywords: Dry Season; Wet Season; Haematological Indices; Biochemical Measurements;

Seasonal Variation; Seasonality

Introduction

Routine haematological and biochemical parameters are essential for assessing an individual's health and diagnosing various medical conditions(1–4). Seasonal and yearly variations in these parameters can be influenced by environmental factors such as temperature, humidity, and rainfall. Changes in environmental conditions can impact an individual's dietary preferences and physical activity levels (5–8), which in turn affect their metabolic processes and overall health. Additionally, certain diseases, including respiratory infections, malaria, and parasitic infestations, often exhibit seasonal patterns that can impact haematological and biochemical parameters, particularly total white cell count and differentials. Moreover, the COVID-19 pandemic has caused significant lifestyle changes, such as physical inactivity and limited access to a healthy diet, which can also affect the general health of populations or countries (9–11).

Investigating seasonal variations and yearly trends in routine parameters over the past five years is of clinical and epidemiological significance. Few studies conducted in sub-Saharan Africa have considered the seasonal effects on haematological and biochemical parameters reference intervals and have shown to affect the reference intervals (1–3,12). However, an earlier study conducted in Rwanda, Uganda, and Zambia reported minimal impact of seasonality on adult clinical laboratory parameter values in these populations, suggesting that seasonal variation may not be important in evaluating adult clinical laboratory parameters (13). Despite these contradictory findings, seasonal influences on haematological and biochemical parameters can potentially impact the interpretation of laboratory results, disease diagnosis, and patient care management. In the ongoing country-tailored reference interval study project, this retrospective study has the potential to contribute to refining geographically and genetically tailored reference ranges for routine parameters in the context

of seasonality, ensuring more accurate and context-specific interpretation of laboratory results.

This study aimed to evaluate the presence and extent of seasonal variations in a comprehensive set of haematological and biochemical routine parameters in Gambian adults with no known underlying health condition and their effects on our defined reference intervals. We also examined the trending pattern of the main haematological and biochemical parameters over the past five years. By utilizing the retrospective data captured in the electronic medical record system of the Medical Research Council Unit The Gambia at LSHTM (MRCG@LSHTM) clinical laboratories, we provided a description of these variations and their potential clinical implications.

Method

Study Setting

The study was conducted in the ISO15189:2012 and good clinical laboratory practice accredited clinical diagnostic laboratory hosted at the Kuyateh Building at MRCG@LSHTM, which is located in the Kanifing municipality in the greater Banjul area of The Gambia (14-16). The diagnostic laboratory is responsible for providing standard and quality-oriented diagnostic services to MRCG@LSHTM staff, clinical trial projects, as well as government and private hospitals. Staff employed by MRCG@LSHTM underwent routine medical examinations at the clinical diagnostic laboratory, and their haematological and biochemical results data were captured in an electronic medical record system database.

Study Design and Data Extraction

This retrospective study retrieved haematological full blood count results data and

biochemical results data from 1st January 2018 to 31st December 2022 from the MRCG@LSHTM electronic medical record system database. In this study, we focused on data from individuals with no known underlying health condition who visited the clinical laboratory department for routine medical examinations or assessments. After being granted access on the 1st of January 2023, we retrieved 503 full blood count data and 772 biochemical data from the database. Following data cleaning, we excluded 10 records (n=10) from the full blood count haematological dataset and 129 records (n=129) from the biochemical dataset. As a result, our final sample size was 493 for the haematological data and 643 for the biochemical data. The extracted haematological full blood count data included haemoglobin, red blood cells (RBC), platelets, and white blood cells (WBC) and differentials results data. The extracted biochemical data comprised serum Gamma-glutamyl transpeptidase (GGT), Sodium, Potassium, Urea, Creatinine, Aspartate aminotransferase (AST), Alanine Aminotransferase (ALT), Total Protein (TP), Albumin (ALB), and Globulin results.

During the data extraction, we considered the age range of 18-63 years, in accordance with the employment age range outlined in MRCG@LSHTM policies. As such, those records that fall outside of 18 to 63 age range were excluded from the final data for analysis (Fig 1). We excluded data from the study if the electronic medical records showed positive serological results for Human Immunodeficiency Virus (HIV) and Hepatitis B. Furthermore, data from pregnant women were excluded based on clinical information available in the electronic system.

Malaria microscopy, a gold-standard diagnostic test for malaria diagnosis, is part of the routine medical examination test panel. We also excluded all the records with malaria positive results. However, all of the haematological and biochemical data showed malaria microscopy negative.

Fig 1: Flow chart demonstrating the data retrieval process and excluded records . The excluded records were from individuals whose age fall outside our specified age range of 18 to 63 years or the records that showed HIV/Hepatitis B positive, and Malaria positive.

Ethics Approval and Consent to Participate

This study was approved by the Gambia Government/MRCG Joint Ethics Committee (Project ID/Ethics ref: L2023.E03). Since all data retrieved from the database were anonymized and coded, informed consent was waived by the Ethics Committee. All methods were performed in accordance with the Declaration of Helsinki.

Statistics

The data were retrieved in Microsoft Excel and analysed using the R package to obtain summary statistics, including the mean, median, and plots. The Shapiro test was validated by Q-Q plot, and a normal distribution bell-shaped curve was used to study the distribution pattern of our data across the parameters. A box plot was used to assess the presence of outliers in datasets. Reference intervals were determined based on the 2.5th and 97.5th percentiles after eliminating outliers in our data using interquartile range calculations (25th - 1.5 * IQR; 75th - 1.5 * IQR). The comparison between the dry and wet season medians was performed by nonparametric Wilcox Rank test in R. Multivariate analysis using a generalized linear modelling approach in R was used to model the association between explanatory variables (season, age, gender, and year) and haematological and biochemical parameters. We further compared the medians of 2018 to 2021 using the Dunn test in R to generate Kruskal-Wallis chi-square and p-value results.

Results

From this study, 493 haematological full blood count data (N = 493) were analysed, including RBC, HB, PLT, and WBC differentials (Table 1). Additionally, 643 biochemistry laboratory data were analysed, including liver enzymes, sodium, potassium, urea, and creatinine (Table 1). The age range for both haematological and biochemistry data was 18 to 63 years. The mean age of haematological data was 31 years, with a median age of 28 years. For biochemistry data, the mean age was 36 years, with a median age of 33 years. However, there was no significant difference between the mean and median ages of the two datasets (Table 1).

Over the five-year period from 2018 to 2022, the dry and wet seasons were defined based on the visit date for sample collection at the clinical laboratory department. The wet season was represented by visits from June to October, while the dry season was represented by visits from November to May. Based on the visit data, 55% of the full blood count data and 58% of the biochemistry laboratory data were generated in the dry season (Table 1).

Unlike the biochemistry laboratory data, which showed almost equal gender proportions, there was a gender disparity in the haematological laboratory data, with 60% of the routine medical examination full blood count data came from the male population (Table 1).

Table 1: **Summary table of characteristics of sample population**

**Explanatory variables**	**Characteristics**	**Summary N(%)**
**Haematology laboratory**		
Season (N=493)	Dry (n)	271 (55.0)
	Wet (n)	222 (45.0)

Gender (N=493)	Male (n)	300 (60.85)
	Female (n)	193 (39.15)
Dry Season (N=271)	Male (n)	175 (64.58)
	Female (n)	96 (35.42)
Wet Season (N=222)	Male (n)	125 (56.31)
	Female (n)	97 (43.69)
Age	Age range	18-63
	Age (Median)	28.00
	Age (Mean)	30.54
**Biochemistry Laboratory**		
Season (N=643)	Dry (n)	373(58.01)
	Wet(n)	270(41.99)
Dry Season (N=373)	Male(n)	183(49.06)
	Female (n)	190(50.94)
Wet Season (N=270)	Male(n)	148(54.81)
	Female(n)	122(45.19)
Gender (N= 643)	Male (n)	339 (52.72)
	Female(n)	304(47.28)
Age	Age range	18-63
	Age (Median)	33
	Age (Mean)	36.16

Seasonal and Yearly Effects and Reference Intervals

Despite the primary focus was to assess the seasonal effects on the haematological and biochemical parameters, as well as analyse the association between explanatory variables (gender and age) and these parameters (Table 2). Using generalised linear modelling (GLM), we found that seasonality was associated with various haematological indices (HB: 0.014(0.015), P<0.05; RBC: -0.184(0.061), P<0.003; PLTs: 17.47 (6.86), p<0.01; WBC: 0.243(0.163), p = 0.0014; neutrophils: 0.271(0.131), P<0.05; and lymphocytes: -0.003(0.066), P= 0.01). However in the biochemical cohort, the biochemical measurements were not affected by the explanatory variables of dry and wet seasons.

In haematological cohort, age was found to have an association with certain parameters, including haemoglobin (0.028(0.011); P = 0.011), platelets (-0.918(0.44): P = 0.039). In the biochemical cohort, only creatinine (1.202(0.472); P<0.01), and total protein (-0.117(0.048); P<0.05) were found to have an association with age (Table 2). Furthermore, several haematological indices, except for monocytes and eosinophils, were associated with gender. In the biochemical cohort, only sodium (0.680(0.323); P= 0.04) was associated with gender.

We assessed the association of the year of visit on various parameters in healthy individuals (Table 2). Using the GLM approach, we found significant associations between the year of visit and haematological parameters such as haemoglobin (0.156(0.079): P<0.05), RBC (0.080(0.034): P<0.05), platelets (7.861(3.16); P<0.05). In the biochemical cohort, biochemical measurements such as sodium (-0.93(0.31); P<0.05), potassium (-0.107(0.041); P=0.013), albumin (-0.89(0.43); P=0.04), and globulin (1.33(0.50); P=0.01) were found to be associated with the year explanatory variable.

Table 2: Association study of explanatory variables season, age and gender, and all the haematological and biochemical parameters. The association was determined by estimated effects on the median (SE) value of the parameters. P value <0.05; Standard Error of the estimate intercept (SE)

Laboratory parameters	Season[a] Estimate(Std. Error); P value <0.05	Age (Estimate(Std. Error); P value <0.05	Gender[b] (Estimate(Std. Error); P value <0.05	Year (Estimate(Std. Error); P value <0.05
HB (g/dL)	**0.014(0.015); p<0.05**	**0.028(0.011); P =0.011**	**2.355(0.126); P<0.0001**	**0.156(0.079): P<0.05**
RBCa(106/uL)	**-0.184(0.061); p<0.05**	0.004(0.003); P=0.287	**0.750(0.049); P<0.0001**	**0.080(0.034): P<0.05**
PLT (103/uL)	**17.47 (6.86); p<0.01**	**-0.918(0.44): P = 0.039**	**-60.19(6.276); P<0.0001**	**7.861(3.16); P<0.05**
WBC (103/uL)	**0.243(0.163); P<0.01**	-0.006(0.011); P = 0.566	**-0.929(0.158); P<0.0001**	0.030(0.071) P>0.67
Neutrophils (103/uL)	**0.271(0.131); P<0.05**	-0.009(0.008); P=0.295	**-0.624(0.129); P<0.0001**	-0.027(0.05); P=0.61
Lymphocytes (103/uL)	**-0.003(0.066);P< 0.01**	0.006(0.004); P= 0.169	**-0.288(0.0654); P<0.0001**	0.038(0.03); P= 0.22
Monocytes (103/uL)	-0.004(0.165); P=0.798	-0.002(0.001); P=0.092	-0.027(0.017); P = 0.11	**0.037(0.007); P<0.05**
Eosinophils (103/uL)	-0.021(0.023); P=0.359	-0.001(0.001); P=0.380)	0.011(0.023); P= 0.623	-0.01(0.01); P=0.139

Sodium(mmol/L)	-0.238(0.330);P =0.471	0.0188(0.013); P=0.156	**0.680(0.323); P=0.04**	**-0.93(0.31); P<0.05**
GGT (U/L)	0.213(7.31); P=0.977	0.227(0.241); P= 0.347	10.696(7.27); P=0.142	-0.43(1.39); P=0.76
Potassium (mmol/L)	0.020(0.039);P=0.6143	0.001(0.001): P=0.424	0.065(0.039); P=0.098	**-0.11(0.04); P=0.013**
Urea (mmol/L)	-4.128(0.259);P=0.112	0.064(0.040); P=0.113	1.225(1.301); P=0.347	-0.11(0.136); P= 0.42
Creatinine (umol/L)	-18.13(14.88);P=0.224	**1.202(0.472); P<0.01**	28.15(15.23); P=0.066	0.62(2.00); P=0.76
AST (U/L)	-2.045(2.256);P<0.344	-0.066(0.069); P= 0.597	0.566(0.256); P=0.798	-0.56(0.65); P= 0.40
ALT (U/L)	-1.619(2.60); P=0.534	0.031(0.089); P=0.729	1.244(2.846); P=0.662	-1.69(1.12); P=0.14
Albumin (g/dL)	-1.663(0.863); P= 0.06	-0.052(0.029); P=0.08	2.028(0.814); P=0.07	**-0.89(0.43); P=0.04**
Total Protein (g/dL)	-2.142(1.465); P=0.148	**-0.117(0.048=9); P<0.05**	1.017(1.408); P=0.472	-0.51(0.64); P=0.43
AST (U/L)	-0.536(1.228); P=0.663	-0.064(0.041); P= 0.12	-1.057(1.168); 0.367	**1.33(0.50); P=0.01**

[a]Wet Season(reference) is compared to Dry Season; [c]Male (reference) is compared to Female gender;

We determined reference intervals nonparametrically based on the 2.5th and 97.5th percentiles for both haematological indices and biochemical measurements (Table 3). To assess the effects of seasonality, we compared the median values between the dry season and the wet season for each year from 2018 to 2022. Although the differences were not statistically significant across all haematological indices (Wilcoxon test, P>0.05), the median values for platelets, as well as WBC and differentials, were slightly higher in the wet season compared to the dry season. Conversely, the median values for HB and RBC were lower in the wet season. Similarly, the median values for all the biochemical measurements were not statistically significant (Wilcoxon test, P>0.05). However, potassium had a slightly higher median in the dry season (median=4.20) compared to the wet season (median=4.10).

We also evaluated the effects of seasonality on the reference intervals derived from our haematological and biochemical study data. In haematological data, we observed changes in reference intervals from the dry season to the wet season were not significant (Wilcoxon test, P>0.05). However, we observed a slight reduction in the upper limit of reference intervals in the wet season for HB and RBC haematological indices, and a slight increase in the upper limit of WBC and differentials. Similarly, in biochemical measurements, we also found the changes in reference intervals from the dry season to the wet season not statistically significant (Wilcoxon test, P>0.05).

Table 3: The effect of exploratory seasonal factor on the reference ranges of the haematological and biochemical parameters (Wilcoxon test P>0.05).

**Laboratory parameters**	**N**	**Median (Reference Range)**	**N**	**Median (Dry Season)**	**N**	**Wet Season**
HB (g/dL)	461	13.90(11.0-16.5)	258	14.0(11.34-16.36)	206	13.70(10.70-16.29)
RBC[a](10^6/uL)	444	4.87(3.90-5.85)	238	4.95(4.06-5.89)	201	4.77(3.77-5.76)
PLT (10^3/uL)	433	244.5(158.6-346.2)	238	237(156.9-338.2)	197	248(163.5-361.1)
WBC (10^3/uL)	437	5.40(3.52-7.65)	245	5.34(3.51-7.60)	194	5.34(3.52-7.80)
Neutrophils (10^3/uL)	426	2.55(1.34-4.05)	235	2.47(1.32-3.81)	194	2.49(1.40-4.55)
Lymphocytes (10^3/uL)	418	2.15(1.33-2.96)	232	2.14(1.30-3.065)	192	2.14(1.33-2.93)
Monocytes (10^3/uL)	416	0.43(0.25-0.64)	230	0.42(0.25-0.62)	190	0.43(0.25-0.64)
Eosinophils (10^3/uL)	335	0.13(0.13-0.31)	201	0.14(0.02-0.36)	138	0.11(0.01-0.26)
Basophils (10^3/uL)	350	0.04(0.01-0.07)	185	0.03(0.02-0.06)	146	0.04(0.02-0.07)
Sodium (mmol/L)	384	140(136-144)	215	140(137-144)		140(136-144)
Potassium (mmol/L)	498	4.12(3.6-4.7)	235	4.20(3.6-4.7)	152	4.10(3.70-4.50)
GGT (U/L)	240	26(11.00-52.025)	134	26(10.23-51.03)	106	26(12.78-53.00)

Creatinine (umol/L)	500	75.2(50.48-107.05)	289	75(49.00-107.08)	211	77(52.93-106.68)
Urea (mmol/L)	507	3.1(1.7-4.9)	381	3.1(1.80-4.7)	217	3.1(1.70-4.9)
AST (U/L)	354	20(12-32)	211	20(12-34.5)	138	20(12.43-29.00)
ALT (U/L)	234	17(7.0-33.0)	130	17(6.23-33.78)	104	17(8.15-31.43)
Albumin (g/dL)	236	40(34-47)	150	40(34.00-47.00)	82	40(34.03-45.00)
Total Protein (g/dL)	336	76(67.00-83.63)	195	76(68.00-85.00)	147	76(67.00-83.35)
Globulin (g/dL)	203	35(27.0-42.00)	116	35(27-42.13)	73	35(29.8-40.0)

[a]Mean value was used for RBC parameter because the data was normally distributed

Monthly and Yearly Pattern in Parameters

We further analysed patterns based on changes in the monthly and yearly median of haematological and biochemical parameters (Fig 2 and S1-S2 Fig). These figures demonstrated a change in the pattern of our monthly median of haematological indices and biochemical measurements from January to December over five year period. From these our study results (Fig 2, S1-S2 Fig), we observed that laboratory parameters, including HB, RBC, HCT/PCV showed seasonal patterns throughout the year, with levels being normal to slightly low during the dry season and normal to high during the wet season. Likewise we observed a seasonal pattern for total WBC, neutrophils, lymphocytes, and platelets from November to March with normal to slightly high median . In terms of biochemistry parameters, liver enzymes (AST, ALT, GGT, total protein) and potassium were generally high in the wet season (June to October) but moderately low in the dry season.

Finally, we assessed the changes in median values of the parameters from 2018 to 2022 using the boxplots in Fig 3 and 4. We followed this with a statistical significance test using the non-parametric Kruskal-Wallis test. Overall, no statistically significant difference was found (Kruskal-Wallis chi-squared = 0.1148, p-value = 1) among the median values of all the haematological indices and biochemical measurements in the last five years (2018 to 2022). In haematological data, the median values for HB and RBC parameters decreased in 2020, while the median values for total WBC count, absolute neutrophils count, and platelets increased slightly. As for biochemical parameters, there was no clear pattern except for AST and ALT, which showed a downward trend from 2018 to 2022.

Fig 2: Monthly pattern of haematological and biochemical parameters from 1st January 2018 to 31st December 2022 in five year period. Haemoglobin (HB); Red blood cells (RBC); White blood cells (WBC); Platelets (PLT); Neutrophils (NEUTRO); Lymphocytes (LYMPH); Mean cell volume (MCV); Packed cell volume (PCV); Eosinophils (EOS); Basophil (BASO); Aspartate aminotransferase (AST); Alanine transaminase (ALT); Gamma-glutamyl transferase (GGT).

Fig 3: Boxplot showing pattern/trend of haematological parameters in the last five years. The results displayed in medians (Kruskal-Wallis chi-squared = 0.063, p-value = 1).

Fig 4: Boxplot showing pattern/trend of biochemical parameters in the last five years. Aspartate aminotransferase (AST); Alanine transaminase (ALT); Gamma-glutamyl transferase (GGT). (Kruskal-Wallis chi-squared = 0.8433, p-value = 0.93)

Discussion

The study examined the effects of seasonality on haematological indices and biochemical measurements, as well as the derived reference intervals. Seasonal variations can be influenced by factors such as changes in environmental temperature and humidity, diet, physical activity, and seasonality of certain diseases like malaria. Overall, no statistically significant differences were found between the median values generated in the dry and wet seasons as well as reference intervals generated between the dry and wet season, indicating that there was no seasonal effect on the reference intervals derived from our study data. The reference intervals derived from our study data were comparable to the results reported by Adetifa et al in 2009, which focused on Gambian adults without any known underlying health conditions during a TB contact tracing study (38). Notably, there was a slight difference in the upper limit of neutrophils reference intervals between the dry season (1.32-3.81) and wet season (1.40-4.55), suggesting the need for more informative reference intervals that consider seasonality. These seasonal variations should be taken into consideration, especially in research studies or clinical trials where patients are monitored at different times of the year. Being aware of these subtle seasonal variations can help prevent erroneous conclusions based on study data.

To further investigate the seasonal variation pattern of these parameters from January to December, we observed moderately normal to slightly higher median pattern for haemoglobin and RBC during the wet season. Haemoglobin and RBC parameters are crucial for diagnosing anaemia in clinical settings. It has been reported that haemoglobin levels in individuals without any known underlying health conditions can vary due to seasonal factors such as changes in diet, including increased consumption of iron-rich foods during certain seasons, as well as asymptomatic infection (22). In the Gambia, the wet season is

characterized by the abundant availability of iron-rich foods, which could explain the higher levels of haemoglobin and RBCs during this period (21). Besides, the moderately normal to high HCT seasonal variation patterns in the wet season also demonstrate the high haemoglobin and RBC medians in the wet season could be attributed to low fluid intake during the wet season which accompanies low temperature and humidity (21).

The seasonal variations in WBC and platelets were observed in our study from November to March. In the Gambia, changes in the environment during the wet season, such as increased rainfall and cold weather from November to March, favour the transmission of malaria parasites, which typically peaks in November, as well as respiratory infections (24–29). Our study supports previous findings regarding the seasonal effects on WBC and differentials (1). Infectious diseases like malaria tend to flourish during the wet season, likely due to environmental factors such as heavy rainfall leading to stagnant water and waterlogged areas (30-33). This can potentially lead to an increase in mean WBC count and differentials in the general population due to low-grade inflammation in the community caused by malaria transmission and viral illnesses (3,10). The seasonal variation in WBC and differentials could be impacted by malaria parasite infection. However, our study records showed malaria microscopy was negative for both haematological cohort and biochemical cohort data retrieved electronically.

Similar to hemoglobin and RBCs, the monthly pattern of liver enzymes and potassium changed based on seasonality. Our findings are aligned with earlier studies that have demonstrated how seasonal changes in diet and fluid intake can impact biochemical measurements, especially electrolytes. The seasonal effects on these biochemical measurements may be attributed to the temperature difference between the wet and dry seasons (34–36). A reference interval study conducted in Guinea also reported seasonal

effects on biochemical parameters, particularly liver enzymes (31). Despite reports of dehydration being more common during the dry season and potentially affecting biochemical measurements like serum creatinine and urea levels, our study found that median values for urea and creatinine remained relatively normal across both seasons (37).

The first case of COVID-19 in the Gambia was identified on March 17, 2020, which eventually led to a government-mandated lockdown and restrictions in an effort to curtail community transmission (40). While there have been no significant differences in the median values for all parameters during the past five years, the decrease in median values for haemoglobin and RBCs in 2020, along with the increase in median value for total WBC, might be attributed to the effects of the COVID-19 pandemic on lifestyles. A study conducted by Werf et al. in 2021 supports this, as it reported that the COVID-19 pandemic has had an impact on lifestyles in the Netherlands(39). This impact includes limited access to healthy diets and fresh fruits and vegetables, changes in eating patterns, and reduced physical activity which could have an impact on the overall health of the public. Hence, further qualitative study is needed to shed light on the impact of COVID-19 pandemic on life styles in the Gambia. Alarmingly, our study also revealed a consistent decline in the median values of AST and ALT, warranting further investigation. Public health authorities should closely monitor the trends in haematological indices and biochemical measurements to assess the lasting impact of the pandemic on the overall public health.

As observed in numerous retrospective studies, our study is also subject to limitations that restrict our control over certain factors. These factors include haemoglobin genotype status, body size, smoking, alcohol consumption, exercise, and contraceptive pill use, all of which have the potential to influence the outcome (17). Additionally, our sample size was constrained by the available data, and we were unable to obtain additional data to enhance

statistical power (18,19). These limitations could be addressed through the implementation of a well-designed prospective study conducted on a general population, following the approach employed by Okebe et al. in 2016. Despite these limitations, we maintain that the results of our study are accurate, as our sample size was considerable. Therefore, our study remains representative and supports the current prospective reference intervals study that will provide a more comprehensive sampling and data stratification reflecting both dry and wet seasons.

Conclusion

Our study found no significant seasonal effects on the reference intervals for haematological and biochemical parameters, despite the identification of certain parameters that exhibited some association with seasonality. However, we did observe seasonal variations in specific parameters such as haemoglobin, red blood cells, liver enzymes, and potassium levels from January to December. Furthermore, we did not observe significant yearly variations in the median values of the all haematological indices and biochemical measurements. Therefore, our findings emphasize the importance of considering seasonal variations in haematological indices and biochemical measurements when interpreting laboratory results and making diagnostic decisions, as well as when recruiting participants for clinical trial projects. Our study should be followed by well-designed context-specific studies that employ standardized sampling and analytical methods within our geographical setting.

Acknowledgements

Not Applicable

Availability of Data and Materials

The datasets used and/or analysed during the current study are available and submitted with manuscript

Financial Disclosure Statement

This project received no specific funding for this work.

Consent for publication: Not applicable

Competing Interest

The authors have declared that no competing interests exist.

Abbreviations

COVID-19: Coronavirus Disease 2019

LSHTM: London School of Hygiene and Tropical Medicine

MRCG@LSHTM: Medical Research Council Unit The Gambia at LSHTM

HIV: Human Immunodeficiency Virus

GGT: Gamma-glutamyl transpeptidase

AST: Aspartate aminotransferase

ALT: Alanine Aminotransferase

TP: Total Protein

ALB: Albumin

WBC: White Blood Cells

RBC: Red Blood Cells

HB: Haemoglobin,

PLT: Platelets

GLM: Generalised Linear Modelling

Author's Contributions

MD and AAN designed the study and drafted the manuscript. MD, MDD, FS, RB, NS, MB, and SU performed the laboratory analysis, data retrieval and performed data arrangements. MD and AAN performed the statistical analysis. All authors reviewed and approved the final manuscript.

References

1. Bæk O, Jensen KJ, Andersen A, Balé C, Martins C, Biering-Sørensen S, et al. Seasonal and sex-specific variations in haematological parameters in 4 to 5.5-month-old infants in Guinea-Bissau, West Africa. Trans R Soc Trop Med Hyg. 2017;111(1).

2. Béavogui AH, Touré AA, Camara D, Doumbouya A, Sylla MM, Touré S, et al. Haematological and Biochemical Reference Values for Healthy Population of Maferinyah Rural Community in Guinea. Biomed Res Int. 2020;2020.

3. Okebe J, Mwesigwa J, Agbla SC, Sanya-Isijola F, Abubakar I, D'Alessandro U, et al. Seasonal variation in haematological and biochemical reference values for healthy young children in The Gambia. BMC Pediatr. 2016;16(1).

4. Gidlow DA, Church JF, Clayton BE. Seasonal Variations in Haematological and Biochemical Parameters. Ann Clin Biochem. 1986;23(3).

5. Marriott BM. Nutritional Needs in Hot Environments: Applications for military personnel in Field Operations. Nutritional Needs in Hot Environments. 1993.

6. Chan CB, Ryan DA. Assessing the effects of weather conditions on physical activity participation using objective measures. Vol. 6, International Journal of Environmental Research and Public Health. 2009.

7. Pasiakos SM. Nutritional Requirements for Sustaining Health and Performance during Exposure to Extreme Environments. Vol. 40, Annual Review of Nutrition. 2020.

8. Turrisi TB, Bittel KM, West AB, Hojjatinia S, Hojjatinia S, Mama SK, et al. Seasons, weather, and device-measured movement behaviors: a scoping review from 2006 to 2020. Vol. 18, International Journal of Behavioral Nutrition and Physical Activity. 2021.

9. Akinbo FO, Okaka CE, Omoregie R. Seasonal Variation of Intestinal Parasitic Infections Among HIV-Positive Patients in Benin City, Nigeria. Ethiopian journal of health sciences. 2011;21(3).

10. Mwesigwa J, Okebe J, Affara M, Di Tanna GL, Nwakanma D, Janha O, et al. On-going malaria transmission in The Gambia despite high coverage of control interventions: A nationwide cross-sectional survey. Malaria Journal. 2015;14(1).

11. Collins KA, Ceesay S, Drammeh S, Jaiteh FK, Guery MA, Lanke K, et al. A Cohort Study on the Duration of Plasmodium falciparum Infections During the Dry Season in The Gambia. Journal of Infectious Diseases. 2022;226(1).

12. Serena V, Alessandro M, Maurizio, Nadia T, Valeria S, Eleonora C, et al. Baseline haematological and biochemical reference values for healthy male adults from mali. Pan African Medical Journal. 2019;32.

13. Ruzagira E, Abaasa A, Karita E, Mulenga J, Kilembe W, Allen S, et al. Effect of seasonal variation on adult clinical laboratory parameters in Rwanda, Zambia, and Uganda: Implications for HIV biomedical prevention trials. PLoS ONE. 2014;9(8).

14. Ezzelle J, Rodriguez-Chavez IR, Darden JM, Stirewalt M, Kunwar N, Hitchcock R, et al. Guidelines on good clinical laboratory practice: Bridging operations between research and clinical research laboratories. Vol. 46, Journal of Pharmaceutical and Biomedical Analysis. 2008.

15. Joshi RK, Sarangi SC, Mohapatra S, Mallick S, Datta SK. A Comparative Review of ICMR, WHO, and EMA Guidelines for Good Clinical Laboratory Practices. Journal of Laboratory Physicians. 2023;15(02).

16. International Organization for standardization. ISO 15189:2012 - Medical laboratories-Requirements for quality and competence. International Organization for standardization ISO. 2012;2012.

17. Talari K, Goyal M. Retrospective studies - Utility and caveats. Journal of the Royal College of Physicians of Edinburgh. 2020;50(4).

18. Fitzner K, Heckinger E. Sample size calculation and power analysis: a quick review. Diabetes Educ. 2010;36(5):701-7. doi: 10.1177/0145721710380791..

19. Kim J, Seo BS. How to calculate sample size and why. Clinics in Orthopedic Surgery. 2013;5(3).

20. Critchley J, Bates I. Haemoglobin colour scale for anaemia diagnosis where there is no laboratory: A systematic review. International Journal of Epidemiology. 2005;34(6).

21. World Health Organization. Countdown to 2015 decade report (2000-2010) with country profiles: taking stock of maternal, newborn and child survival. Maternal, Newborn & Child Survival Countdown to 2015. 2015;

22. Semagn BE, Gebreegziabher ZA, Abebaw WA. Iron-rich food consumption and associated factors among children aged 6–23 months in Sierra Leone: multi-level logistic regression analysis. BMC Public Health. 2023;23(1).

23. Ayanful-Torgby R, Quashie NB, Boampong JN, Williamson KC, Amoah LE. Seasonal variations in Plasmodium falciparum parasite prevalence assessed by varying diagnostic tests in asymptomatic children in southern Ghana. PLoS ONE. 2018;13(6).

24. Chowdhury FR, Ibrahim QSU, Shafiqul Bari M, Jahangir Alam MM, Dunachie SJ, Rodriguez-Morales AJ, et al. The association between temperature, rainfall and humidity with common climate-sensitive infectious diseases in Bangladesh. PLoS ONE. 2018;13(6).

25. Prentice AM, Mendoza YA, Pereira D, Cerami C, Wegmuller R, Constable A, et al. Dietary strategies for improving iron status: Balancing safety and efficacy. Nutrition Reviews. 2017;75(1).

26. Maes M, Stevens W, Scharpe S, Bosmans E, De Meyer F, D'Hondt P, et al. Seasonal variation in peripheral blood leukocyte subsets and in serum interleukin-6, and soluble interleukin-2 and-6 receptor concentrations in normal volunteers. Experientia. 1994;50(9).

27. Ruul A, Saar B. Anaemia of chronic disease. Eesti Arst. 2015;94(9).

28. Kurkina N V., Gorshenina EI, Chegodaeva L V., Polagimova A V. Anemia of Chronic Diseases. Klinicheskaya Onkogematologiya/Clinical Oncohematology. 2021;14(3).

29. Fraenkel PG. Understanding anemia of chronic disease. Hematology (United States). 2015;2015(1).

30. Fares A. Factors influencing the seasonal patterns of infectious diseases. International Journal of Preventive Medicine. 2013;4(2).

31. Abdoul Habib Béavogui, Almamy Amara Touré, Daouda Camara, Abdoulaye Doumbouya, Malick Minkael Sylla, Sékou Touré, Ahmadou Hamidou Togo, Mamadou Saliou Diallo, Alexandre Delamou, Issaka Sagara, Alassane Dicko, Abdoulaye Djimdé, "Haematological and Biochemical Reference Values for Healthy Population of Maferinyah Rural Community in Guinea", BioMed Research International. 2020;2020. https://doi.org/10.1155/2020/8605485

32. Mandala W, Munyenyembe A, Sulani I, Soko M, Mallewa J, Hiestand J. Acute Malaria in Malawian Children and Adults is Characterized by Thrombocytopenia That Normalizes in Convalescence. Journal of Blood Medicine. 2022;13.

33. Bhatia I, Sharma A, Guleria S, Sharma A, Jaswal RS, Vaid A, et al. Thrombocytopenia in Children: A Large Prospective Study on Clinical Manifestations, Seasonal Variation, Etiology, and Outcome. Journal of Association of Physicians of India. 2023;71(3).

34. Kailakov AM. Seasonal fluctuations in the content of some electrolytes in patients with diabetes mellitus. Problemy Endokrinologii. 1979;25(2).

35. Swingler G, Power D. Seasonal plasma electrolyte fluctuations in childhood diarrhoea. Archives of Disease in Childhood. 2002;87(5).

36. Koyama T, Makinouchi R, Machida S, Matsui K, Shibagaki Y, Imai N. Seasonal Changes in the Prevalence of Hyperkalemia in the Emergency Department: A Single Center Study. Medicina (Lithuania). 2022;58(2).

37. Ephraim RKD, Asamoah CA, Abaka-Yawson A, Kwadzokpui PK, Adusei S. Climate change causes changes in biochemical markers of kidney disease. BMC Nephrology. 2020;21(1).

38. Adetifa IM, Hill PC, Jeffries DJ, Jackson-Sillah D, Ibanga HB, Bah G, Donkor S, Corrah T, Adegbola RA. Haematological values from a Gambian cohort--possible reference range for a West African population. Int J Lab Hematol. 2009 Dec;31(6):615-22. doi: 10.1111/j.1751-553X.2008.01087.x. Epub 2008 Jul 10. PMID: 18631172.

39. van der Werf, E.T., Busch, M., Jong, M.C. et al. Lifestyle changes during the first wave of the COVID-19 pandemic: a cross-sectional survey in the Netherlands. BMC Public Health 21, 1226 (2021). https://doi.org/10.1186/s12889-021-11264-z

40. Abatan B, Agboghoroma O, Akemoke F, Antonio M, Awokola B, Bittaye M, Bojang A, Bojang K, Brotherton H, Cerami C, Clarke E, D'Alessandro U, de Silva T, Drammeh M, Forrest K, Hofmann N, Jagne S, Jah H, Jarju S, Jaye A, Jobe M, Kampmann B, Manjang B, Martinez-Alvarez M, Mohammed N, Nadjm B, Ndiath MO, Nkereuwem E, Nwakanma D, Oko F, Okoh E, Okomo U, Olatunji Y, Oriero E, Prentice AM, Roberts C, Roca A, Sabally B, Sambou S, Samateh A, Secka O, Sesay AK, Singhateh Y, Susso B, Usuf E, Vilane A, Wariri O. Intense and Mild First Epidemic Wave of Coronavirus Disease, The Gambia. Emerg Infect Dis. 2021; 27(8):2064-2072. doi: 10.3201/eid2708.204954.

Supporting Information Files

S1 File: Study Raw Data

S2 File: L2023.E03_Dibbasey_Ethics_Waiver_1

S1 Fig: Haematological indices from January to December in five year period. The point showed monthly medians in five year period.

S2 Fig: Biochemical measurements from January to December in five year period. The point showed monthly medians in five year period.

Figure 1

Click here to access/download;Figure;Fig 1.tif

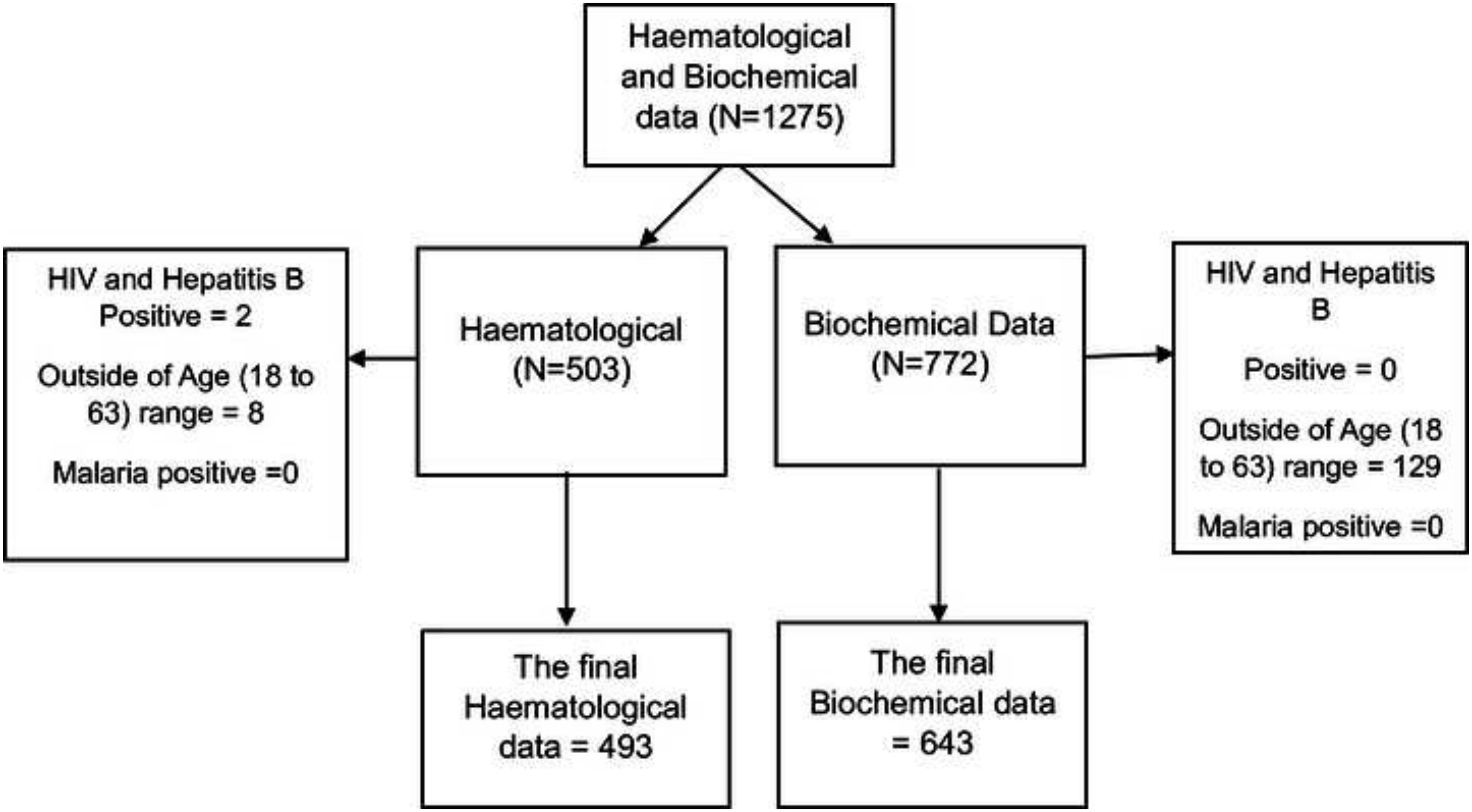

Figure 2

Click here to access/download;Figure;Fig 2.tif

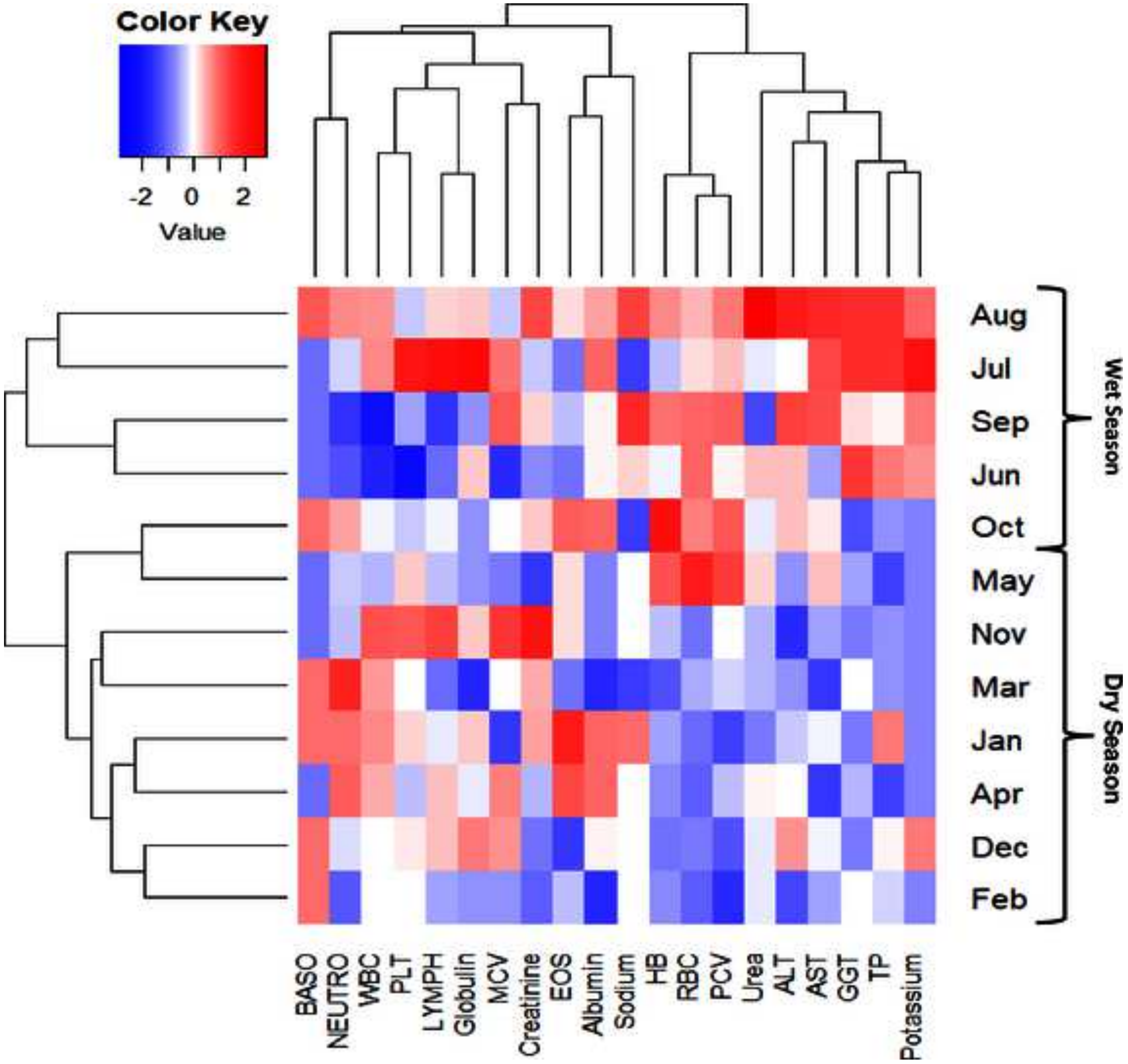

Figure 3 Click here to access/download;Figure;Fig 3.tif

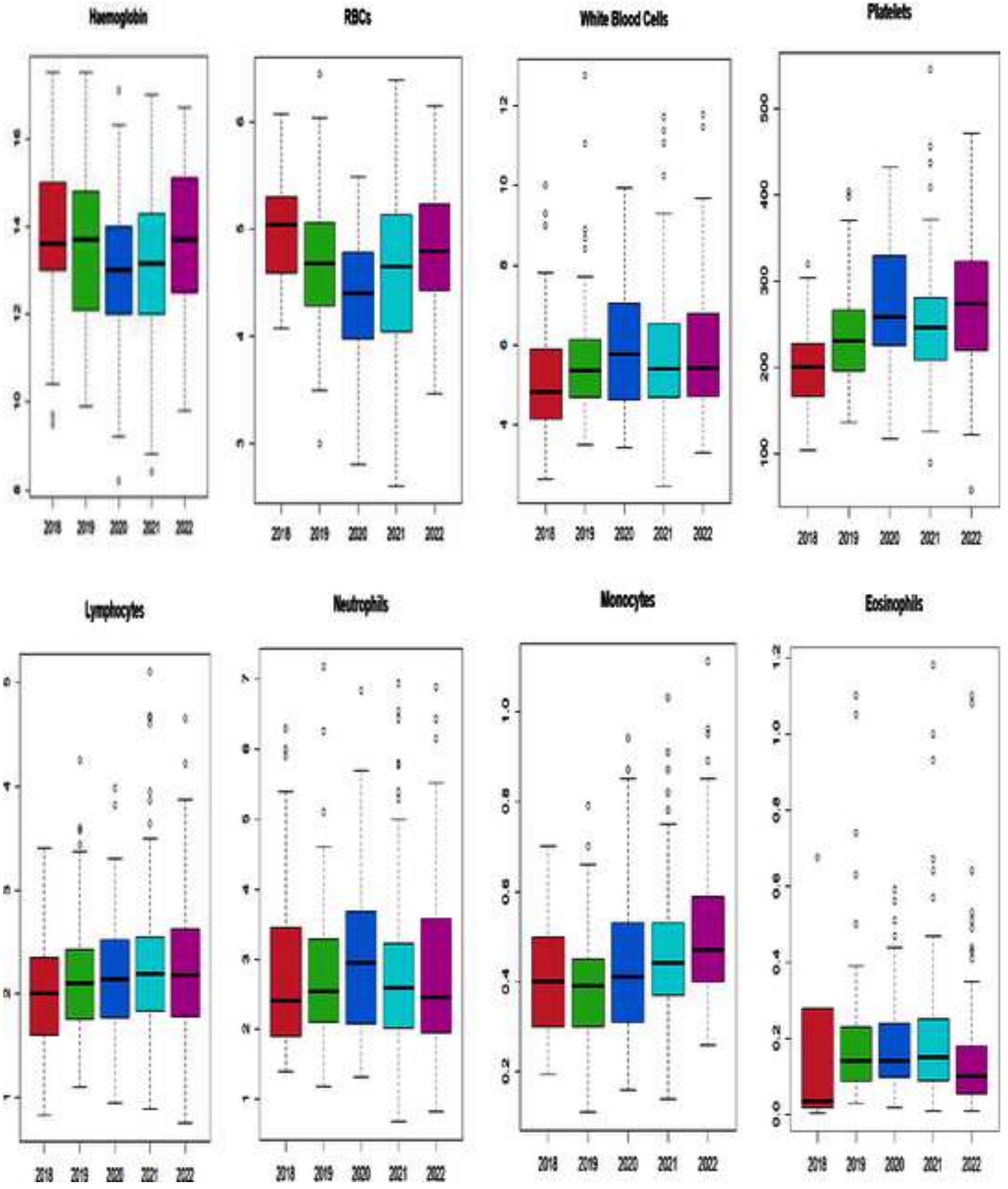

Figure 4

Click here to access/download;Figure;Fig 4.tif

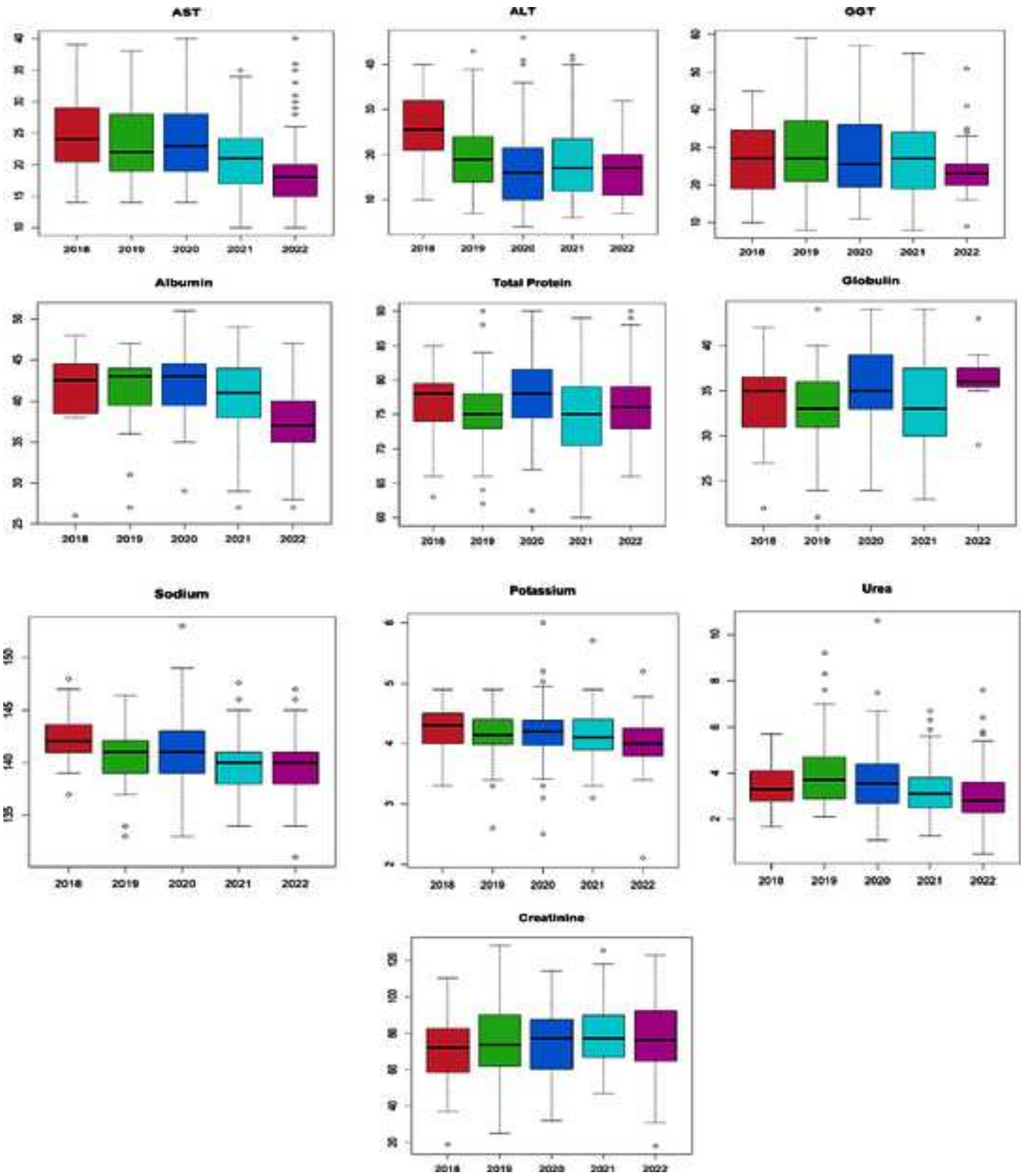

Click here to access/download
Supporting Information
S2_File.pdf

Study Raw Data

Click here to access/download
Supporting Information
S1_File.xlsx

Click here to access/download
Supporting Information
S1_Fig (1).tif

Click here to access/download
Supporting Information
S2_Fig.tif

Seasonal variations in haematological and biochemical parameters of healthy Gambian adults: Retrospective Study 2018-2022

Mustapha Dibbasey*[1,2], Mamudou Dahaba[1], Francess Sarfo[1], Rosyna Begum[1], Mustapha Kanteh[1], Nyima Sumareh[1], Mustapha Bakare[1], Solomon Umukoro[1], **Alfred Amambua-Ngwa**[1,3]

[1]Medical Research Council the Gambia at London School of Hygiene and Tropical Medicine

[2]School of Biological Science, Department of Biochemistry, Cell and Molecular Biology, University of Ghana, Ghana, Accra, Legon

[3]London School of Hygiene and Tropical Medicine

*Corresponding Author: Mustapha Dibbasey (lshmd4@lshtm.ac.uk; mdibbasey@mrc.gm)

Abstract

Objective: The objectives of this study are to determine the presence and effect of seasonal variations and provide insights into trend from 2018 to 2022 in a comprehensive set of routine haematological indices and biochemical measurements in Gambian adults with no known underlying health condition..

Methods: The study was conducted in the clinical diagnostic laboratory at MRCG@LSHTM. We retrieved five years of data (1st January 2018 to 31st December 2022) from an electronic database and analysed 493 full blood counts and 643biochemical data. In this study, we focused on data from individuals with no known underlying health condition who visited the clinical diagnostic laboratory for routine medical examinations or assessments.

Results: Our study found a positive association between seasonality (wet season as the reference) and HB (HB: 0.014(0.015), P<0.05), WBC (WBC: 0.243(0.163), p =0.0014), and neutrophils (neutrophils: 0.271(0.131), P<0.05) with exception to RBC (RBC: - 0.184(0.061), P< 0.003) that showed negative association. Despite the association, the seasonal effects on our derived reference intervals for haematological indices and biochemical measurements from the wet season to the dry season were not statistically significant (Wilcoxon test, P>0.05). In addition, we observed in our heatmap result that some laboratory parameters, including HB, RBC, HCT/PCV, urea, liver enzymes, and potassium, showed seasonal variation patterns throughout the year, with median levels being normal to slightly low during the dry season and normal to high during the wet season. Although no significant difference (Kruskal-Wallis chi-squared = 0.1148, p-value = 1) was found among the median values for all parameters from 2018 to 2022, further analysis showed reduced median values for HB and RBC and a high median value for total WBC in 2022. Additionally, ALT and AST parameters showed a consistent declining trend from 2018 to 2022.

Conclusion: Our study found no seasonal effects on the derived reference intervals of haematological indices and biochemical measurements. However, we observed changes in patterns for certain parameters particularly HB, RBC, liver enzymes, and potassium based on seasonality .

Keywords: Dry Season; Wet Season; Haematological Indices; Biochemical Measurements; Seasonal Variation; Seasonality

Introduction

Routine haematological and biochemical parameters are essential for assessing an individual's health and diagnosing various medical conditions(1–4). Seasonal and yearly variations in these parameters can be influenced by environmental factors such as temperature, humidity, and rainfall. Changes in environmental conditions can impact an individual's dietary preferences and physical activity levels (5–8), which in turn affect their metabolic processes and overall health. Additionally, certain diseases, including respiratory infections, malaria, and parasitic infestations, often exhibit seasonal patterns that can impact haematological and biochemical parameters, particularly total white cell count and differentials. Moreover, the COVID-19 pandemic has caused significant lifestyle changes, such as physical inactivity and limited access to a healthy diet, which can also affect the general health of populations or countries (9–11).

Investigating seasonal variations and yearly trends in routine parameters over the past five years is of clinical and epidemiological significance. Few studies conducted in sub-Saharan Africa have considered the seasonal effects on haematological and biochemical parameters reference intervals and have shown to affect the reference intervals (1–3,12). However, an earlier study conducted in Rwanda, Uganda, and Zambia reported minimal impact of seasonality on adult clinical laboratory parameter values in these populations, suggesting that seasonal variation may not be important in evaluating adult clinical laboratory parameters (13). Despite these contradictory findings, seasonal influences on haematological and biochemical parameters can potentially impact the interpretation of laboratory results, disease

diagnosis, and patient care management. In the ongoing country-tailored reference interval study project, this retrospective study has the potential to contribute to refining geographically and genetically tailored reference ranges for routine parameters in the context of seasonality, ensuring more accurate and context-specific interpretation of laboratory results.

This study aimed to evaluate the presence and extent of seasonal variations in a comprehensive set of haematological and biochemical routine parameters in Gambian adults with no known underlying health condition and their effects on our defined reference intervals. We also examined the trending pattern of the main haematological and biochemical parameters over the past five years. By utilizing the retrospective data captured in the electronic medical record system of the Medical Research Council Unit The Gambia at LSHTM (MRCG@LSHTM) clinical laboratories, we provided a description of these variations and their potential clinical implications.

Method

Study Setting

The study was conducted in the ISO15189:2012 and good clinical laboratory practice accredited clinical diagnostic laboratory hosted at the Kuyateh Building at MRCG@LSHTM, which is located in the Kanifing municipality in the greater Banjul area of The Gambia (14-16). The diagnostic laboratory is responsible for providing standard and quality-oriented diagnostic services to MRCG@LSHTM staff, clinical trial projects, as well as government and private hospitals. Staff employed by MRCG@LSHTM underwent routine medical examinations at the clinical diagnostic laboratory, and their haematological and biochemical results data were captured in an electronic medical record system database.

Study Design and Data Extraction

This retrospective study retrieved haematological full blood count results data and biochemical results data from 1st January 2018 to 31st December 2022 from the MRCG@LSHTM electronic medical record system database. In this study, we focused on data from individuals with no known underlying health condition who visited the clinical laboratory department for routine medical examinations or assessments. After being granted access on the 1st of January 2023, we retrieved 503 full blood count data and 772 biochemical data from the database. Following data cleaning, we excluded 10 records (n=10) from the full blood count haematological dataset and 129 records (n=129) from the biochemical dataset. As a result, our final sample size was 493 for the haematological data and 643 for the biochemical data. The extracted haematological full blood count data included haemoglobin, red blood cells (RBC), platelets, and white blood cells (WBC) and differentials results data. The extracted biochemical data comprised serum Gamma-glutamyl transpeptidase (GGT), Sodium, Potassium, Urea, Creatinine, Aspartate aminotransferase (AST), Alanine Aminotransferase (ALT), Total Protein (TP), Albumin (ALB), and Globulin results.

During the data extraction, we considered the age range of 18-63 years, in accordance with the employment age range outlined in MRCG@LSHTM policies. As such, those records that fall outside of 18 to 63 age range were excluded from the final data for analysis (Fig 1). We excluded data from the study if the electronic medical records showed positive serological results for Human Immunodeficiency Virus (HIV) and Hepatitis B. Furthermore, data from pregnant women were excluded based on clinical information available in the electronic system.

Malaria microscopy, a gold-standard diagnostic test for malaria diagnosis, is part of the routine medical examination test panel. We also excluded all the records with malaria

positive results. However, all of the haematological and biochemical data showed malaria microscopy negative.

Fig 1: Flow chart demonstrating the data retrieval process and excluded records . The excluded records were from individuals whose age fall outside our specified age range of 18 to 63 years or the records that showed HIV/Hepatitis B positive, and Malaria positive.

Ethics Approval and Consent to Participate

This study was approved by the Gambia Government/MRCG Joint Ethics Committee (Project ID/Ethics ref: L2023.E03). Since all data retrieved from the database were anonymized and coded, informed consent was waived by the Ethics Committee. All methods were performed in accordance with the Declaration of Helsinki.

Statistics

The data were retrieved in Microsoft Excel and analysed using the R package to obtain summary statistics, including the mean, median, and plots. The Shapiro test was validated by Q-Q plot, and a normal distribution bell-shaped curve was used to study the distribution pattern of our data across the parameters. A box plot was used to assess the presence of outliers in datasets. Reference intervals were determined based on the 2.5th and 97.5th percentiles after eliminating outliers in our data using interquartile range calculations (25th - 1.5 * IQR; 75th - 1.5 * IQR). The comparison between the dry and wet season medians was performed by nonparametric Wilcox Rank test in R. Multivariate analysis using a generalized linear modelling approach in R was used to model the association between explanatory variables (season, age, gender, and year) and haematological and biochemical parameters. We further compared the medians of 2018 to 2021 using the Dunn test in R to generate Kruskal-Wallis chi-square and p-value results.

Results

From this study, 493 haematological full blood count data (N = 493) were analysed, including RBC, HB, PLT, and WBC differentials (Table 1). Additionally, 643 biochemistry laboratory data were analysed, including liver enzymes, sodium, potassium, urea, and creatinine (Table 1). The age range for both haematological and biochemistry data was 18 to 63 years. The mean age of haematological data was 31 years, with a median age of 28 years. For biochemistry data, the mean age was 36 years, with a median age of 33 years. However, there was no significant difference between the mean and median ages of the two datasets (Table 1).

Over the five-year period from 2018 to 2022, the dry and wet seasons were defined based on the visit date for sample collection at the clinical laboratory department. The wet season was represented by visits from June to October, while the dry season was represented by visits from November to May. Based on the visit data, 55% of the full blood count data and 58% of the biochemistry laboratory data were generated in the dry season (Table 1).

Unlike the biochemistry laboratory data, which showed almost equal gender proportions, there was a gender disparity in the haematological laboratory data, with 60% of the routine medical examination full blood count data came from the male population (Table 1).

Table 1: **Summary table of characteristics of sample population**

**Explanatory variables**	**Characteristics**	**Summary N(%)**
**Haematology laboratory**		
Season (N=493)	Dry (n)	271 (55.0)
	Wet (n)	222 (45.0)

Gender (N=493)	Male (n)	300 (60.85)
	Female (n)	193 (39.15)
Dry Season (N=271)	Male (n)	175 (64.58)
	Female (n)	96 (35.42)
Wet Season (N=222)	Male (n)	125 (56.31)
	Female (n)	97 (43.69)
Age	Age range	18-63
	Age (Median)	28.00
	Age (Mean)	30.54
**Biochemistry Laboratory**		
Season (N=643)	Dry (n)	373~~4~~(58.01~~16~~)
	Wet(n)	270~~64~~(41.99~~84~~)
Dry Season (N=373~~4~~)	Male(n)	183~~0~~(49~~8~~.06~~13~~)
	Female (n)	190~~1~~(50~~1~~.94~~87~~)
Wet Season (N=270~~64~~)	Male(n)	148~~39~~(54~~2~~.81~~65~~)
	Female(n)	122(45~~7~~.19~~35~~)
Gender (N= 643)	Male (n)	339~~21~~ (52~~49~~.7~~9~~2)
	Female(n)	304~~16~~(47~~50~~.2~~0~~8)
Age	Age range	18-63
	Age (Median)	33
	Age (Mean)	36.16

Seasonal and Yearly Effects and Reference Intervals

Despite the primary focus was to assess the seasonal effects on the haematological and biochemical parameters, as well as analyse the association between explanatory variables (gender and age) and these parameters (Table 2). Using generalised linear modelling (GLM), we found that seasonality was associated with various haematological indices (HB: 0.014(0.015), P<0.05; RBC: -0.184(0.061), P<0.003; PLTs: 17.47 (6.86), p<0.01; WBC: 0.243(0.163), p = 0.0014; neutrophils: 0.271(0.131), P<0.05; and lymphocytes: -0.003(0.066), P= 0.01). However in the biochemical cohort, the biochemical measurements were not affected by the explanatory variables of dry and wet seasons.

In haematological cohort, age was found to have an association with certain parameters, including haemoglobin (0.028(0.011); P = 0.011), platelets (-0.918(0.44): P = 0.039). In the biochemical cohort, only creatinine (1.202(0.472); P<0.01), and total protein (-0.117(0.048); P<0.05) were found to have an association with age (Table 2). Furthermore, several haematological indices, except for monocytes and eosinophils, were associated with gender. In the biochemical cohort, only sodium (0.680(0.323); P= 0.04) was associated with gender.

We assessed the association of the year of visit on various parameters in healthy individuals (Table 2). Using the GLM approach, we found significant associations between the year of visit and haematological parameters such as haemoglobin (0.156(0.079): P<0.05), RBC (0.080(0.034): P<0.05), platelets (7.861(3.16); P<0.05). In the biochemical cohort, biochemical measurements such as sodium (-0.93(0.31); P<0.05), potassium (-0.107(0.041); P=0.013), albumin (-0.89(0.43); P=0.04), and globulin (1.33(0.50); P=0.01) were found to be associated with the year explanatory variable.

Table 2: Association study of explanatory variables season, age and gender, and all the haematological and biochemical parameters. The association was determined by estimated effects on the median (SE) value of the parameters. P value <0.05; Standard Error of the estimate intercept (SE)

Laboratory parameters	Season[a] Estimate(Std. Error); P value <0.05	Age (Estimate(Std. Error); P value <0.05	Gender[b] (Estimate(Std. Error); P value <0.05	Year (Estimate(Std. Error); P value <0.05
HB (g/dL)	**0.014(0.015); p<0.05**	**0.028(0.011); P =0.011**	**2.355(0.126); P<0.0001**	**0.156(0.079): P<0.05**
RBCa(106/uL)	**-0.184(0.061); p<0.05**	0.004(0.003); P=0.287	**0.750(0.049); P<0.0001**	**0.080(0.034): P<0.05**
PLT (103/uL)	**17.47 (6.86); p<0.01**	**-0.918(0.44): P = 0.039**	**-60.19(6.276); P<0.0001**	**7.861(3.16); P<0.05**
WBC (103/uL)	**0.243(0.163); P<0.01**	-0.006(0.011); P = 0.566	**-0.929(0.158); P<0.0001**	0.030(0.071) P>0.67
Neutrophils (103/uL)	**0.271(0.131); P<0.05**	-0.009(0.008); P=0.295	**-0.624(0.129); P<0.0001**	-0.027(0.05); P=0.61
Lymphocytes (103/uL)	**-0.003(0.066);P< 0.01**	0.006(0.004); P= 0.169	**-0.288(0.0654); P<0.0001**	0.038(0.03); P= 0.22
Monocytes (103/uL)	-0.004(0.165); P=0.798	-0.002(0.001); P=0.092	-0.027(0.017); P = 0.11	**0.037(0.007); P<0.05**
Eosinophils (103/uL)	-0.021(0.023); P=0.359	-0.001(0.001); P=0.380)	0.011(0.023); P= 0.623	-0.01(0.01); P=0.139

Sodium(mmol/L)	-0.238(0.330);P =0.471	0.0188(0.013); P=0.156	**0.680(0.323); P=0.04**	**-0.93(0.31); P<0.05**
GGT (U/L)	0.213(7.31); P=0.977	0.227(0.241); P= 0.347	10.696(7.27); P=0.142	-0.43(1.39); P=0.76
Potassium (mmol/L)	0.020(0.039);P=0.6143	0.001(0.001): P=0.424	0.065(0.039); P=0.098	**-0.11(0.04); P=0.013**
Urea (mmol/L)	-4.128(0.259);P=0.112	0.064(0.040); P=0.113	1.225(1.301); P=0.347	-0.11(0.136); P= 0.42
Creatinine (umol/L)	-18.13(14.88);P=0.224	**1.202(0.472); P<0.01**	28.15(15.23); P=0.066	0.62(2.00); P=0.76
AST (U/L)	-2.045(2.256);P<0.344	-0.066(0.069); P= 0.597	0.566(0.256); P=0.798	-0.56(0.65); P= 0.40
ALT (U/L)	-1.619(2.60); P=0.534	0.031(0.089); P=0.729	1.244(2.846); P=0.662	-1.69(1.12); P=0.14
Albumin (g/dL)	-1.663(0.863); P= 0.06	-0.052(0.029); P=0.08	2.028(0.814); P=0.07	**-0.89(0.43); P=0.04**
Total Protein (g/dL)	-2.142(1.465); P=0.148	**-0.117(0.048=9); P<0.05**	1.017(1.408); P=0.472	-0.51(0.64); P=0.43
AST (U/L)	-0.536(1.228); P=0.663	-0.064(0.041); P= 0.12	-1.057(1.168); 0.367	**1.33(0.50); P=0.01**

[a]Wet Season(reference) is compared to Dry Season; [c]Male (reference) is compared to Female gender;

We determined reference intervals nonparametrically based on the 2.5th and 97.5th percentiles for both haematological indices and biochemical measurements (Table 3). To assess the effects of seasonality, we compared the median values between the dry season and the wet season for each year from 2018 to 2022. Although the differences were not statistically significant across all haematological indices (Wilcoxon test, P>0.05), the median values for platelets, as well as WBC and differentials, were slightly higher in the wet season compared to the dry season. Conversely, the median values for HB and RBC were lower in the wet season. Similarly, the median values for all the biochemical measurements were not statistically significant (Wilcoxon test, P>0.05). However, potassium had a slightly higher median in the dry season (median=4.20) compared to the wet season (median=4.10).

We also evaluated the effects of seasonality on the reference intervals derived from our haematological and biochemical study data. In haematological data, we observed changes in reference intervals from the dry season to the wet season were not significant (Wilcoxon test, P>0.05). However, we observed a slight reduction in the upper limit of reference intervals in the wet season for HB and RBC haematological indices, and a slight increase in the upper limit of WBC and differentials. Similarly, in biochemical measurements, we also found the changes in reference intervals from the dry season to the wet season not statistically significant (Wilcoxon test, P>0.05).

Table 3: The effect of exploratory seasonal factor on the reference ranges of the haematological and biochemical parameters (Wilcoxon test P>0.05).

**Laboratory parameters**	**N**	**Median (Reference Range)**	**N**	**Median (Dry Season)**	**N**	**Wet Season**
HB (g/dL)	461	13.90(11.0-16.5)	258	14.0(11.34-16.36)	206	13.70(10.70-16.29)
RBC[a](10^6/uL)	444	4.87(3.90-5.85)	238	4.95(4.06-5.89)	201	4.77(3.77-5.76)
PLT (10^3/uL)	433	244.5(158.6-346.2)	238	237(156.9-338.2)	197	248(163.5-361.1)
WBC (10^3/uL)	437	5.40(3.52-7.65)	245	5.34(3.51-7.60)	194	5.34(3.52-7.80)
Neutrophils (10^3/uL)	426	2.55(1.34-4.05)	235	2.47(1.32-3.81)	194	2.49(1.40-4.55)
Lymphocytes (10^3/uL)	418	2.15(1.33-2.96)	232	2.14(1.30-3.065)	192	2.14(1.33-2.93)
Monocytes (10^3/uL)	416	0.43(0.25-0.64)	230	0.42(0.25-0.62)	190	0.43(0.25-0.64)
Eosinophils (10^3/uL)	335	0.13(0.13-0.31)	201	0.14(0.02-0.36)	138	0.11(0.01-0.26)
Basophils (10^3/uL)	350	0.04(0.01-0.07)	185	0.03(0.02-0.06)	146	0.04(0.02-0.07)
Sodium (mmol/L)	384	140(136-144)	215	140(137-144)		140(136-144)
Potassium (mmol/L)	498	4.12(3.6-4.7)	235	4.20(3.6-4.7)	152	4.10(3.70-4.50)
GGT (U/L)	240	26(11.00-52.025)	134	26(10.23-51.03)	106	26(12.78-53.00)

Creatinine (umol/L)	500	75.2(50.48-107.05)	289	75(49.00-107.08)	211	77(52.93-106.68)
Urea (mmol/L)	507	3.1(1.7-4.9)	381	3.1(1.80-4.7)	217	3.1(1.70-4.9)
AST (U/L)	354	20(12-32)	211	20(12-34.5)	138	20(12.43-29.00)
ALT (U/L)	234	17(7.0-33.0)	130	17(6.23-33.78)	104	17(8.15-31.43)
Albumin (g/dL)	236	40(34-47)	150	40(34.00-47.00)	82	40(34.03-45.00)
Total Protein (g/dL)	336	76(67.00-83.63)	195	76(68.00-85.00)	147	76(67.00-83.35)
Globulin (g/dL)	203	35(27.0-42.00)	116	35(27-42.13)	73	35(29.8-40.0)

[a]Mean value was used for RBC parameter because the data was normally distributed

Monthly and Yearly Pattern in Parameters

We further analysed patterns based on changes in the monthly and yearly median of haematological and biochemical parameters (Fig 2 and S1-S2 Fig). These figures demonstrated a change in the pattern of our monthly median of haematological indices and biochemical measurements from January to December over five year period. From these our study results (Fig 2, S1-S2 Fig), we observed that laboratory parameters, including HB, RBC, HCT/PCV showed seasonal patterns throughout the year, with levels being normal to slightly low during the dry season and normal to high during the wet season. Likewise we observed a seasonal pattern for total WBC, neutrophils, lymphocytes, and platelets from November to March with normal to slightly high median . In terms of biochemistry parameters, liver enzymes (AST, ALT, GGT, total protein) and potassium were generally high in the wet season (June to October) but moderately low in the dry season.

Finally, we assessed the changes in median values of the parameters from 2018 to 2022 using the boxplots in Fig 3 and 4. We followed this with a statistical significance test using the non-parametric Kruskal-Wallis test. Overall, no statistically significant difference was found (Kruskal-Wallis chi-squared = 0.1148, p-value = 1) among the median values of all the haematological indices and biochemical measurements in the last five years (2018 to 2022). In haematological data, the median values for HB and RBC parameters decreased in 2020, while the median values for total WBC count, absolute neutrophils count, and platelets increased slightly. As for biochemical parameters, there was no clear pattern except for AST and ALT, which showed a downward trend from 2018 to 2022.

Fig 2: Monthly pattern of haematological and biochemical parameters from 1st January 2018 to 31st December 2022 in five year period. Haemoglobin (HB); Red blood cells (RBC); White blood cells (WBC); Platelets (PLT); Neutrophils (NEUTRO); Lymphocytes (LYMPH); Mean cell volume (MCV); Packed cell volume (PCV); Eosinophils (EOS); Basophil (BASO); Aspartate aminotransferase (AST); Alanine transaminase (ALT); Gamma-glutamyl transferase (GGT).

Fig 3: Boxplot showing pattern/trend of haematological parameters in the last five years. The results displayed in medians (Kruskal-Wallis chi-squared = 0.063, p-value = 1).

Fig 4: Boxplot showing pattern/trend of biochemical parameters in the last five years. Aspartate aminotransferase (AST); Alanine transaminase (ALT); Gamma-glutamyl transferase (GGT). (Kruskal-Wallis chi-squared = 0.8433, p-value = 0.93)

Discussion

The study examined the effects of seasonality on haematological indices and biochemical measurements, as well as the derived reference intervals. Seasonal variations can be influenced by factors such as changes in environmental temperature and humidity, diet, physical activity, and seasonality of certain diseases like malaria. Overall, no statistically significant differences were found between the median values generated in the dry and wet seasons as well as reference intervals generated between the dry and wet season, indicating that there was no seasonal effect on the reference intervals derived from our study data. The reference intervals derived from our study data were comparable to the results reported by Adetifa et al in 2009, which focused on Gambian adults without any known underlying health conditions during a TB contact tracing study (38). Notably, there was a slight difference in the upper limit of neutrophils reference intervals between the dry season (1.32-3.81) and wet season (1.40-4.55), suggesting the need for more informative reference intervals that consider seasonality. These seasonal variations should be taken into consideration, especially in research studies or clinical trials where patients are monitored at different times of the year. Being aware of these subtle seasonal variations can help prevent erroneous conclusions based on study data.

To further investigate the seasonal variation pattern of these parameters from January to December, we observed moderately normal to slightly higher median pattern for haemoglobin and RBC during the wet season. Haemoglobin and RBC parameters are crucial for diagnosing anaemia in clinical settings. It has been reported that haemoglobin levels in individuals without any known underlying health conditions can vary due to seasonal factors such as changes in diet, including increased consumption of iron-rich foods during certain seasons, as well as asymptomatic infection (22). In the Gambia, the wet season is

characterized by the abundant availability of iron-rich foods, which could explain the higher levels of haemoglobin and RBCs during this period (21). Besides, the moderately normal to high HCT seasonal variation patterns in the wet season also demonstrate the high haemoglobin and RBC medians in the wet season could be attributed to low fluid intake during the wet season which accompanies low temperature and humidity (21).

The seasonal variations in WBC and platelets were observed in our study from November to March. In the Gambia, changes in the environment during the wet season, such as increased rainfall and cold weather from November to March, favour the transmission of malaria parasites, which typically peaks in November, as well as respiratory infections (24–29). Our study supports previous findings regarding the seasonal effects on WBC and differentials (1). Infectious diseases like malaria tend to flourish during the wet season, likely due to environmental factors such as heavy rainfall leading to stagnant water and waterlogged areas (30-33). This can potentially lead to an increase in mean WBC count and differentials in the general population due to low-grade inflammation in the community caused by malaria transmission and viral illnesses (3,10). The seasonal variation in WBC and differentials could be impacted by malaria parasite infection. However, our study records showed malaria microscopy was negative for both haematological cohort and biochemical cohort data retrieved electronically.

Similar to hemoglobin and RBCs, the monthly pattern of liver enzymes and potassium changed based on seasonality. Our findings are aligned with earlier studies that have demonstrated how seasonal changes in diet and fluid intake can impact biochemical measurements, especially electrolytes. The seasonal effects on these biochemical measurements may be attributed to the temperature difference between the wet and dry seasons (34–36). A reference interval study conducted in Guinea also reported seasonal

effects on biochemical parameters, particularly liver enzymes (31). Despite reports of dehydration being more common during the dry season and potentially affecting biochemical measurements like serum creatinine and urea levels, our study found that median values for urea and creatinine remained relatively normal across both seasons (37).

The first case of COVID-19 in the Gambia was identified on March 17, 2020, which eventually led to a government-mandated lockdown and restrictions in an effort to curtail community transmission (40). While there have been no significant differences in the median values for all parameters during the past five years, the decrease in median values for haemoglobin and RBCs in 2020, along with the increase in median value for total WBC, might be attributed to the effects of the COVID-19 pandemic on lifestyles. A study conducted by Werf et al. in 2021 supports this, as it reported that the COVID-19 pandemic has had an impact on lifestyles in the Netherlands(39). This impact includes limited access to healthy diets and fresh fruits and vegetables, changes in eating patterns, and reduced physical activity which could have an impact on the overall health of the public. Hence, further qualitative study is needed to shed light on the impact of COVID-19 pandemic on life styles in the Gambia. Alarmingly, our study also revealed a consistent decline in the median values of AST and ALT, warranting further investigation. Public health authorities should closely monitor the trends in haematological indices and biochemical measurements to assess the lasting impact of the pandemic on the overall public health.

As observed in numerous retrospective studies, our study is also subject to limitations that restrict our control over certain factors. These factors include haemoglobin genotype status, body size, smoking, alcohol consumption, exercise, and contraceptive pill use, all of which have the potential to influence the outcome (17). Additionally, our sample size was constrained by the available data, and we were unable to obtain additional data to enhance

statistical power (18,19). These limitations could be addressed through the implementation of a well-designed prospective study conducted on a general population, following the approach employed by Okebe et al. in 2016. Despite these limitations, we maintain that the results of our study are accurate, as our sample size was considerable. Therefore, our study remains representative and supports the current prospective reference intervals study that will provide a more comprehensive sampling and data stratification reflecting both dry and wet seasons.

Conclusion

Our study found no significant seasonal effects on the reference intervals for haematological and biochemical parameters, despite the identification of certain parameters that exhibited some association with seasonality. However, we did observe seasonal variations in specific parameters such as haemoglobin, red blood cells, liver enzymes, and potassium levels from January to December. Furthermore, we did not observe significant yearly variations in the median values of the all haematological indices and biochemical measurements. Therefore, our findings emphasize the importance of considering seasonal variations in haematological indices and biochemical measurements when interpreting laboratory results and making diagnostic decisions, as well as when recruiting participants for clinical trial projects. Our study should be followed by well-designed context-specific studies that employ standardized sampling and analytical methods within our geographical setting.

Acknowledgements

Not Applicable

Availability of Data and Materials

The datasets used and/or analysed during the current study are available and submitted with manuscript

Financial Disclosure Statement

This project received no specific funding for this work.

Funding Information

The study was funded by Medical Research Council the Gambia at London School of Hygiene and Tropical Medicine

Consent for publication: Not applicable

Competing Interest

The authors have declared that no competing interests exist.

I declare that the authors have no competing interests as defined by BMC, or other interests that might be perceived to influence the results and/or discussion reported in this paper.

Formatted: Font: Bold

Formatted: Font: Bold

Abbreviations

Formatted: Font: Bold

COVID-19: Coronavirus Disease 2019

LSHTM: London School of Hygiene and Tropical Medicine

MRCG@LSHTM: Medical Research Council Unit The Gambia at LSHTM

HIV: Human Immunodeficiency Virus

GGT: Gamma-glutamyl transpeptidase

AST: Aspartate aminotransferase

ALT: Alanine Aminotransferase

TP: Total Protein

ALB: Albumin

WBC: White Blood Cells

RBC: Red Blood Cells

HB: Haemoglobin,

PLT: Platelets

GLM: Generalised Linear Modelling

Author’s Contributions

MD and AAN designed the study and drafted the manuscript. MD, MDD, FS, RB, NS, MB, and SU performed the laboratory analysis, data retrieval and performed data arrangements. MD and AAN performed the statistical analysis. All authors reviewed and approved the final manuscript.

References

Formatted: Font: Bold

1. Bæk O, Jensen KJ, Andersen A, Balé C, Martins C, Biering-Sørensen S, et al. Seasonal and sex-specific variations in haematological parameters in 4 to 5.5-month-old infants in Guinea-Bissau, West Africa. Trans R Soc Trop Med Hyg. 2017;111(1).

2. Béavogui AH, Touré AA, Camara D, Doumbouya A, Sylla MM, Touré S, et al. Haematological and Biochemical Reference Values for Healthy Population of Maferinyah Rural Community in Guinea. Biomed Res Int. 2020;2020.

3. Okebe J, Mwesigwa J, Agbla SC, Sanya-Isijola F, Abubakar I, D'Alessandro U, et al. Seasonal variation in haematological and biochemical reference values for healthy young children in The Gambia. BMC Pediatr. 2016;16(1).

4. Gidlow DA, Church JF, Clayton BE. Seasonal Variations in Haematological and Biochemical Parameters. Ann Clin Biochem. 1986;23(3).

5. Marriott BM. Nutritional Needs in Hot Environments: Applications for military personnel in Field Operations. Nutritional Needs in Hot Environments. 1993.

6. Chan CB, Ryan DA. Assessing the effects of weather conditions on physical activity participation using objective measures. Vol. 6, International Journal of Environmental Research and Public Health. 2009.

7. Pasiakos SM. Nutritional Requirements for Sustaining Health and Performance during Exposure to Extreme Environments. Vol. 40, Annual Review of Nutrition. 2020.

8. Turrisi TB, Bittel KM, West AB, Hojjatinia S, Hojjatinia S, Mama SK, et al. Seasons, weather, and device-measured movement behaviors: a scoping review from 2006 to 2020. Vol. 18, International Journal of Behavioral Nutrition and Physical Activity. 2021.

9. Akinbo FO, Okaka CE, Omoregie R. Seasonal Variation of Intestinal Parasitic Infections Among HIV-Positive Patients in Benin City, Nigeria. Ethiopian journal of health sciences. 2011;21(3).

10. Mwesigwa J, Okebe J, Affara M, Di Tanna GL, Nwakanma D, Janha O, et al. On-going malaria transmission in The Gambia despite high coverage of control interventions: A nationwide cross-sectional survey. Malaria Journal. 2015;14(1).

11. Collins KA, Ceesay S, Drammeh S, Jaiteh FK, Guery MA, Lanke K, et al. A Cohort Study on the Duration of Plasmodium falciparum Infections During the Dry Season in The Gambia. Journal of Infectious Diseases. 2022;226(1).

12. Serena V, Alessandro M, Maurizio, Nadia T, Valeria S, Eleonora C, et al. Baseline haematological and biochemical reference values for healthy male adults from mali. Pan African Medical Journal. 2019;32.

13. Ruzagira E, Abaasa A, Karita E, Mulenga J, Kilembe W, Allen S, et al. Effect of seasonal variation on adult clinical laboratory parameters in Rwanda, Zambia, and Uganda: Implications for HIV biomedical prevention trials. PLoS ONE. 2014;9(8).

14. Ezzelle J, Rodriguez-Chavez IR, Darden JM, Stirewalt M, Kunwar N, Hitchcock R, et al. Guidelines on good clinical laboratory practice: Bridging operations between research and clinical research laboratories. Vol. 46, Journal of Pharmaceutical and Biomedical Analysis. 2008.

15. Joshi RK, Sarangi SC, Mohapatra S, Mallick S, Datta SK. A Comparative Review of ICMR, WHO, and EMA Guidelines for Good Clinical Laboratory Practices. Journal of Laboratory Physicians. 2023;15(02).

16. International Organization for standardization. ISO 15189:2012 - Medical laboratories-Requirements for quality and competence. International Organization for standardization ISO. 2012;2012.

17. Talari K, Goyal M. Retrospective studies - Utility and caveats. Journal of the Royal College of Physicians of Edinburgh. 2020;50(4).

18. Fitzner K, Heckinger E. Sample size calculation and power analysis: a quick review. Diabetes Educ. 2010;36(5):701-7. doi: 10.1177/0145721710380791.~~Suresh K, Chandrashekara S. Sample size estimation and power analysis for clinical research studies. Vol. 5, Journal of Human Reproductive Sciences. 2012~~.

19. Kim J, Seo BS. How to calculate sample size and why. Clinics in Orthopedic Surgery. 2013;5(3).

20. Critchley J, Bates I. Haemoglobin colour scale for anaemia diagnosis where there is no laboratory: A systematic review. International Journal of Epidemiology. 2005;34(6).

21. World Health Organization. Countdown to 2015 decade report (2000-2010) with country profiles: taking stock of maternal, newborn and child survival. Maternal, Newborn & Child Survival Countdown to 2015. 2015;

22. Semagn BE, Gebreegziabher ZA, Abebaw WA. Iron-rich food consumption and associated factors among children aged 6–23 months in Sierra Leone: multi-level logistic regression analysis. BMC Public Health. 2023;23(1).

23. Ayanful-Torgby R, Quashie NB, Boampong JN, Williamson KC, Amoah LE. Seasonal variations in Plasmodium falciparum parasite prevalence assessed by varying diagnostic tests in asymptomatic children in southern Ghana. PLoS ONE. 2018;13(6).

24. Chowdhury FR, Ibrahim QSU, Shafiqul Bari M, Jahangir Alam MM, Dunachie SJ, Rodriguez-Morales AJ, et al. The association between temperature, rainfall and humidity with common climate-sensitive infectious diseases in Bangladesh. PLoS ONE. 2018;13(6).

25. Prentice AM, Mendoza YA, Pereira D, Cerami C, Wegmuller R, Constable A, et al. Dietary strategies for improving iron status: Balancing safety and efficacy. Nutrition Reviews. 2017;75(1).

26. Maes M, Stevens W, Scharpe S, Bosmans E, De Meyer F, D'Hondt P, et al. Seasonal variation in peripheral blood leukocyte subsets and in serum interleukin-6, and soluble interleukin-2 and-6 receptor concentrations in normal volunteers. Experientia. 1994;50(9).

27. Ruul A, Saar B. Anaemia of chronic disease. Eesti Arst. 2015;94(9).

28. Kurkina N V., Gorshenina EI, Chegodaeva L V., Polagimova A V. Anemia of Chronic Diseases. Klinicheskaya Onkogematologiya/Clinical Oncohematology. 2021;14(3).

29. Fraenkel PG. Understanding anemia of chronic disease. Hematology (United States). 2015;2015(1).

30. Fares A. Factors influencing the seasonal patterns of infectious diseases. International Journal of Preventive Medicine. 2013;4(2).

31. Abdoul Habib Béavogui, Almamy Amara Touré, Daouda Camara, Abdoulaye Doumbouya, Malick Minkael Sylla, Sékou Touré, Ahmadou Hamidou Togo, Mamadou Saliou Diallo, Alexandre Delamou, Issaka Sagara, Alassane Dicko, Abdoulaye Djimdé, "Haematological and Biochemical Reference Values for Healthy Population of Maferinyah Rural Community in Guinea", BioMed Research International. 2020;2020. https://doi.org/10.1155/2020/8605485

32. Mandala W, Munyenyembe A, Sulani I, Soko M, Mallewa J, Hiestand J. Acute Malaria in Malawian Children and Adults is Characterized by Thrombocytopenia That Normalizes in Convalescence. Journal of Blood Medicine. 2022;13.

33. Bhatia I, Sharma A, Guleria S, Sharma A, Jaswal RS, Vaid A, et al. Thrombocytopenia in Children: A Large Prospective Study on Clinical Manifestations, Seasonal Variation, Etiology, and Outcome. Journal of Association of Physicians of India. 2023;71(3).

34. Kailakov AM. Seasonal fluctuations in the content of some electrolytes in patients with diabetes mellitus. Problemy Endokrinologii. 1979;25(2).

35. Swingler G, Power D. Seasonal plasma electrolyte fluctuations in childhood diarrhoea. Archives of Disease in Childhood. 2002;87(5).

36. Koyama T, Makinouchi R, Machida S, Matsui K, Shibagaki Y, Imai N. Seasonal Changes in the Prevalence of Hyperkalemia in the Emergency Department: A Single Center Study. Medicina (Lithuania). 2022;58(2).

37. Ephraim RKD, Asamoah CA, Abaka-Yawson A, Kwadzokpui PK, Adusei S. Climate change causes changes in biochemical markers of kidney disease. BMC Nephrology. 2020;21(1).

38. Adetifa IM, Hill PC, Jeffries DJ, Jackson-Sillah D, Ibanga HB, Bah G, Donkor S, Corrah T, Adegbola RA. Haematological values from a Gambian cohort--possible reference range for a West African population. Int J Lab Hematol. 2009 Dec;31(6):615-22. doi: 10.1111/j.1751-553X.2008.01087.x. Epub 2008 Jul 10. PMID: 18631172.

39. van der Werf, E.T., Busch, M., Jong, M.C. et al. Lifestyle changes during the first wave of the COVID-19 pandemic: a cross-sectional survey in the Netherlands. BMC Public Health 21, 1226 (2021). https://doi.org/10.1186/s12889-021-11264-z

40. Abatan B, Agboghoroma O, Akemoke F, Antonio M, Awokola B, Bittaye M, Bojang A, Bojang K, Brotherton H, Cerami C, Clarke E, D'Alessandro U, de Silva T, Drammeh M, Forrest K, Hofmann N, Jagne S, Jah H, Jarju S, Jaye A, Jobe M, Kampmann B, Manjang B, Martinez-Alvarez M, Mohammed N, Nadjm B, Ndiath MO, Nkereuwem E, Nwakanma D, Oko F, Okoh E, Okomo U, Olatunji Y, Oriero E, Prentice AM, Roberts C, Roca A, Sabally B, Sambou S, Samateh A, Secka O, Sesay AK, Singhateh Y, Susso B, Usuf E, Vilane A, Wariri O. Intense and Mild First Epidemic Wave of Coronavirus Disease, The Gambia. Emerg Infect Dis. 2021; 27(8):2064-2072. doi: 10.3201/eid2708.204954.

Supporting Information Files

Formatted: Font: Bold

S1 File: Study Raw Data

S2 File: L2023.E03_Dibbasey_Ethics_Waiver_1

S1 Fig: Haematological indices from January to December in five year period. The point showed monthly medians in five year period.

S2 Fig: Biochemical measurements from January to December in five year period. The point showed monthly medians in five year period.

Authors' Responses to Editor's and Reviewers Comments

Ref: Submission ID: PGPH-D-23-02498

Title: Re: " **Seasonal and yearly variations in haematological and biochemical parameters of healthy Gambian adults: a retrospective study"**

Dear Editor and Reviewers,

First and foremost, I would like to thank you all for the thorough review process, scientifically thoughtful comments and suggestions which will hopefully improve the manuscript quality and its scientific rigor. We have endeavored to provide point-by-point response to the comments and undertake any typographical and phrasing errors correction. Further, we have re-written sentences to eliminate any ambiguity in our manuscript and removed unwanted text.

Reviewer #1

Reviewer's General Comment

General comment: The use of the term/word healthy people is problematic. If someone came to the laboratory for routine check-up/testing does not make such individual healthy person. Perhaps, individual with no known underlying health condition could suffice. Although the study design was retrospective, reading through the entire write-up appears more prospective.

Author's Response: This is addressed as we have replaced the term "healthy individual" with "individual with no known underlying condition". Also, we have updated the manuscript to reflect more retrospective study design. For instance, we have deleted the entire section on "laboratory equipment and method section".

Reviewer's Comment on Methods

Reviewer's Comment 1: Line 90 – 93 is not clear. Please revise

Author's Response: Revised to make it more clearer. The entire paragraph was also revised to ensure clarity.

Reviewer's Comment 2: Line 96, we are being encouraged not to start sentences with abbreviation

Author's Response: This is corrected

Reviewer's Comment 3: Line 104 -108, The definition of healthy people is challenging

Author's Response: This is addressed as we have replaced the term "healthy individual" with "individual with no known underlying condition". Based on the medical records, all those that have

positive malaria, HIV and hepatitis were excluded from the final dataset. We also disregarded all those with known medical condition such as Diabetes and hypertension during data retrieval stage.

Reviewer's Comment 4: Line 108, statement suggest more prospective design

Author's Response: This statement was corrected. The updated one is "The extracted biochemical data comprised serum Gamma-glutamyl transpeptidase (GGT), Sodium, Potassium, Urea, Creatinine, Aspartate aminotransferase (AST), Alanine Aminotransferase (ALT), Total Protein (TP), Albumin (ALB), and Globulin results"

Reviewer's Comment 5: Line 113 – 114, it is not clear whether the study included persons aged 18 – 63 or exempted these age group rather

Author's Response: It is true that there was no clarity. However, we have addressed this and updated the entire paragraph. The updated paragraph "During the data extraction, we considered the age range of 18-63 years, in accordance with the employment age range outlined in MRCG@LSHTM policies. As such, those records that fall outside of 18 to 63 age range were excluded from the final data for analysis (figure 1). We excluded data from the study if the electronic medical records showed positive serological results for Human Immunodeficiency Virus (HIV), and Hepatitis B".

Reviewer's Comment 6: Line 117 (the figure 1), is confusing. What is Hep? What is the meaning of Age (18-63) = 129? The entire figure 1 is not clear.

Author's Response: It is true that figure 1 lack clarity which I have addressed and explained the details of the figure in the method section. We have added extra information in the figure legend to add bit more clarity. Hep is shorten version of Hepatitis B but this abbreviation was updated to Hepatitis B.

Reviewer's Comment 7: Line 120 – 130, this section appears the authors did the sampling themselves

Author's Response: We have deleted the entire section on "laboratory equipment and method section" as it those reflect more of prospective study design than retrospective study design. Hence, the entire paragraph is deleted.

Reviewer's Comment 8: Line 157 is contradicting with line 103

Author's Response: Yes it is true that I didn't clearly explain how we came to the final dataset of 493. However, this is clearly addressed in the method section now as initial dataset underwent some data cleaning whereby 10 records were found to be either HIV or Hepatitis B positive or fall outside the specified age range targeted for this study (i.e. 18 to 63 years). Those records that fall outside this age range were excluded. Hence this gave us final dataset of 493 for haematological full blood count

data and 643 for biochemical data. The sentence added to figure 1 legend is “The excluded records were from individuals whose age fall outside our specified age range of 18 to 63 years or the records that showed HIV/Hepatitis B positive, and/or Malaria positive”.

Reviewer’s Comment on Discussion

Reviewer’s Comment 1: It is not clear why the authors are comparing their study to a previous work among children less then 5 years (line 264 -266).

Author’s Response: The reason I compared my finding to the finding of Okebe et al. is because of the study was conducted in similar geographical setting despite difference in study population. In this study, they investigated the effect of seasonality on reference range developed in wet and dry season in children 0-5yrs. However, the comparison does not have a solid scientific merit, hence I decided to removed it.

Reviewer’s Comment 2: It is also not clear if the study evaluated the malaria or other parasitic infectious state of the study participants. It can be argued that, during the raining season, parasitic infection is high and can affect the haematological parameters. Therefore, the observed variation in the parameters require clear explanation.

Author’s Response: The study excluded not only those records that were found to have positive HIV and/or hepatitis B results but malaria positive results were also excluded. However, we did not have any positive malaria case as indicated in figure 1 and the raw data. We have updated our exclusion criteria section in our method section which has addressed this point.

Reviewer’s Comment 3: Why was hematocrit (HCT) not included to substantiated the hemoglobin (Hb) recorded. It is possible for people to take less fluid during the raining season and get dehydrated.

Author’s Response:

Seasonality is characterised by changes in temperature, humidity, rainfall, food system etc. Dehydration due to less water intake or extreme heat is also included. Hence, the primary objective is to assess the overall impact of seasonality on haematological and biochemical parameters. As such, I do not think looking at HCT is needed to substantiate the HB recorded. Ideally, we should have looked at HCT in isolation to assess the association of season explanatory variable and HCT but we focused on haematological indices directly measured instead of calculated indices by automated haematology analyser platform in our laboratory which include HB, RBC, WBC and differentials, and PLT. That’s the reason we disregarded HCT in the association study.

However, the haematocrit (HCT), which represented in the heatmap as packed cell volume (PCV), was included in the analysis for seasonal variation pattern study in figure 2 which shed light on the seasonal/monthly changes pattern of both haematological indices and biochemical measurements. The

heatmap showed HCT/PCV has seasonal variation pattern in line with similar pattern observed with HB and RBC. This is further discussed in our discussion section to add more context to haemoglobin and red blood cells seasonal variation pattern results.

Reviewer #2:

Reviewer's Comment on Title and Abstract

Reviewer's Comment: Title:-if possible; the title can be changed by remove of the word (yearly), and addition of the year to (retrospective study 2018-2022)

Author's Response: Addressed

Reviewer Comment on Abstract:

Reviewer's Comment 1-Divide background from the aim of the study

Reviewer's Comment 2-Better to write the objective of the study.

Author's Response: Yes we have addressed both comment 1 and 2. We started the abstract with objective of the study.

Reviewer's Comment 3-in abstract give brief idea about study design, study population and area, sampling, methods of data collection and the measurements (not more than 5 rows).

Author's Response: Addressed

Reviewer's Comment 4: briefly give the main results of the study with the accurate numbers.

Author's Response: I realised some key details regarding my study was missing in the abstract. For instance, the sentence **"The median values for HB and RBC as well as liver enzymes and potassium were high in the wet season but low in dry season**" was ambiguous as we were summarising the heatmap results from our data as we showed some seasonal patterns of some laboratory parameters. This is now explained clearly in this sentence "**In addition, we observed in our heatmap result that some laboratory parameters, including HB, RBC, HCT/PCV, urea, liver enzymes, and potassium, showed seasonal patterns throughout the year, with levels being normal to slightly low during the dry season and normal to high during the wet season".**

Reviewer's Comment 5-there is some confusion regarding the effect of seasonal differences in the measurement of haematological indices and biochemical measurements.

Author's Response: The study showed an association but when we further analysed the effects of seasonality after eliminating all the outliers, we found no seasonal effects as the medians from the wet season and dry season of all the haematological indices and biochemical measurements were not statistically significantly different using Wilcox test. However, what we found in this study was that we observed a seasonal patterns of haematological indices including HB, RBC, and HCT/PCV, and biochemical measurements such as liver enzymes and potassium.

Under the results section, we have re-written the results section to add more clarity to our data and eliminate any ambiguity about the results. In the result section, here is the re-written sentences "**Our study found a positive association between seasonality (wet season as the reference) and HB (HB: 0.014(0.015), P<0.05), WBC (WBC: 0.243(0.163), p =0.0014), and neutrophils (neutrophils: 0.271(0.131), P<0.05) with exception to RBC (RBC: - 0.184(0.061), P< 0.003) that showed negative association. Despite the association, the seasonal effects on our derived reference intervals from the wet season to the dry season were not statistically significant (Wilcoxon test, P>0.05). In addition, we observed in our heatmap result that some laboratory parameters, including HB, RBC, HCT/PCV, urea, liver enzymes, and potassium, showed seasonal patterns throughout the year, with median levels being normal to slightly low during the dry season and normal to high during the wet season".**

Comments 6-write only the conclusion of the result. interpretation of result can be discussed in the section of discussion.
-use mean full and brief key words

Author's Comment: All these points are valid and were addressed.

Reviewer's Comments on Methods:
- some texts were not needed
- regarding exclusion and inclusion criteria, what about the other seasonal infections e.g. influenza
- what is the final diagnose of the healthy participants who participate in the current study.
- the biomedical history of the participants is absent from the current study.
- inclusion and exclusion criteria should minimize the un-suspected results or errors.

Authors Response: All these points were addressed. The complete biomedical history of patients data is not available but we have certain biomedical data such HIV, Hepatitis B, and malaria status found in raw data retrieved from the electronic database. Based on the records available, the patients with no known underlying condition, HIV and hepatitis B negative and malaria microscopy negative were included in the final dataset as shown in figure 1. However, the seasonal infections status such as influenza data was not available in their clinical biomedical data. The clinical data of the patients usually indicate any underlying condition, hence all those with underlying condition visited the lab for routine medical examination were not retrieved. Hence we focused on those sent for Routine Medical Examination/Assessment without any known underlying condition as indicated in their clinical records.

Reviewer's Comment on Results:

-493 participant for haematological indexes.

-643 participant for biochemical measurements.

-the measured parameters was for separated individuals?

-643 and 493 individuals measured for different parameters.

-the current study have 2 study population.

-the study must divide into two studies.

In the results section its clearly that there was 2 study population:

-in the first one the researcher examine the seasonal haematological changes.

-in the second one the researcher examine the biochemical changes.

Then:

-there was a comparisons between the two populations.

So:

-the researcher must find the haematological indexes and biochemical measurement change in one population.

OR:

-compare the change in hematological indexes in the first population which occurs as a result of seasonal change as separate work.

-then find the seasonal changes in biochemical measurements in the second population

-finally the researcher can compare the results of the two studies

Author's Response: Dear reviewer, I don't know whether I fully comprehend your comments on separating the haematological cohort and biochemical cohort and then compare the findings. We have two separate dataset for haematological indices (493) and biochemical measurements (643)

and their datasets were analysed independently because they were retrieved from two different departments in our lab. In the results section, we try to summarised the findings based on the context of the section explanation. To address your points, we have added further explanation and made some adjustments to reflect the two study populations/data, haematological indices data and biochemical measurements data.

Reviewer's Comment on Discussion:

Reviewer's Comment 1-in discussion there is unacceptable justification regarding the personal, socio- demographic and life style data which didn't appear in the methods of data collection in form of questionnaire or study objectives.

Author's Response: Dear Reviewer, it is true that I mentioned that the COVID-19 pandemic impacted lifestyles. However, I used previously published peer-reviewed publication from Werf et al. 2021 to backed the statement that pandemic has impacted the lifestyles across many countries. I have also adjusted the paragraph to add more clarity .

Thank you reviewer's once again for this thoroughly resounding review. The comments were objectively laid out and I have endeavoured to address them objectively as well. Based, on these addressing these comments, I have observed that manuscript has being improved.

Mustapha Dibbasey
PhD Student
University of Ghana | London School of Hygiene and Tropical Med

Authors' Responses to Editor's and Reviewers Comments

Ref: Submission ID: PGPH-D-23-02498

Title: Re: " **Seasonal and yearly variations in haematological and biochemical parameters of healthy Gambian adults: a retrospective study 2018-2022"**

Dear Editor and Reviewers,

First and foremost, I would like to express my gratitude for the comprehensive review process, scientifically thoughtful comments, and suggestions. These inputs will undoubtedly contribute to the enhancement of the manuscript's quality and scientific rigour. We have made a concerted effort to provide a point-by-point response to the comments and correct any typographical and phrasing errors. Additionally, we have rephrased sentences to eliminate ambiguity and removed unnecessary text.

Editor comments

1. Please review your reference list to ensure that it is complete and correct. If you have cited papers that have been retracted, please include the rationale for doing so in the manuscript text, or remove these references and replace them with relevant current references. Any changes to the reference list should be mentioned in the rebuttal letter that accompanies your revised manuscript. If you need to cite a retracted article, indicate the article's retracted status in the References list and also include a citation and full reference for the retraction notice.

Response to Editor:

Thank you for bringing to our attention that the paper by Suresh K and Chandrashekara S titled "Sample size estimation and power analysis for clinical research studies," published in the Journal of Human Reproductive Sciences in 2012, has been retracted. Referenced as **"Suresh K, Chandrashekara S. Sample size estimation and power analysis for clinical research studies. Vol. 5, Journal of Human Reproductive Sciences. 2012".** We have replaced this retracted paper with the article by Fitzner and Heckinger from 2010, titled "Sample size calculation and power analysis: a quick review," published in Diabetes Educ. The reference is as follows: **Fitzner K, Heckinger E. Sample size calculation and power analysis: a quick review. Diabetes Educ. 2010;36(5):701-7. doi: 10.1177/0145721710380791.**

Reviewer #2

Response to Reviewer #2 Comment on data analysis in Table 1

Once again, I would like to thank reviewer #2 for the thorough review and for identifying the calculation error in Table 1. This was an oversight on our part. After re-examining the code used in R, we found that it was incorrectly laid out, resulting in these erroneous results. We have rectified the error in Table 1.

Yours Sincerely

Mustapha Dibbasey

PhD Student | University of Ghana | London School of Hygiene and Tropical Med
