# Supplementary material for: Seasonal variations in haematological and biochemical parameters of healthy Gambian adults: Retrospective study 2018–2022
Source: PLOS Glob Public Health. 2024 Sep 17;4(9):e0003715. doi: 10.1371/journal.pgph.0003715 (PMC11407651; doi:10.1371/journal.pgph.0003715)
Supplement: Attachment — Submitted filename: Authors Response Letter.docx [file pgph.0003715.s007.docx]

**Authors’ Responses to Editor’s and Reviewers Comments**

**Ref: Submission ID: PGPH-D-23-02498**

Title: Re: " **Seasonal and yearly variations in haematological and biochemical parameters of healthy Gambian adults: a retrospective study”**

**Dear Editor and Reviewers,**

First and foremost, I would like to thank you all for the thorough review process, scientifically thoughtful comments and suggestions which will hopefully improve the manuscript quality and its scientific rigor. We have endeavored to provide point-by-point response to the comments and undertake any typographical and phrasing errors correction. Further, we have re-written sentences to eliminate any ambiguity in our manuscript and removed unwanted text.

**Reviewer #1**

**Reviewer’s General Comment**

**General comment:** The use of the term/word healthy people is problematic. If someone came to the laboratory for routine check-up/testing does not make such individual healthy person. Perhaps, individual with no known underlying health condition could suffice. Although the study design was retrospective, reading through the entire write-up appears more prospective.

**Author’s Response:** This is addressed as we have replaced the term “healthy individual” with “individual with no known underlying condition”. Also, we have updated the manuscript to reflect more retrospective study design. For instance, we have deleted the entire section on “laboratory equipment and method section”.

**Reviewer’s Comment on Methods**

**Reviewer’s Comment 1:** Line 90 – 93 is not clear. Please revise

**Author’s Response:** Revised to make it more clearer. The entire paragraph was also revised to ensure clarity.

**Reviewer’s Comment 2:** Line 96, we are being encouraged not to start sentences with abbreviation

**Author’s Response:** This is corrected

**Reviewer’s Comment 3:** Line 104 -108, The definition of healthy people is challenging

**Author’s Response:** This is addressed as we have replaced the term “healthy individual” with “individual with no known underlying condition”. Based on the medical records, all those that have positive malaria, HIV and hepatitis were excluded from the final dataset. We also disregarded all those with known medical condition such as Diabetes and hypertension during data retrieval stage.

**Reviewer’s Comment 4:** Line 108, statement suggest more prospective design

**Author’s Response:** This statement was corrected. The updated one is “The extracted biochemical data comprised serum Gamma-glutamyl transpeptidase (GGT), Sodium, Potassium, Urea, Creatinine, Aspartate aminotransferase (AST), Alanine Aminotransferase (ALT), Total Protein (TP), Albumin (ALB), and Globulin results”

**Reviewer’s Comment 5:** Line 113 – 114, it is not clear whether the study included persons aged 18 – 63 or exempted these age group rather

**Author’s Response:** It is true that there was no clarity. However, we have addressed this and updated the entire paragraph. The updated paragraph “During the data extraction, we considered the age range of 18-63 years, in accordance with the employment age range outlined in MRCG@LSHTM policies. As such, those records that fall outside of 18 to 63 age range were excluded from the final data for analysis (figure 1). We excluded data from the study if the electronic medical records showed positive serological results for Human Immunodeficiency Virus (HIV), and Hepatitis B”.

**Reviewer’s Comment 6:** Line 117 (the figure 1), is confusing. What is Hep? What is the meaning of Age (18-63) = 129? The entire figure 1 is not clear.

**Author’s Response:** It is true that figure 1 lack clarity which I have addressed and explained the details of the figure in the method section. We have added extra information in the figure legend to add bit more clarity. Hep is shorten version of Hepatitis B but this abbreviation was updated to Hepatitis B.

**Reviewer’s Comment 7:** Line 120 – 130, this section appears the authors did the sampling themselves

**Author’s Response:** We have deleted the entire section on “laboratory equipment and method section” as it those reflect more of prospective study design than retrospective study design. Hence, the entire paragraph is deleted.

**Reviewer’s Comment 8:** Line 157 is contradicting with line 103

**Author’s Response**: Yes it is true that I didn’t clearly explain how we came to the final dataset of 493. However, this is clearly addressed in the method section now as initial dataset underwent some data cleaning whereby 10 records were found to be either HIV or Hepatitis B positive or fall outside the specified age range targeted for this study (i.e. 18 to 63 years). Those records that fall outside this age range were excluded. Hence this gave us final dataset of 493 for haematological full blood count data and 643 for biochemical data. The sentence added to figure 1 legend is “The excluded records were from individuals whose age fall outside our specified age range of 18 to 63 years or the records that showed HIV/Hepatitis B positive, and/or Malaria positive”.

**Reviewer’s Comment on Discussion**

**Reviewer’s Comment 1:** It is not clear why the authors are comparing their study to a previous work among children less then 5 years (line 264 -266).

**Author’s Response:** The reason I compared my finding to the finding of Okebe et al. is because of the study was conducted in similar geographical setting despite difference in study population. In this study, they investigated the effect of seasonality on reference range developed in wet and dry season in children 0-5yrs. However, the comparison does not have a solid scientific merit, hence I decided to removed it.

**Reviewer’s Comment 2:** It is also not clear if the study evaluated the malaria or other parasitic infectious state of the study participants. It can be argued that, during the raining season, parasitic infection is high and can affect the haematological parameters. Therefore, the observed variation in the parameters require clear explanation.

**Author’s Response:** The study excluded not only those records that were found to have positive HIV and/or hepatitis B results but malaria positive results were also excluded. However, we did not have any positive malaria case as indicated in figure 1 and the raw data. We have updated our exclusion criteria section in our method section which has addressed this point.

**Reviewer’s Comment 3:** Why was hematocrit (HCT) not included to substantiated the hemoglobin (Hb) recorded. It is possible for people to take less fluid during the raining season and get dehydrated.

**Author’s Response:**

Seasonality is characterised by changes in temperature, humidity, rainfall, food system etc. Dehydration due to less water intake or extreme heat is also included. Hence, the primary objective is to assess the overall impact of seasonality on haematological and biochemical parameters. As such, I do not think looking at HCT is needed to substantiate the HB recorded. Ideally, we should have looked at HCT in isolation to assess the association of season explanatory variable and HCT but we focused on haematological indices directly measured instead of calculated indices by automated haematology analyser platform in our laboratory which include HB, RBC, WBC and differentials, and PLT. That’s the reason we disregarded HCT in the association study.

However, the haematocrit (HCT), which represented in the heatmap as packed cell volume (PCV), was included in the analysis for seasonal variation pattern study in figure 2 which shed light on the seasonal/monthly changes pattern of both haematological indices and biochemical measurements. The heatmap showed HCT/PCV has seasonal variation pattern in line with similar pattern observed with HB and RBC. This is further discussed in our discussion section to add more context to haemoglobin and red blood cells seasonal variation pattern results.

**Reviewer #2:**

**Reviewer’s Comment on Title and Abstract**

**Reviewer’s Comment**: Title:-if possible; the title can be changed by remove of the word (yearly), and addition of the year to (retrospective study 2018-2022)

**Author’s Response**: Addressed

**Reviewer Comment on Abstract:**
**Reviewer’s Comment 1**-Divide background from the aim of the study

**Reviewer’s Comment** 2-Better to write the objective of the study.

**Author’s Response**: Yes we have addressed both comment 1 and 2. We started the abstract with objective of the study.

**Reviewer’s Comment 3-**in abstract give brief idea about study design, study population and area, sampling, methods of data collection and the measurements (not more than 5 rows).

**Author’s Response:** Addressed

**Reviewer’s Comment 4:** briefly give the main results of the study with the accurate numbers.

**Author’s Response:** I realised some key details regarding my study was missing in the abstract. For instance, the sentence **“The median values for HB and RBC as well as liver enzymes and potassium were high in the wet season but low in dry season**” was ambiguous as we were summarising the heatmap results from our data as we showed some seasonal patterns of some laboratory parameters. This is now explained clearly in this sentence “**In addition, we observed in our heatmap result that some laboratory parameters, including HB, RBC, HCT/PCV, urea, liver enzymes, and potassium, showed seasonal patterns throughout the year, with levels being normal to slightly low during the dry season and normal to high during the wet season”.**

**Reviewer’s Comment 5**-there is some confusion regarding the effect of seasonal differences in the measurement of haematological indices and biochemical measurements.

**Author’s Response:** The study showed an association but when we further analysed the effects of seasonality after eliminating all the outliers, we found no seasonal effects as the medians from the wet season and dry season of all the haematological indices and biochemical measurements were not statistically significantly different using Wilcox test. However, what we found in this study was that we observed a seasonal patterns of haematological indices including HB, RBC, and HCT/PCV, and biochemical measurements such as liver enzymes and potassium.

Under the results section, we have re-written the results section to add more clarity to our data and eliminate any ambiguity about the results. In the result section, here is the re-written sentences “**Our study found a positive association between seasonality (wet season as the reference) and HB (HB: 0.014(0.015), P<0.05), WBC (WBC: 0.243(0.163), p =0.0014), and neutrophils (neutrophils: 0.271(0.131), P<0.05) with exception to RBC (RBC: - 0.184(0.061), P< 0.003) that showed negative association. Despite the association, the seasonal effects on our derived reference intervals from the wet season to the dry season were not statistically significant (Wilcoxon test, P>0.05). In addition, we observed in our heatmap result that some laboratory parameters, including HB, RBC, HCT/PCV, urea, liver enzymes, and potassium, showed seasonal patterns throughout the year, with median levels being normal to slightly low during the dry season and normal to high during the wet season”.**

**Comments 6**-write only the conclusion of the result. interpretation of result can be discussed in the section of discussion.
-use mean full and brief key words

**Author’s Comment**: All these points are valid and were addressed.

**Reviewer’s Comments on Methods:**
- some texts were not needed
- regarding exclusion and inclusion criteria, what about the other seasonal infections e.g. influenza
- what is the final diagnose of the healthy participants who participate in the current study.
- the biomedical history of the participants is absent from the current study.
- inclusion and exclusion criteria should minimize the un-suspected results or errors.

**Authors Response:** All these points were addressed. The complete biomedical history of patients data is not available but we have certain biomedical data such HIV, Hepatitis B, and malaria status found in raw data retrieved from the electronic database. Based on the records available, the patients with no known underlying condition, HIV and hepatitis B negative and malaria microscopy negative were included in the final dataset as shown in figure 1. However, the seasonal infections status such as influenza data was not available in their clinical biomedical data. The clinical data of the patients usually indicate any underlying condition, hence all those with underlying condition visited the lab for routine medical examination were not retrieved. Hence we focused on those sent for Routine Medical Examination/Assessment without any known underlying condition as indicated in their clinical records.

**Reviewer’s Comment on Results:**
-493 participant for haematological indexes.
-643 participant for biochemical measurements.
-the measured parameters was for separated individuals?
-643 and 493 individuals measured for different parameters.
-the current study have 2 study population.
-the study must divide into two studies.
In the results section its clearly that there was 2 study population:
-in the first one the researcher examine the seasonal haematological changes.
-in the second one the researcher examine the biochemical changes.
Then:
-there was a comparisons between the two populations.
So:
-the researcher must find the haematological indexes and biochemical measurement change in one population.
OR:
-compare the change in hematological indexes in the first population which occurs as a result of seasonal change as separate work.
-then find the seasonal changes in biochemical measurements in the second population
-finally the researcher can compare the results of the two studies

**Author’s Response:** Dear reviewer, I don’t know whether I fully comprehend your comments on separating the haematological cohort and biochemical cohort and then compare the findings. We have two separate dataset for haematological indices (493) and biochemical measurements (643) and their datasets were analysed independently because they were retrieved from two different departments in our lab. In the results section, we try to summarised the findings based on the context of the section explanation. To address your points, we have added further explanation and made some adjustments to reflect the two study populations/data, haematological indices data and biochemical measurements data.

**Reviewer’s Comment on Discussion:**
**Reviewer’s Comment 1**-in discussion there is unacceptable justification regarding the personal, socio- demographic and life style data which didn't appear in the methods of data collection in form of questionnaire or study objectives.

**Author’s Response:** Dear Reviewer, it is true that I mentioned that the COVID-19 pandemic impacted lifestyles. However, I used previously published peer-reviewed publication from Werf et al. 2021 to backed the statement that pandemic has impacted the lifestyles across many countries. I have also adjusted the paragraph to add more clarity .

**Thank you reviewer’s once again for this thoroughly resounding review. The comments were objectively laid out and I have endeavoured to address them objectively as well. Based, on these addressing these comments, I have observed that manuscript has being improved.**

**Mustapha Dibbasey**

**PhD Student**

**University of Ghana | London School of Hygiene and Tropical Med**
