# Supplementary material for: Seasonal variations in haematological and biochemical parameters of healthy Gambian adults: Retrospective study 2018–2022
Source: PLOS Glob Public Health. 2024 Sep 17;4(9):e0003715. doi: 10.1371/journal.pgph.0003715 (PMC11407651; doi:10.1371/journal.pgph.0003715)
Supplement: Attachment — Submitted filename: Authors Response Letter v2.docx [file pgph.0003715.s009.docx]

**Authors’ Responses to Editor’s and Reviewers Comments**

**Ref: Submission ID: PGPH-D-23-02498**

Title: Re: " **Seasonal and yearly variations in haematological and biochemical parameters of healthy Gambian adults: a retrospective study 2018-2022”**

**Dear Editor and Reviewers,**

First and foremost, I would like to express my gratitude for the comprehensive review process, scientifically thoughtful comments, and suggestions. These inputs will undoubtedly contribute to the enhancement of the manuscript's quality and scientific rigour. We have made a concerted effort to provide a point-by-point response to the comments and correct any typographical and phrasing errors. Additionally, we have rephrased sentences to eliminate ambiguity and removed unnecessary text.

**Editor comments**
1. Please review your reference list to ensure that it is complete and correct. If you have cited papers that have been retracted, please include the rationale for doing so in the manuscript text, or remove these references and replace them with relevant current references. Any changes to the reference list should be mentioned in the rebuttal letter that accompanies your revised manuscript. If you need to cite a retracted article, indicate the article's retracted status in the References list and also include a citation and full reference for the retraction notice.

**Response to Editor:**

Thank you for bringing to our attention that the paper by Suresh K and Chandrashekara S titled "Sample size estimation and power analysis for clinical research studies," published in the Journal of Human Reproductive Sciences in 2012, has been retracted. Referenced as **“Suresh K, Chandrashekara S. Sample size estimation and power analysis for clinical research studies. Vol. 5, Journal of Human Reproductive Sciences. 2012”.** We have replaced this retracted paper with the article by Fitzner and Heckinger from 2010, titled "Sample size calculation and power analysis: a quick review," published in Diabetes Educ. The reference is as follows: **Fitzner K, Heckinger E. Sample size calculation and power analysis: a quick review. Diabetes Educ. 2010;36(5):701-7. doi: 10.1177/0145721710380791.

Reviewer #2**

**Response to Reviewer #2 Comment on data analysis in Table 1**

Once again, I would like to thank reviewer #2 for the thorough review and for identifying the calculation error in Table 1. This was an oversight on our part. After re-examining the code used in R, we found that it was incorrectly laid out, resulting in these erroneous results. We have rectified the error in Table 1.

**Yours Sincerely**

**Mustapha Dibbasey**

**PhD Student | University of Ghana | London School of Hygiene and Tropical Med**
