# Supplementary material for: Seasonal variations in haematological and biochemical parameters of healthy Gambian adults: Retrospective study 2018–2022
Source: PLOS Glob Public Health. 2024 Sep 17;4(9):e0003715. doi: 10.1371/journal.pgph.0003715 (PMC11407651; doi:10.1371/journal.pgph.0003715)
Supplement: Attachment — Submitted filename: Author Response to Reviewer v3.docx [file pgph.0003715.s011.docx]

**Authors’ Responses to Editor’s and Reviewers Comments**

**Ref: Submission ID: PGPH-D-23-02498**

Title: Re: " **Seasonal and yearly variations in haematological and biochemical parameters of healthy Gambian adults: a retrospective study 2018-2022”**

**Dear Editor and Reviewers,**

First and foremost, I would like to express my gratitude for the comprehensive review process, scientifically thoughtful comments, and suggestions. These inputs will undoubtedly contribute to the enhancement of the manuscript's quality and scientific rigour. We have made a concerted effort to provide a point-by-point response to the comments and correct any typographical and phrasing errors. Additionally, we have rephrased sentences to eliminate ambiguity and removed unnecessary text.

**Editor comments**
1. Please review your reference list to ensure that it is complete and correct. If you have cited papers that have been retracted, please include the rationale for doing so in the manuscript text, or remove these references and replace them with relevant current references. Any changes to the reference list should be mentioned in the rebuttal letter that accompanies your revised manuscript. If you need to cite a retracted article, indicate the article's retracted status in the References list and also include a citation and full reference for the retraction notice.

**Response to Editor:**
Thank you for bringing to our attention that the paper by Suresh K and Chandrashekara S titled "Sample size estimation and power analysis for clinical research studies," published in the Journal of Human Reproductive Sciences in 2012, has been retracted. Referenced as **“Suresh K, Chandrashekara S. Sample size estimation and power analysis for clinical research studies. Vol. 5, Journal of Human Reproductive Sciences. 2012”.** We have replaced this retracted paper with the article by Fitzner and Heckinger from 2010, titled "Sample size calculation and power analysis: a quick review," published in Diabetes Educ. The reference is as follows: **Fitzner K, Heckinger E. Sample size calculation and power analysis: a quick review. Diabetes Educ. 2010;36(5):701-7. doi: 10.1177/0145721710380791.

Reviewer #2 Comments**

**Comment #1:** Regarding study participants, grouping and measurements; are still not clear

**Response to Comment #1:** I have added these sentences to add bit more clarity to study participants and measurements **‘In this study, we focused on data from individuals with no known underlying health condition who visited the clinical laboratory department for routine medical examinations and have their haematological and biochemical tests performed. After completing the retrieval process and conducting data cleaning, a total of 493 haematological full blood count results and 643 biochemical results were retrieved from the electronic database, as depicted in Figure 1. Out of the 643 biochemical results, 150 were identified as lacking corresponding haematological full blood count results in the electronic database. However, these 150 results were still included in the final analyses to increase the sample size.’**

**Study Design**: The study is a retrospective study in which we retrieved archived electronic dataset found in our hospital electronic database.

**Study population/participants:** The study focused on haematological and biochemical results data from **individuals with no known underlying health condition** who visited the clinical laboratory department for routine medical examinations and have their haematological and biochemical tests performed.

**Measurements:** both biochemical measurements and haematological indices were retrieved from the electronic database. There were 150 biochemical results retrieved from the electronic database without corresponding haematological results, but we still included them in our final biochemical dataset which resulted to 643 biochemical results.

**Reviewer’s Comment #2:** no need to mention type of analysis in the results in abstract

**Author’s Response #2:** Addressed

**Reviewer’s Comment #3:** not important (ignore this and focus on the sample that meet research criteria)

**Author’s Response #3:** Addressed

**Reviewer’s Comment #4:** focus on the objectives

**Author’s Response #4:** Addressed

**Reviewer’s Comment #5:** in the first group the researcher measure haematological parameters

**Author’s Response #5:** Here we did not measure haematological parameters instead were retrieved them from the database alongside their biochemical results. I have addressed this comment in the comment #1.

**Reviewer’s Comment #6:** mention number of participant who were in the median and mean
**Author’s Response #6:** I have addressed this by adding additional column clearly labelled in table 1. My intention is to highlight the mean, median and age range of the two datasets.

**Reviewer’s Comment #8: T**able 2 is for un-desirable date please no need for table 2 as mentioned above

**Author’s Response #8:** Table 2 has the modelling (generalised linear model) results in which we model the effects of seasonality (i.e. the samples collected in wet or dry season) on the various haematological indices and biochemical measurements. Following this modelling analysis in which we found significant effects of seasonality on certain haematological indices and biochemical measurements, we assessed the effects of seasonal variation on the reference ranges we have generated from these study datasets, haematological full blood count results and biochemical data. **I therefore believe,** Table 2 in this study addressed important section, which is integral to overall study design and idea, seasonal variations in routine laboratory parameters.

**Reviewer’s Comment #9:** a lot of repeated data and information

**Author’s Response #9:** Addressed

**Reviewer’s Comment #10:** add column for the missing data in all haematological and biochemical measurements

**Author’s Response #10:** Addressed

**Reviewer’s Comment #11:** how you compare the haematological measurements with biochemical measurements

**Author’s Response #11:** Addressed. The sentence was re-worded to add more clarity ‘**In haematological dataset, the median values for HB and RBC parameters decreased in 2020, while the median values for total WBC count, absolute neutrophils count, and platelets increased slightly. In the biochemical dataset, there was no clear pattern except for median values of AST and ALT, which showed a downward trend from 2018 to 2022’.**

My intention was not to compare the two datasets but to look for trend in the various haematological indices and biochemical measurements.

**Reviewer’s Comment #12:** use only the abbreviations for all the define term discussion

**Author’s Response #12:** Addressed

**Reviewer’s Comment #13:** you mean (using reference interval as standard?)

**Author’s Response #13:**

In this study, first and foremost we generated a reference range/interval from our study datasets because there is no current existing local reference range/interval tailor-made to Gambian population yet. Next, we assessed how the reference intervals we derived from our datasets are different in dry season and wet season,(i.e. does the lower limit and upper limit of normal changes significantly across the two seasons). Thus, the reference interval was not used as standard in this study instead we assessed the effects of seasonality on reference intervals we have generated from our haematological indices and biochemical measurements datasets.

**To addressed this comment, I re-phrased the sentence ‘The study examined the effects of seasonality on haematological indices, biochemical measurements and the reference ranges derived from our study datasets’ .**

**Reviewer’s Comment #14:** add the subtitle (limitations and recommendations)

**Author’s Response #14:** Addressed

**Reviewer’s Comment #15:** please what the effect of covid 19 in haematological and biochemical measurements

**Author’s Response #15:** I also addressed this point by deleting the paragraph as I believed it does not align with the study objectives and add no substantial information to the study. Hence deleted. **However, we found no effects on COVID-19 pandemic lockdown, which culminated into lifestyle changes in many parts of the World including the Gambia, on routine parameters.**

**Yours Sincerely**

**Mustapha Dibbasey**

**PhD Student | University of Ghana | London School of Hygiene and Tropical Medicine**
